# A Dynamic-Gain Gradient-Descent-Based Complementary Filter for Attitude Estimation

**DOI:** 10.3390/s26206475

**Published:** 2026-10-13

**Authors:** Andre Poščić, Darko Erceg, Josip Vuković, Josip Lončar

**Affiliations:** Department of Communication and Space Technologies, University of Zagreb Faculty of Electrical Engineering and Computing, Unska 3, 10000 Zagreb, Croatia; andre.poscic@fer.hr (A.P.); darko.erceg@fer.hr (D.E.); josip.vukovic@fer.hr (J.V.)

**Keywords:** sensor fusion, attitude estimation, rotation quaternion, gradient descent, complementary filter, dynamic gain, convergence, real-time systems

## Abstract

This paper presents a gradient-descent-based complementary filter with dynamically adjustable gain for attitude estimation using gyroscope, accelerometer, and magnetometer measurements. The proposed method adaptively adjusts the complementary-filter gain through a bilinear transformation of the cost function already available within the gradient-descent optimization procedure, thereby increasing the correction aggressiveness during large orientation mismatches while preserving gyroscope-dominated propagation near steady-state operation. By leveraging the already computed cost function, the proposed method avoids the need for an additional error metric or computationally demanding optimization procedure. In contrast to conventional complementary filters with fixed gain, the proposed formulation accelerates convergence from incorrect initial conditions without a substantial increase in the average arithmetic workload. Simulation, Monte Carlo, and experimental results demonstrate substantially faster convergence relative to constant-gain complementary filtering, the Madgwick algorithm, and the Mahony filter, while maintaining comparable steady-state performance. The operation-count analysis, together with average numbers of gradient-descent iterations below one in most tested configurations, demonstrates that the improved transient convergence is achieved with low average arithmetic workload, while retaining characteristics favorable for computationally constrained embedded and real-time attitude estimation systems.

## 1. Introduction

Attitude estimation plays a crucial role in a wide range of technological fields, including consumer electronics [1], human motion analysis [2,3], robotics [4], and aerospace technology [5,6,7,8]. In all these applications, accurate and reliable orientation information is essential. The achievable estimation accuracy largely depends on the selected sensors and the sophistication of the employed signal processing techniques.

In general, attitude is defined as the rotation between a body-fixed coordinate frame and a global reference frame, typically an Earth-centered inertial frame. This three-dimensional (3D) rotation can be represented using Euler angles [9] or, more commonly, quaternions [10,11]. Although Euler angles provide a more intuitive interpretation, they suffer from a singularity known as gimbal lock [12]. Quaternions, while less intuitive, avoid this issue and are therefore widely adopted in modern attitude estimation algorithms.

Numerous direct attitude estimation techniques have been proposed in the literature, including the q-method [13], ESOQ [14], QUEST [15], and recursive variants such as REQUEST and Optimal-REQUEST [16,17]. Since attitude determination typically relies on measurements from multiple sensors, sensor fusion techniques are essential for achieving accurate and robust orientation estimates. A common and cost-effective sensing solution for attitude estimation is the magnetic, angular rate, and gravity (MARG) sensor, which combines inertial sensing (tri-axis accelerometer and tri-axis gyroscope) with magnetic sensing (tri-axis magnetometer) capabilities within a single device [18,19,20,21].

Despite their practicality, each of these sensors has inherent limitations. Accelerometers measure gravitational acceleration but are also sensitive to dynamic accelerations caused by external forces. Magnetometers are susceptible to disturbances from nearby magnetic materials, including hard- and soft-iron effects, which complicate calibration and degrade measurement quality. Gyroscopes generally exhibit lower noise levels and provide angular velocity measurements that can be integrated to estimate orientation. However, they are affected by bias drift, leading to cumulative errors over time. Although none of the individual MARG sensors can provide fully reliable attitude estimates on their own, appropriate sensor fusion techniques can significantly improve performance.

Among sensor fusion approaches, Kalman-filter-based methods are widely used for attitude estimation and can provide high estimation accuracy [19,20,21]. Depending on the adopted formulation, they generally require the specification of process and measurement models and involve covariance propagation and matrix operations. Complementary filters provide an alternative approach with a simpler estimation structure and low computational requirements [22,23,24,25]. They combine the complementary characteristics of gyroscope-based orientation estimation and absolute orientation information obtained from accelerometer and magnetometer measurements.

Although several direct methods can estimate attitude using accelerometer and magnetometer data, this work focuses on a more recent iterative approach based on the gradient descent algorithm [22,26,27,28,29,30]. The gradient descent algorithm fuses accelerometer and magnetometer measurements to determine the optimal rotational quaternion by minimizing a least-squares error criterion. Several modifications of gradient-descent-based attitude estimation algorithms have subsequently been proposed to improve convergence speed, computational efficiency, and robustness to sensor disturbances. In [29], an improved formulation of the IMU/MARG gradient-descent algorithm was introduced, while [31] presented a modified formulation that improved convergence and reduced the influence of magnetic-field disturbances on roll and pitch estimation. More recently, adaptive approaches have been investigated to improve the performance of gradient-descent-based estimation under dynamic conditions [32]. In parallel, adaptive and optimized complementary-filter formulations have been proposed to alleviate the limitations associated with fixed filter parameters [33,34]. In [33], a nonlinear complementary filter was presented that combines gyroscope-based attitude propagation with vector-based attitude correction. The resulting formulation allows online gyroscope-bias estimation and provides improved stability properties. In [34], an adjustable complementary-filter gain was proposed, allowing the filter gain to be adapted according to the reliability of the accelerometer and magnetometer measurements. This adaptive approach improved the robustness of attitude estimation during dynamic motion but did not specifically aim to accelerate convergence or reduce computational complexity. In contrast, the adaptation principle proposed in this work targets faster convergence while maintaining estimation accuracy comparable to that of other algorithms, without requiring an additional optimization procedure, since the filter-gain adaptation is based on the cost function already computed within the gradient-descent procedure.

Because the gradient descent algorithm is iterative, the number of iterations becomes a critical parameter that directly influences both estimation accuracy and computational cost. An insufficient number of iterations can result in suboptimal orientation estimates, whereas an excessive number of iterations unnecessarily increases processing time and computational burden.

In real-time applications, this trade-off is particularly important, yet it is often overlooked in the literature. Some studies suggest that a single iteration may be sufficient if the convergence rate of the estimate matches or exceeds the rate of change of the object’s motion [30]. However, the required number of iterations depends on multiple factors and is difficult to determine a priori. In [35], an optimization approach for gradient descent parameter tuning in quaternion-based attitude estimation was proposed. A novel figure of merit and an associated termination criterion were introduced to automatically determine the required number of iterations. The approach presented in [35] is further extended in this work through several modifications and additions. The most important enhancement is the introduction of a dynamically adjusted complementary filter gain, which improves both convergence speed and estimation robustness compared to competing algorithms.

In this work, sequences of gyroscope, accelerometer, and magnetometer measurements are used to estimate the current orientation of an object. In the simulations, the three sensor measurement vectors in the body-fixed coordinate frame are synthetically generated, whereas in the experimental evaluation, they are obtained from actual sensors. In both cases, the corresponding gravitational acceleration and magnetic-field reference vectors expressed in the inertial frame are assumed to be known.

The remainder of the paper is organized as follows. Section 2 presents attitude representation based on Euler angles and quaternions, together with their interrelation. Section 3 outlines the mathematical framework of gradient descent-based attitude estimation and the complementary filter. Section 4 presents simulation results obtained using synthetic data and compares the proposed approach with other attitude estimation algorithms. Section 5 discusses the experimental results, while Section 6 concludes the paper and outlines directions for future work.

## 2. Attitude Representation

The orientation of a rigid body can be described as the rotation between the body-fixed coordinate frame and the North-East-Down (NED) navigation frame. A common and intuitive way to represent orientation in three-dimensional (3D) space is through Euler angles: yaw (ψ), pitch (θ), and roll (ϕ). These angles correspond to successive rotations about the *z*-, *y*-, and *x*-axes, respectively. Since matrix multiplication is not commutative, the order of rotations must be specified. In this work, the standard aerospace *z*-*y*-*x* rotation sequence is adopted.

The individual rotation matrices associated with the Euler angles are given by

(1a) $$R_{i}^{v_{1}}\left(\psi \right)=\left[\begin{array}{ccc} \cos(\psi ) & \sin(\psi ) & 0\\ -\sin(\psi ) & \cos(\psi ) & 0\\ 0 & 0 & 1 \end{array}\right],$$

(1b) $$R_{v_{1}}^{v_{2}}\left(\theta \right)=\left[\begin{array}{ccc} \cos(\theta ) & 0 & -\sin(\theta )\\ 0 & 1 & 0\\ \sin(\theta ) & 0 & \cos(\theta ) \end{array}\right],$$

(1c) $$R_{v_{2}}^{b}\left(\phi \right)=\left[\begin{array}{ccc} 1 & 0 & 0\\ 0 & \cos(\phi ) & \sin(\phi )\\ 0 & -\sin(\phi ) & \cos(\phi ) \end{array}\right].$$

Multiplying the matrices in the *z*-*y*-*x* sequence yields the direction cosine matrix (DCM)

(2) $$R_{i}^{b}\left(\phi ,\theta ,\psi \right)=R_{v_{2}}^{b}\left(\phi \right)R_{v_{1}}^{v_{2}}\left(\theta \right)R_{i}^{v_{1}}\left(\psi \right).$$

The elements of the DCM can be expressed as functions of the Euler angles

(3) $$R_{i}^{b}\left(\phi ,\theta ,\psi \right)=\left[\begin{array}{ccc} \cos(\psi )\cos(\theta ) & \cos(\theta )\sin(\psi ) & -\sin(\theta )\\ \cos(\psi )\sin(\phi )\sin(\theta )-\cos(\phi )\sin(\psi ) & \cos(\phi )\cos(\psi )+\sin(\phi )\sin(\psi )\sin(\theta ) & \cos(\theta )\sin(\phi )\\ \sin(\phi )\sin(\psi )+\cos(\phi )\cos(\psi )\sin(\theta ) & \cos(\phi )\sin(\psi )\sin(\theta )-\cos(\psi )\sin(\phi ) & \cos(\phi )\cos(\theta ) \end{array}\right].$$

Although Euler angles provide an intuitive representation of orientation, they suffer from a singularity known as gimbal lock, which results in the loss of one rotational degree of freedom. In the adopted *z*-*y*-*x* rotation sequence, this singularity occurs when θ=±90°. To avoid this limitation, att itude is commonly represented using quaternions. A quaternion extends complex numbers and consists of one real component and three mutually orthogonal imaginary components. Unlike Euler angles, quaternions represent attitude using a single rotation angle α about a unit vector $n=n_{x}\hat{i}+n_{y}\hat{j}+n_{z}\hat{k}$, where n defines the rotation axis and $\hat{i},\hat{j},\hat{k}$ are the basis vectors of the Cartesian coordinate system.

The quaternion is usually represented as a four-dimensional (4D) vector

(4) $$q=\cos\left(\frac{\alpha }{2}\right)+\sin\left(\frac{\alpha }{2}\right)n=\left[\begin{array}{c} q_{1}\\ q_{2}\\ q_{3}\\ q_{4} \end{array}\right]=\left[\begin{array}{c} \cos(\alpha /2)\\ n_{x}\sin\left(\alpha /2\right)\\ n_{y}\sin\left(\alpha /2\right)\\ n_{z}\sin\left(\alpha /2\right) \end{array}\right].$$

Because the rotation axis vector n is normalized, the resulting quaternion is also a unit quaternion satisfying ∥q∥=1. Analogous to (3), the DCM elements can also be expressed as functions of quaternion elements

(5) $$R_{i}^{b}\left(q\right)=\left[\begin{array}{ccc} q_{1}^{2}+q_{2}^{2}-q_{3}^{2}-q_{4}^{2} & 2(q_{2}q_{3}+q_{1}q_{4}) & 2(q_{2}q_{4}-q_{1}q_{3})\\ 2(q_{2}q_{3}-q_{1}q_{4}) & q_{1}^{2}-q_{2}^{2}+q_{3}^{2}-q_{4}^{2} & 2(q_{3}q_{4}+q_{1}q_{2})\\ 2(q_{2}q_{4}+q_{1}q_{3}) & 2(q_{3}q_{4}-q_{1}q_{2}) & q_{1}^{2}-q_{2}^{2}-q_{3}^{2}+q_{4}^{2} \end{array}\right]$$

Unlike the Euler-angle-based formulation in (3), the quaternion-based DCM formulation in (5) involves only algebraic operations on quaternion elements and does not require trigonometric function evaluations. Consequently, quaternion-based approaches are particularly well suited for low-cost and low-power embedded systems with limited computational resources.

By equating the elements of (3) and (5), Euler angles can be expressed as functions of quaternion elements, with the quadrant ambiguity of the yaw and roll angles resolved using the two-argument inverse tangent function atan2.

(6) $$\left[\begin{array}{c} \psi \\ \theta \\ \phi \end{array}\right]=\left[\begin{array}{c} \begin{array}{c} atan2(r_{12},r_{11}) \end{array}\\ -\arcsin(r_{13})\\ \begin{array}{c} atan2(r_{23},r_{33}) \end{array} \end{array}\right]=\left[\begin{array}{c} \begin{array}{c} atan2\left(2\left(q_{2}q_{3}+q_{1}q_{4}\right),q_{1}^{2}+q_{2}^{2}-q_{3}^{2}-q_{4}^{2}\right) \end{array}\\ \begin{array}{c} -\arcsin\left(2\left(q_{2}q_{4}-q_{1}q_{3}\right)\right) \end{array}\\ \begin{array}{c} atan2\left(2\left(q_{3}q_{4}+q_{1}q_{2}\right),q_{1}^{2}-q_{2}^{2}-q_{3}^{2}+q_{4}^{2}\right) \end{array} \end{array}\right]$$

## 3. Gradient-Descent-Based Complementary Filter for Attitude Estimation

### 3.1. Attitude Estimation Based on Gradient Descent Algorithm

One of the attitude estimation algorithms considered in this work is the gradient descent algorithm. The gradient descent algorithm is an iterative optimization technique commonly used to minimize a given cost function *J*. In the context of attitude estimation, the cost function is formulated as a function of the rotation quaternion. The gradient descent procedure can be written as a straightforward recursive update equation [35]

(7) $$q_{k+1}=q_{k}-\mu \nabla J\left(q_{k}\right).$$

Here, q denotes the rotation quaternion, and μ represents the step size, which determines the convergence rate of the algorithm. If μ is chosen too small, convergence becomes slow and the algorithm requires many iterations to approach the minimum. Conversely, if μ is too large, the algorithm may become unstable and diverge. For this reason, the step size μ is typically selected empirically.

The gradient descent procedure begins with an initial estimate $q_{0}$, which is iteratively updated at each step *k* as the estimate converges toward the minimum of the cost function. After each iteration, the updated quaternion is normalized in order to preserve the unit-quaternion constraint.

(8) $$q_{k+1}\leftarrow \frac{q_{k+1}}{∥q_{k+1}∥}$$

Without normalization, the resulting quaternion no longer represents a pure rotation, and the corresponding transformation matrix may introduce undesired scaling effects. The gradient $\nabla J(q_{k})$ is computed as an unconstrained gradient in $R^{4}$, whereas the optimization variable is constrained to the unit sphere $S^{3}$. The unconstrained gradient may contain a radial component that affects the magnitude of the estimated quaternion. Therefore, the normalization applied after each gradient-descent step in (8) projects the updated quaternion back onto $S^{3}$, thereby enforcing the unit-quaternion constraint and removing the radial scaling introduced by the unconstrained update.

The cost function is therefore defined as $J\left(q\right):R^{4}\to R$. To formulate this cost function explicitly, we first introduce the corresponding error vectors. Let $a^{b}$ denote the gravitational acceleration vector expressed in the body-fixed frame, and let $R_{i}^{b}$ represent the rotation matrix describing the transformation from the inertial frame to the body frame. Ideally, if $R_{i}^{b}$ is constructed from the true rotation quaternion, the body-frame acceleration vector can be obtained as $a^{b}=R_{i}^{b}a^{i}$, where $a^{i}$ is the reference gravitational acceleration vector expressed in the inertial frame. However, if the quaternion used to form $R_{i}^{b}$ does not accurately represent the body’s orientation, a discrepancy arises between the true and predicted vectors. In that case, the error vector $e_{a}$ is defined as [35]

(9) $$e_{a}=R_{i}^{b}a^{i}-a^{b}.$$

Analogously, the magnetic field error vector is defined as [35]

(10) $$e_{m}=R_{i}^{b}m^{i}-m^{b}.$$

Here, vectors $a^{i}$ and $m^{i}$ denote the known reference vectors of gravitational acceleration and the Earth’s magnetic field, respectively, both expressed in the inertial reference frame. It should be noted that the vectors $a^{i}$, $a^{b}$, $m^{i}$ and $m^{b}$ are not unit vectors when they are measured and should be normalized before substituting into Equations (9) and (10). Based on (9) and (10), the cost function J(q) is then defined as follows [35]:

(11) $$J\left(q\right)=e_{a}^{T}e_{a}+e_{m}^{T}e_{m}.$$

It should be noted that under ideal, noise-free conditions, the cost function is equal to zero when the quaternion corresponds to the true attitude of the object, since both error vectors vanish. In practical scenarios, however, measurement noise and sensor imperfections prevent the error vectors from becoming exactly zero. Although these disturbances cannot be completely removed, the cost function can still be minimized using the gradient descent algorithm. To implement the gradient descent procedure, it is necessary to derive an explicit expression for the gradient of the cost function. This requires computing the partial derivatives of the cost function (11) with respect to the *i*-th element of the rotation quaternion [35]

(12) $$\frac{\partial }{\partial q_{i}}J\left(q\right)=2e_{a}^{T}\left(\frac{\partial }{\partial q_{i}}R_{i}^{b}\right)a^{i}+2e_{m}^{T}\left(\frac{\partial }{\partial q_{i}}R_{i}^{b}\right)m^{i}$$

where $\partial R_{i}^{b}/\partial q_{i}$ is the partial derivative of the rotation matrix $R_{i}^{b}$ (5) with respect to the *i*-th element of the rotation quaternion, which yields the matrices $M_{i}$ [35].

(13a) $$M_{1}=\frac{\partial }{\partial q_{1}}R_{i}^{b}=2\left[\begin{array}{ccc} q_{1} & q_{4} & -q_{3}\\ -q_{4} & q_{1} & q_{2}\\ q_{3} & -q_{2} & q_{1} \end{array}\right]$$

(13b) $$M_{2}=\frac{\partial }{\partial q_{2}}R_{i}^{b}=2\left[\begin{array}{ccc} q_{2} & q_{3} & q_{4}\\ q_{3} & -q_{2} & q_{1}\\ q_{4} & -q_{1} & -q_{2} \end{array}\right]$$

(13c) $$M_{3}=\frac{\partial }{\partial q_{3}}R_{i}^{b}=2\left[\begin{array}{ccc} -q_{3} & q_{2} & -q_{1}\\ q_{2} & q_{3} & q_{4}\\ q_{1} & q_{4} & -q_{3} \end{array}\right]$$

(13d) $$M_{4}=\frac{\partial }{\partial q_{4}}R_{i}^{b}=2\left[\begin{array}{ccc} -q_{4} & q_{1} & q_{2}\\ -q_{1} & -q_{4} & q_{3}\\ q_{2} & q_{3} & q_{4} \end{array}\right]$$

Combining (12) and (13), the gradient of the cost function evaluated at the current quaternion estimate $q_{k}$ can be written as [35]

(14) $$\nabla J\left(q_{k}\right)=2{\left[\begin{array}{c} e_{a}^{T}M_{1}a^{i}+e_{m}^{T}M_{1}m^{i}\\ e_{a}^{T}M_{2}a^{i}+e_{m}^{T}M_{2}m^{i}\\ e_{a}^{T}M_{3}a^{i}+e_{m}^{T}M_{3}m^{i}\\ e_{a}^{T}M_{4}a^{i}+e_{m}^{T}M_{4}m^{i} \end{array}\right]}_{q=q_{k}}$$

The effectiveness of the gradient descent algorithm for attitude estimation is influenced by several key parameters, including the step size μ, the maximum number of iterations $N_{max}$, and the choice of the initial quaternion $q_{0}$. In addition, particular attention is given to the formulation of an appropriate termination criterion, as well as to different strategies for propagating the initial quaternion. Achieving optimal performance requires selecting a parameter set that ensures high estimation accuracy while minimizing the number of iterations required for convergence. The iterative process of the gradient descent algorithm is terminated either when the maximum number of iterations $N_{\max}$ is reached or when the selected convergence criterion is satisfied. In its basic form, the convergence criterion is defined as

(15) $${|\Delta J\left(q\right)}_{k}{|=|J\left(q\right)}_{k}-J{\left(q\right)}_{k-1}|\;<\;\Delta J_{\max},$$

where $\Delta J_{\max}$ is the maximum allowable difference between consecutive cost function values. For the initial iteration, the previous cost function value $J{\left(q\right)}_{k-1}$ is unavailable. Therefore, it is initialized to zero, yielding ${|\Delta J\left(q\right)}_{0}{|=J\left(q\right)}_{0}$. This initialization provides a large initial value of ΔJ when the estimate is far from the minimum, while simultaneously allowing rapid termination when the initial estimate is already close to the optimum. Parameters $N_{max}$ and $\Delta J_{\max}$ are user-defined parameters. To avoid premature termination near stationary points with small changes in the cost function but relatively large cost function values, the convergence criterion can be strengthened by the additional requirement

(16) $${|\Delta J\left(q\right)}_{k}|\;<\;\Delta J_{\max}\;\wedge \;J{\left(q\right)}_{k}<J_{\max},$$

where $J_{\max}$ denotes the maximum acceptable value of the cost function. Thus, $J_{\max}$ does not define an independent termination condition, but rather acts as an additional constraint on the cost-function-change criterion. The parameter $J_{\max}$ is empirically selected in the same manner as $N_{\max}$ and $\Delta J_{\max}$.

### 3.2. Complementary Filter Based on Gradient Descent Algorithm

A complementary filter is a widely used sensor-fusion technique that combines measurements from absolute sensors, such as accelerometers and magnetometers, with measurements from rate sensors, such as gyroscopes. Measurements acquired from the accelerometer and magnetometer are used in the gradient-descent algorithm. Although these measurements are affected by relatively high levels of noise, they provide an absolute orientation reference and therefore contribute to the long-term accuracy of the estimate. In contrast, numerical integration of the gyroscope angular velocity provides a smooth and accurate short-term orientation estimate, but is subject to accumulating drift caused by gyroscope errors.

By combining low-pass and high-pass filtering actions, the complementary filter exploits the advantages of each sensor type while mitigating their respective drawbacks. Low-pass filtering of the gradient-descent-based estimate attenuates high-frequency measurement noise while maintaining long-term accuracy. In contrast, high-pass filtering of the gyroscope-based estimate retains the low-noise characteristics resulting from angular-velocity integration while suppressing the slowly accumulating drift. Owing to its simplicity and low computational burden, the complementary filter is particularly suitable for low-power and resource-constrained applications. In practice, the complementary filter combines orientation estimates obtained from different sensors through a weighted combination. For instance, the rotation quaternion can be estimated from accelerometer and magnetometer measurements using the gradient descent algorithm described previously. Let this estimate be denoted by $q_{\mathrm{gd}}$. Alternatively, the quaternion can be derived from gyroscope angular velocity measurements through integration. This estimate is denoted by $q_{\omega }$. The complementary filter then forms a weighted combination of these two estimates, which can be expressed as

(17) $$q=Kq_{\omega }+\left(1-K\right)q_{\mathrm{gd}},$$

where *K* represents the scaling factor or the complementary filter gain, which determines the level of confidence assigned to each estimation method. Here, q represents the fused estimate of the rotation quaternion. It should be noted that directly summing quaternions does not, in general, preserve their unit norm. Nevertheless, this approach is acceptable when the quaternions being combined are close to each other in quaternion space. Even so, it is recommended to normalize the resulting quaternion to preserve the unit-quaternion constraint. The gyroscope-based quaternion estimate $q_{\omega }$ is obtained through numerical integration as

(18) $$q_{k}=q_{k-1}+\Delta T{\dot{q}}_{k}.$$

Here, ΔT is the difference in time between two samples and ${\dot{q}}_{k}$ is the time derivative of the rotation quaternion in step *k*. ${\dot{q}}_{k}$ can be obtained from the angular velocity using the following equation [36]:

(19) $$\dot{q}=\frac{d}{dt}q=\frac{1}{2}\Omega \left(\omega \right)q.$$

The matrix Ω(ω) is defined as [36]

(20) $$\Omega \left(\omega \right)=\left[\begin{array}{cccc} 0 & -\omega_{x} & -\omega_{y} & -\omega_{z}\\ \omega_{x} & 0 & \omega_{z} & -\omega_{y}\\ \omega_{y} & -\omega_{z} & 0 & \omega_{x}\\ \omega_{z} & \omega_{y} & -\omega_{x} & 0 \end{array}\right].$$

Although integration of gyroscope measurements suppresses high-frequency noise, gyroscope bias introduces drift in the estimated quaternion over time. Consequently, the gyroscope-based estimate $q_{\omega }$ is effectively high-pass filtered within the complementary filter structure. In contrast, the quaternion estimate $q_{gd}$ obtained from accelerometer and magnetometer measurements provides absolute attitude information but is more sensitive to measurement noise and disturbances. Therefore, it is low-pass filtered. The resulting complementary filter combines the short-term reliability of gyroscope integration with the long-term reference provided by the accelerometer and magnetometer.

The third parameter mentioned at the end of Section 3.1 is the initial quaternion $q_{0}$ and its propagation. In [35], three different methods of initial quaternion propagation were compared and the most effective propagation method was called predictive propagation of initial quaternion. The same propagation technique was adopted by [37]. The propagation process used in this method can be described as follows: in each iteration the quaternion estimated in the previous time step and the measured angular velocity are used to approximate the rotation quaternion in the current time step. This quaternion is then used as the initial quaternion for the gradient descent algorithm. This initialization strategy improves convergence behavior by providing an initial quaternion estimate that is already close to the optimal solution at the current time step. Therefore, this propagation strategy is also adopted in the proposed approach.

### 3.3. Dynamic Calculation of Complementary Filter Gain

In many complementary filter implementations, the complementary filter gain *K* is chosen as a constant parameter. For example, when K=0.8, the fused rotation quaternion estimate is dominated by the gyroscope-based quaternion estimate, while less weight is assigned to the quaternion obtained using the gradient descent algorithm. In this work, such an approach is referred to as the Gradient-Descent-Based Complementary Filter with Constant Gain (GDCF-CG).

The main limitation of the GDCF-CG method is related to the trade-off between transient response and noise suppression. When a large value of *K* is selected, the gyroscope-based estimate dominates the fused quaternion. Although this improves short-term smoothness and reduces the influence of measurement noise, the filter requires a longer transient phase to converge toward the true attitude because the gradient-descent-based estimate, which provides absolute orientation information, has only a limited influence on the fused solution.

Conversely, when a smaller value of *K* is used (e.g., K=0.1), the contribution of the gradient-descent-based estimate becomes dominant. In this case, the transient phase is significantly shortened because the filter converges more rapidly toward the absolute orientation estimate. However, the resulting attitude estimate becomes more sensitive to measurement noise and disturbances originating from accelerometer and magnetometer measurements.

To mitigate the drawbacks of the GDCF-CG method, this work proposes a modified approach referred to as the Gradient-Descent-Based Complementary Filter with Dynamic Gain (GDCF-DG). In the proposed method, the complementary filter gain *K* is dynamically adjusted during the attitude estimation process. The primary objective of this approach is to preserve the advantages of both estimation methods while simultaneously reducing the duration of the transient phase.

The key idea behind the proposed method is to adapt the complementary filter gain according to the current value of the cost function J(q). Since the cost function reflects the discrepancy between the estimated and reference sensor vectors, its value can be interpreted as a measure of the deviation of the estimated attitude from the true attitude. Large values of J(q) indicate significant attitude estimation error, whereas values approaching zero correspond to estimates that are close to the true orientation.

This observation provides a natural mechanism for dynamically adjusting the complementary filter gain. When the value of J(q) is large, greater confidence should be assigned to the gradient-descent-based estimate in order to accelerate convergence toward the true attitude, implying K(q)→0. Conversely, when J(q) becomes small, the transient phase is effectively completed and greater confidence can be assigned to the gyroscope-based estimate, implying K(q)→1.

A potential concern associated with the weighted sum in (17) is the linear combination of two different quaternion estimates, $q_{\omega }$ and $q_{\mathrm{gd}}$. Such a linear combination provides a meaningful approximation when the two quaternion estimates are sufficiently close in quaternion space. During the transient phase, however, the two estimates may be far apart, potentially resulting in a poor approximation of the actual attitude. The proposed GDCF-DG algorithm mitigates this issue because $K_{d}\to 0$ for large attitude discrepancies, causing the fused estimate to rely primarily on the gradient-descent-based estimate $q_{\mathrm{gd}}$ during the transient phase.

Furthermore, the linear combination of $q_{\omega }$ and $q_{\mathrm{gd}}$ may result in partial or complete cancellation if the two estimates converge to q and −q, respectively, since these quaternions describe the same physical attitude. This ambiguity can be detected by evaluating the quaternion inner product, since $q_{\omega }^{T}q_{\mathrm{gd}}<0$ indicates that the two quaternions lie in opposite hemispheres of $S^{3}$. In the proposed GDCF-DG algorithm, this possibility is mitigated through predictive propagation of the initial quaternion [35], whereby the gyroscope-based estimate $q_{\omega }$ is used as the initial quaternion for the gradient-descent procedure. Consequently, the gradient-descent estimate is initialized in the same quaternion hemisphere as the gyroscope-based estimate and, for sufficiently small gradient-descent steps, is expected to remain in the same hemisphere. Nevertheless, when quaternion estimates are combined linearly, it is generally advisable to enforce a consistent quaternion sign, such that $q_{\omega }^{T}q_{\mathrm{gd}}>0$, in order to avoid cancellation.

To construct the proposed dynamic gain function, it is first necessary to determine the range of the cost function J(q). Consider the acceleration-related term from (11)

(21) $$e_{a}^{T}e_{a}={\left(R_{i}^{b}a^{i}-a^{b}\right)}^{T}\left(R_{i}^{b}a^{i}-a^{b}\right).$$

Since $R_{i}^{b}$ is a rotation matrix and both $a^{i}$ and $a^{b}$ are normalized vectors, straightforward manipulation yields

(22) $$e_{a}^{T}e_{a}=2-2{\left(R_{i}^{b}a^{i}\right)}^{T}a^{b}.$$

The term ${\left(R_{i}^{b}a^{i}\right)}^{T}a^{b}$ represents the scalar product of two unit vectors and can therefore be written as cosγ, where γ is the angle between them. Consequently,

(23) $$e_{a}^{T}e_{a}=2\left(1-\cos\gamma \right).$$

Because cosγ∈[−1,1], it follows that $e_{a}^{T}e_{a}\in \left[0,4\right]$. An analogous result holds for the magnetic-field-related term, yielding $e_{m}^{T}e_{m}\in \left[0,4\right]$. Hence, from (11), it follows that

(24) J(q)∈[0,8].

Although the cost function J(q) is formally bounded within the interval [0,8], its practically relevant values during the convergence process may span several orders of magnitude. In particular, after convergence the cost function often reaches values on the order of $10^{-3}$ or lower, whereas substantially larger values occur during the transient phase. Consequently, a simple linear mapping between J(q) and the complementary filter gain would provide insufficient sensitivity in the low-cost-function region, where accurate adjustment of the gain is the most critical. Therefore, it is necessary to introduce a nonlinear transformation that maps the cost function value J(q)∈[0,8] into the interval [0,1], corresponding to the desired range of the complementary filter gain. In addition, the transformation should preserve the inverse relationship between the gain coefficient and the cost function value while remaining computationally efficient for real-time implementation.

A bilinear transformation of the form

(25) $$\frac{J\left(q\right)-K_{0}}{J\left(q\right)+K_{0}}$$

naturally satisfies these requirements, where $K_{0}>0$ is a user-defined parameter. However, this transformation maps the cost function into the interval [−1,1], which does not correspond to the desired range of the complementary filter gain. Therefore, the following modified transformation is introduced:

(26) $$K_{d}=1-\frac{1}{2}\left(\frac{J\left(q\right)-K_{0}}{J\left(q\right)+K_{0}}+1\right)=\frac{K_{0}}{J\left(q\right)+K_{0}}.$$

With the complementary filter gain defined in this way, the balance between the gyroscope-based and gradient-descent-based estimates is dynamically adjusted according to the current value of the cost function. It should be noted that this formulation may present a potential limitation in real-world scenarios. Non-gravitational accelerations and disturbances in the local magnetic field may increase the value of the cost function J(q), thereby reducing $K_{d}$ and increasing the weight assigned to the gradient-descent-based estimate derived from the disturbed accelerometer and magnetometer measurements. This potential effect was investigated through the experimental validation described in Section 5 and was not found to have a significant influence on the estimation performance.

From (26), the interpretation of the parameter $K_{0}$ becomes straightforward. When $K_{0}=J\left(q\right)$, it follows that $K_{d}=0.5$, meaning that equal confidence is assigned to the gradient-descent-based estimate and the gyroscope-based estimate. Thus, $K_{0}$ defines the cost function value corresponding to equal weighting of the gyroscope-based and gradient-descent-based estimates.

**Figure 1:**
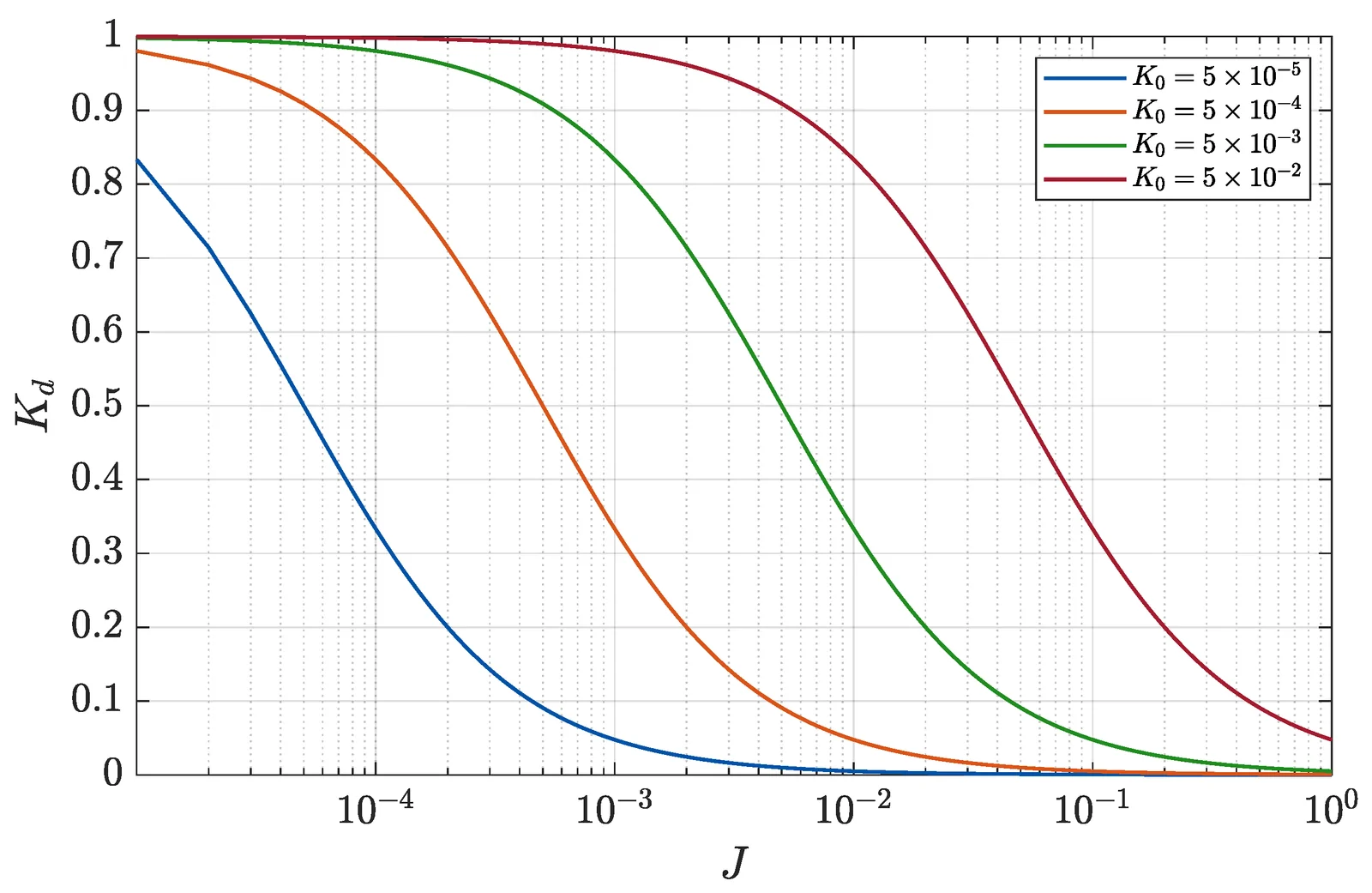
Dynamic gain $K_{d}$ as a function of the cost function J(q) for different values of $K_{0}$.

Examples of the dependence of the dynamic complementary filter gain $K_{d}$ on the cost function J(q) for different values of $K_{0}$ are shown in Figure 1. The parameter $K_{0}$ depends on the characteristics and quality of the employed sensors. Nevertheless, it can be determined using the design procedure described below.

Once the filter has converged, the cost function no longer decreases systematically but fluctuates around a residual level determined by the quality of the sensor measurements. Let $\bar{J}$ denote the mean value of the cost function under steady-state operation. Approximating the mean dynamic gain by evaluating (26) at $\bar{J}$ gives

(27) $$\bar{K_{d}}\approx \frac{K_{0}}{\bar{J}+K_{0}}.$$

A complementary filter is typically designed by specifying the desired weighting of the gyroscope-based estimate, that is, by selecting the constant gain *K* in (17). Requiring the proposed filter to reproduce approximately the same weighting under steady-state operation, $\bar{K_{d}}\approx K$, and solving (27) for $K_{0}$ yields

(28) $$K_{0}=\frac{K}{1-K}\;\bar{J}.$$

The selection of $K_{0}$ is therefore reduced to the determination of a single scalar, the residual level $\bar{J}$. Rather than being tuned independently, $K_{0}$ is determined from the conventional complementary-filter gain *K* and the residual level associated with the particular measurement setup. Consequently, its numerical value may differ between scenarios even when the desired steady-state filter weighting remains the same.

**Figure 2:**
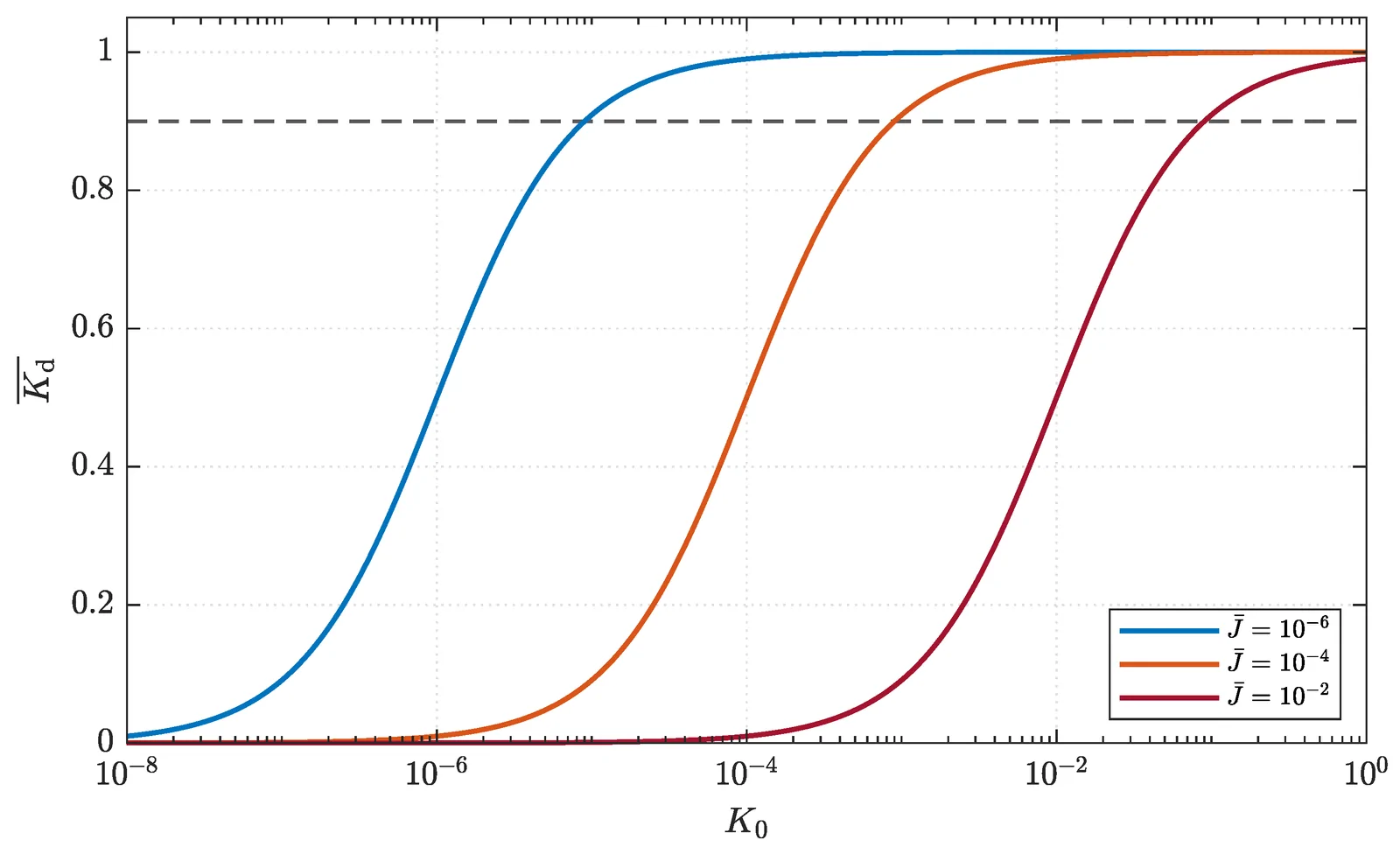
Mean gain $\bar{K_{d}}$ as a function of $K_{0}$, shown for several residual levels $\bar{J}$. The dashed line marks the design value K=0.90; its intersection with each curve is the value of $K_{0}$ returned by (28).

Figure 2 illustrates this relationship for several residual levels. The residual level $\bar{J}$ can be determined directly from recorded measurements. The constant-gain filter in (17) is run once over a representative measurement segment using the desired gain *K* and the selected termination criterion $\Delta J_{\max}$. After the initial transient has decayed, $\bar{J}$ is obtained as the average value of J(q), and $K_{0}$ then follows from (28). No reference attitude is required, since J(q) is evaluated directly from the sensor measurements and is already computed at each time step as part of the gradient-descent procedure.

The magnitude of $\bar{J}$ reflects the quality of the measurements. Non-gravitational accelerations, magnetic disturbances, and calibration residuals may increase it because, under such conditions, no attitude necessarily satisfies both vector measurements simultaneously. The residual level also depends on how far the gradient-descent procedure is allowed to proceed before termination. Therefore, $\bar{J}$, and consequently $K_{0}$, is determined for the selected $\Delta J_{\max}$ and should be reevaluated using (28) if the termination criterion is changed. The mean gain obtained using this procedure is verified using the simulation and experimental results presented in Section 4 and Section 5.

### 3.4. Program Implementation and Computational Cost

**Figure 3:**
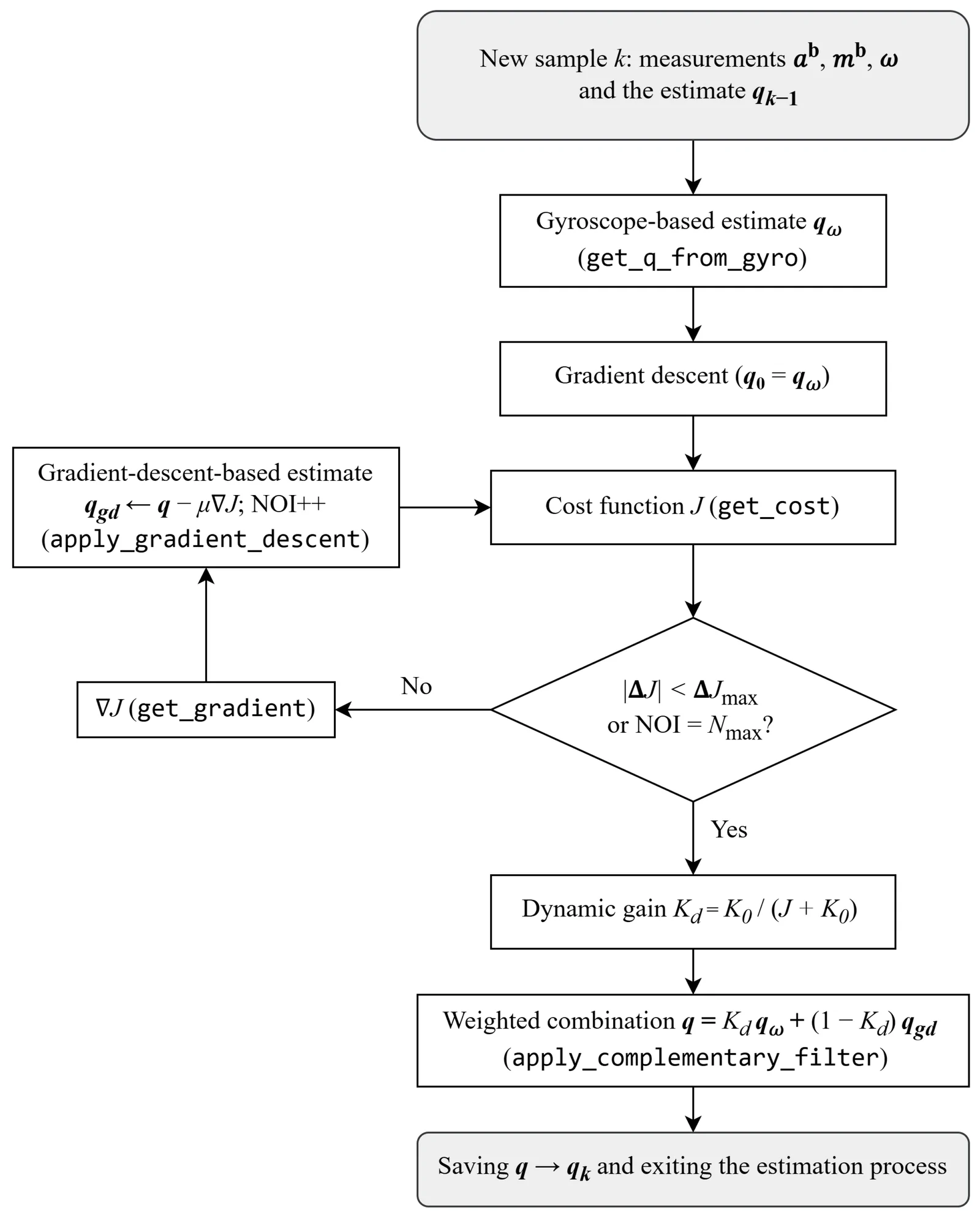
Processing flow for a single sample of the proposed algorithm. The gradient descent loop terminates when either the termination criterion is satisfied or the maximum number of iterations, $N_{\max}$, is reached. The cost function value *J* computed within the loop is directly used to determine the dynamic gain $K_{d}$.

The algorithm was implemented in MathWorks MATLAB R2024a, with the estimate from the previous sample, $q_{k-1}$, retained between successive calls, as required by a recursive filter. The processing of a single sample follows the procedure shown in Figure 3. The estimate $q_{\omega }$ is first obtained from the angular-rate measurements by numerical integration and is then used as the initial quaternion for the gradient-descent loop, which returns $q_{\mathrm{gd}}$ together with the corresponding cost-function value *J*. The two estimates are subsequently fused, with the gain $K_{d}$ computed from $K_{0}$ and the value of *J* already available from the gradient-descent loop, so that no additional cost-function evaluation is required.

The proposed algorithm together with the Madgwick [30,38], Mahony [33], and GDCF-CG [35] reference algorithms were implemented as separate MATLAB classes with identical input and output interfaces. Each algorithm receives the measurements $a^{b}$, $m^{b}$, and ω for a single sample and returns the estimated attitude quaternion. In every simulation and experiment, all algorithms are executed within the same script using an identical measurement sequence and the same initial attitude estimate, and their outputs are processed using the same routines. This provides a consistent basis for the comparisons presented in Section 4 and Section 5.

The algorithmic computational workload is quantified by the number of arithmetic operations per sample, counted separately for multiplications, additions and subtractions, divisions, and square roots. These operation types are not combined into a single metric because divisions and square roots generally require more computational effort than multiplications and additions, with their relative costs depending on the processor architecture and implementation. All four algorithms are evaluated using the same counting convention: squaring and multiplication by a constant each count as one multiplication; a squared term or constant multiple used repeatedly within a single step (e.g., $q_{1}^{2}$ or $2q_{1}$) is computed only once; matrix–vector products are counted element by element, including multiplications by zero-valued entries; and normalization of an *n*-component vector requires 2n multiplications, n−1 additions, one square root, and one division. Comparisons are not included in the operation count. The resulting counts depend on the arrangement of the expressions in the implementation, and all four algorithms may be further reduced through standard code optimization.

The cost-function and gradient rows each include the computation of the rotation matrix R(q). For each operation type considered separately, the corresponding number of operations per sample can be expressed as

(29) $$C\left(\mathrm{NOI}\right)=C_{\mathrm{fix}}+\mathrm{NOI}\cdot C_{\mathrm{iter}},$$

where $C_{\mathrm{fix}}$ denotes the number of operations of the considered type required by the steps executed once per sample, including one cost-function evaluation, while $C_{\mathrm{iter}}$ denotes the corresponding number required by the four steps repeated during each gradient-descent iteration (Table 1). The expression is exact for $\mathrm{NOI}<N_{\max}$. When $\mathrm{NOI}=N_{\max}$, the final cost-function evaluation associated with the termination check is not performed, and (29) therefore represents an upper bound. Table 2 compares the operation counts of GDCF-CG and GDCF-DG with those of the Madgwick and Mahony algorithms, which perform a single correction step per sample and consequently have fixed operation counts.

For the experimental configuration with $k_{I}=0.3$ detailed in Section 5, the Mahony filter requires 111 multiplications and 68 additions/subtractions per sample, while the numbers of divisions and square roots remain unchanged.

At NOI=0, the proposed algorithm requires approximately the same number of multiplications and additions as the Mahony filter and fewer than the Madgwick algorithm. Each additional gradient-descent iteration increases the operation count by $C_{\mathrm{iter}}$, with the gradient evaluation accounting for the largest contribution. The predictive propagation ($q_{0}=q_{\omega }$) and the adaptive termination criterion limit this additional workload by allowing the gradient-descent procedure to terminate with NOI=0 whenever further correction is unnecessary. The average NOI obtained in each scenario is reported in Section 4 and Section 5, while the maximum per-sample workload is bounded by $N_{\max}$. The dynamic-gain calculation itself requires only one additional addition and one division per sample. Consequently, the difference in arithmetic-operation count between GDCF-DG and GDCF-CG is determined primarily by their respective NOI values.

## 4. Simulations of Gradient-Descent-Based Complementary Filter with Dynamic Gain

The proposed GDCF-DG algorithm does not assume any specific model of the object’s rotational motion. The orientation is estimated recursively from the current sensor measurements and the attitude estimate from the previous sample, without assuming a predefined evolution of the angular velocity. Consequently, the object may remain stationary or undergo rotational motion with constant or time-varying angular velocity. The algorithm is evaluated in this work under static and constant-angular-velocity conditions using synthetically generated data, while the experimental evaluation additionally considers manually induced rotational motion.

The convergence performance of the proposed GDCF-DG is evaluated through a series of simulation studies and compared with the constant-gain formulation (GDCF-CG), the Madgwick algorithm, and the Mahony explicit complementary filter. The Madgwick algorithm [30] is used as the reference benchmark because of its widespread adoption in low-cost attitude estimation systems. Its tuning parameter β controls the trade-off between gyroscope drift compensation and sensitivity to accelerometer and magnetometer noise. The Mahony explicit complementary filter [33] is included as a second reference because it represents an established nonlinear complementary-filter formulation directly on SO(3) and, unlike the Madgwick algorithm, applies a correction whose magnitude depends on the attitude error. Its proportional gain $k_{P}$ controls the strength of the attitude-error correction and therefore influences the trade-off between correction responsiveness and sensitivity to measurement noise. The gyroscope-bias integral gain $k_{I}$ of [33] is set to zero throughout the simulations because the synthetically generated measurements contain no gyroscope bias. The corresponding gyroscope-bias compensation parameter ζ of the Madgwick algorithm is set to zero for the same reason. The specific values of β and $k_{P}$ used in each simulation scenario are provided in the corresponding subsection.

All algorithms and simulations are implemented in MATLAB. The algorithms receive body-frame sensor measurements as inputs and return an estimated rotation quaternion, from which the corresponding rotation matrix is obtained using (5).

The reference vectors used in the inertial frame are the gravitational acceleration vector $a^{i}={\left[0,\;0,\;9.81\right]}^{T}$ m/s^2^ and the magnetic field vector $m^{i}={\left[22.2,\;1.7,\;42.7\right]}^{T}$ μT. The magnetic field vector was obtained from the World Magnetic Model [39] for the geographical coordinates of Zagreb, Croatia ($45{}^{\circ}48^{\prime }26^{\prime \prime }$ N, $15{}^{\circ}58^{\prime }3^{\prime \prime }$ E).

The sensor measurements used in the simulations are synthetically generated from a known ground-truth rotation quaternion. In the static scenario, the true quaternion remains constant throughout the simulation. In the dynamic scenario, the true quaternion is generated by rotating the object about a predefined axis with constant angular velocity.

The simulations are conducted under both ideal noiseless conditions and noisy conditions. In the noisy case, additive Gaussian white noise is applied to the synthetically generated sensor measurements.

The noise characteristics are derived from experimental measurements obtained using a low-cost MPU9250 MARG sensor by TDK InvenSense, San Jose, CA, USA. More than 3500 samples were recorded while the sensor remained stationary.

For each sensor, the highest measured variance among all axes was selected and used in the simulations. The resulting noise variances are $0.2\times 10^{-3}$ (m/s^2^)^2^ for the accelerometer, 0.65 (μT)^2^ for the magnetometer, and $0.35\times 10^{-6}$ (rad/s)^2^ for the gyroscope.

Since the sensor measurements are synthetically generated and the true quaternion is known at every time step, the estimation accuracy can be evaluated using the principal angle error (PAE). The PAE is computed from the corrective rotation matrix $R^{T}\hat{R}$, where R and $\hat{R}$ denote the true and estimated rotation matrices, respectively. For each sample, the PAE is calculated as

(30) $$\mathrm{PAE}=\arccos\left[\frac{1}{2}\left(\mathrm{Tr}(R^{T}\hat{R})-1\right)\right].$$

Following the optimization procedure presented in [35], the gradient descent parameters are set to their optimized values. The gradient descent step size is chosen as μ=0.07, and the change in the cost function value, $\Delta J_{\max}$, is used as the primary termination criterion. The specific value of $\Delta J_{\max}$ is stated separately for each simulation scenario. If the convergence criterion is not satisfied, a secondary termination condition of $N_{\max}=20$ iterations is applied. The termination parameter $J_{\max}$ was not used in these simulations.

The sampling frequency is $f_{s}=10$ Hz in all simulations. The record length is T=1000 s for the dynamic scenario and T=200 s for the static scenario, providing sufficient time for all considered algorithms to reach steady state after the initial transient.

### 4.1. Static Noiseless Simulations

The convergence behavior of the considered algorithms is most clearly observed under static conditions, where the observed object remains stationary and the initial rotation angle α from (4) is offset by 180° relative to the true orientation. If the initial quaternion coincides with the true quaternion, the transient response vanishes entirely. Consequently, an initial offset of 180° represents the worst-case convergence scenario.

However, the exact antipodal configuration exhibits a particular numerical behavior that must be taken into account. To understand this behavior, consider the individual terms of the cost function. From (23), each contribution to the cost function can be expressed as a function of the angle γ between the estimated and measured vectors:

(31) $$J_{k}\left(\gamma_{k}\right)=2-2\cos\gamma_{k}=4\sin^{2}\frac{\gamma_{k}}{2},$$

where the index *k* denotes the *k*-th sensor (k=a for the accelerometer, k=m for the magnetometer). By differentiating with respect to the error angle, we obtain:

(32) $$\frac{dJ_{k}}{d\gamma_{k}}=2\sin\gamma_{k}.$$

For $\gamma_{k}=0$ (correct orientation), the cost function reaches its global minimum $J_{k}=0$, while for $\gamma_{k}=\pi$ (antipodal configuration) it reaches its maximum $J_{k}=4$. In both cases, the derivative vanishes since sin(0)=sin(π)=0. This implies that the neighborhood of the antipodal configuration is locally very flat. Consequently, the gradient descent algorithm produces very small correction steps in this region and may converge extremely slowly or even stall near the stationary point. For the complete cost function (11) which uses both sensors, the same geometric reasoning extends naturally:

(33) $$\frac{\partial J}{\partial \gamma_{a}}=2\sin\gamma_{a},\;\frac{\partial J}{\partial \gamma_{m}}=2\sin\gamma_{m}.$$

If both angular errors are close to π, both partial derivatives are close to zero and the overall gradient is small. It should be noted that, in the full quaternion space $S^{3}$, this point does not necessarily correspond to a strict local extremum, but rather exhibits stationary saddle-like behavior, which is consistent with the quasi-convexity analysis of attitude-estimation cost functions based on vector observations presented in [40]. For this reason, the initial error angle in the static noiseless simulations is set to 175° rather than exactly 180°, thereby ensuring a nonzero initial gradient magnitude and preventing the convergence comparison from being obscured by saddle-point stalling. In practical applications, sensor noise alone is generally sufficient to drive the estimate away from the saddle-point neighborhood, making this issue of limited practical significance.

Under static noiseless conditions, four algorithms are compared: the Madgwick algorithm, the Mahony explicit complementary filter, GDCF-CG with K=0.90, and the proposed GDCF-DG with $K_{0}=1\times 10^{-5}$. For the proposed GDCF-DG, $K_{0}$ is selected according to (28) such that the dynamic gain approaches the GDCF-CG design value K=0.90 after the initial transient. Under noiseless static conditions, the residual cost-function level is governed primarily by the termination criterion rather than by measurement noise. For $\Delta J_{\max}=1\times 10^{-6}$, the cost function settles at approximately $\bar{J}\approx 1\times 10^{-6}$ after the transient. According to (28), this gives $K_{0}=9\bar{J}\approx 9\times 10^{-6}$, for which the rounded value $K_{0}=1\times 10^{-5}$ is used. Once the gradient-descent procedure terminates, the estimate remains stationary in this noiseless static scenario. Consequently, $K_{0}$ primarily affects the transient response rather than the final estimation error, which is consistent with the objective of the proposed approach to improve convergence while preserving steady-state performance.

The convergence behavior of the Madgwick and Mahony algorithms depends on their respective fixed gains, β and $k_{P}$, which control the strength of the attitude correction and thereby influence the trade-off between convergence speed and steady-state behavior. The comparison in this subsection is therefore performed at two tuning levels. At the first level, each algorithm is evaluated using a gain reported by its authors: β=0.041, the value derived for the MARG implementation in [30], and $k_{P}=1$ rad/s, the value used in both experiments reported in [33]. At the second level, both algorithms are assigned substantially higher gains, β=0.50 and $k_{P}=9$, selected to accelerate their transient response while avoiding instability or pronounced steady-state oscillations. Both GDCF variants retain the same parameters at both comparison levels, since the proposed dynamic-gain formulation does not require separate tuning for the acquisition phase.

The simulations were performed assuming a constant object orientation defined by the quaternion

(34) $$q_{\mathrm{true}}={\left[\begin{array}{cc} \cos(\alpha /2) & \sin(\alpha /2)n \end{array}\right]}^{T},$$

where α=45° and $n=\left(1/\sqrt{2}\right){\left[1,\;-1,\;0\right]}^{T}$, yielding $q_{\mathrm{true}}\approx {\left[0.9239,\;0.2706,\;-0.2706,\;0\right]}^{T}$.

Using (5), the corresponding rotation matrix $R_{i}^{b}\left(q\right)$ was computed and used to generate the body-frame representations of the gravitational acceleration and magnetic-field vectors according to

(35) $$a^{b}=R_{i}^{b}a^{i},\;m^{b}=R_{i}^{b}m^{i}.$$

Since the object remains stationary throughout the simulation, the angular velocity is $\omega ={\left[0,\;0,\;0\right]}^{T}$ rad/s.

**Figure 4:**
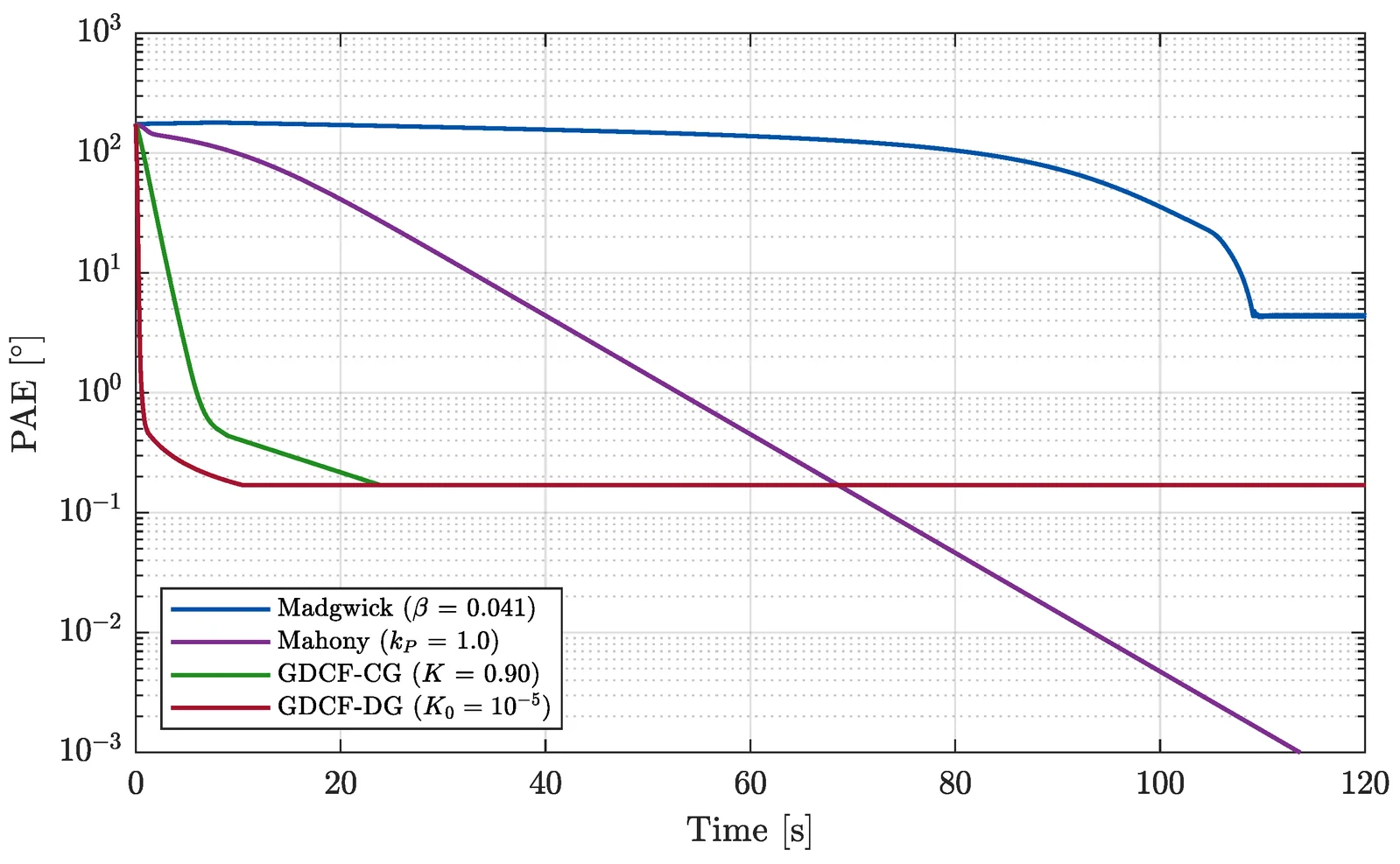
Static noiseless simulation—principal angle error (PAE) with the Madgwick algorithm and the Mahony filter using gains reported by their authors, β=0.041 and $k_{P}=1$ rad/s.

Figure 4 shows the principal angle error over an extended time span, with the Madgwick algorithm and the Mahony filter operating at the gains reported by their authors. Convergence is defined here, and throughout this section, as the first time instant at which the principal angle error falls below 10°. According to this criterion, the Madgwick algorithm requires 107.9 s and the Mahony filter 32.9 s to recover from the 175° initial offset, whereas GDCF-CG requires 3.2 s and the proposed GDCF-DG only 0.3 s. At these gains, neither reference algorithm achieves convergence on a time scale comparable to that of either GDCF variant.

For this reason, the remaining comparisons in this subsection use a more aggressive setting for both reference algorithms. Increasing the gain during initialization is explicitly suggested for the Madgwick algorithm in [38], where a higher value of β is applied during the first 10 s of each experiment to accelerate convergence. In the present simulation, β=0.50 is selected as an aggressive setting that substantially accelerates convergence while keeping the resulting steady-state oscillations well below the 10° convergence threshold.

For the Mahony filter, increasing $k_{P}$ similarly accelerates the attitude-error correction, but excessively large values lead to degraded discrete-time behavior at the sampling frequency considered here. The value $k_{P}=9$ was found to provide the fastest convergence among the tested settings while retaining stable behavior at $f_{s}=10$ Hz. No acquisition-specific gain-scheduling procedure is prescribed in [33]. These tuning strategies are external to the estimators themselves: the acquisition strategy suggested for the Madgwick algorithm requires switching between gain values according to a schedule specified in advance, while the Mahony filter does not provide an analogous acquisition-specific mechanism. In contrast, the proposed GDCF-DG continuously adapts the filter gain according to the current value of *J*, using a single parameter and without requiring a predefined gain schedule.

**Figure 5:**
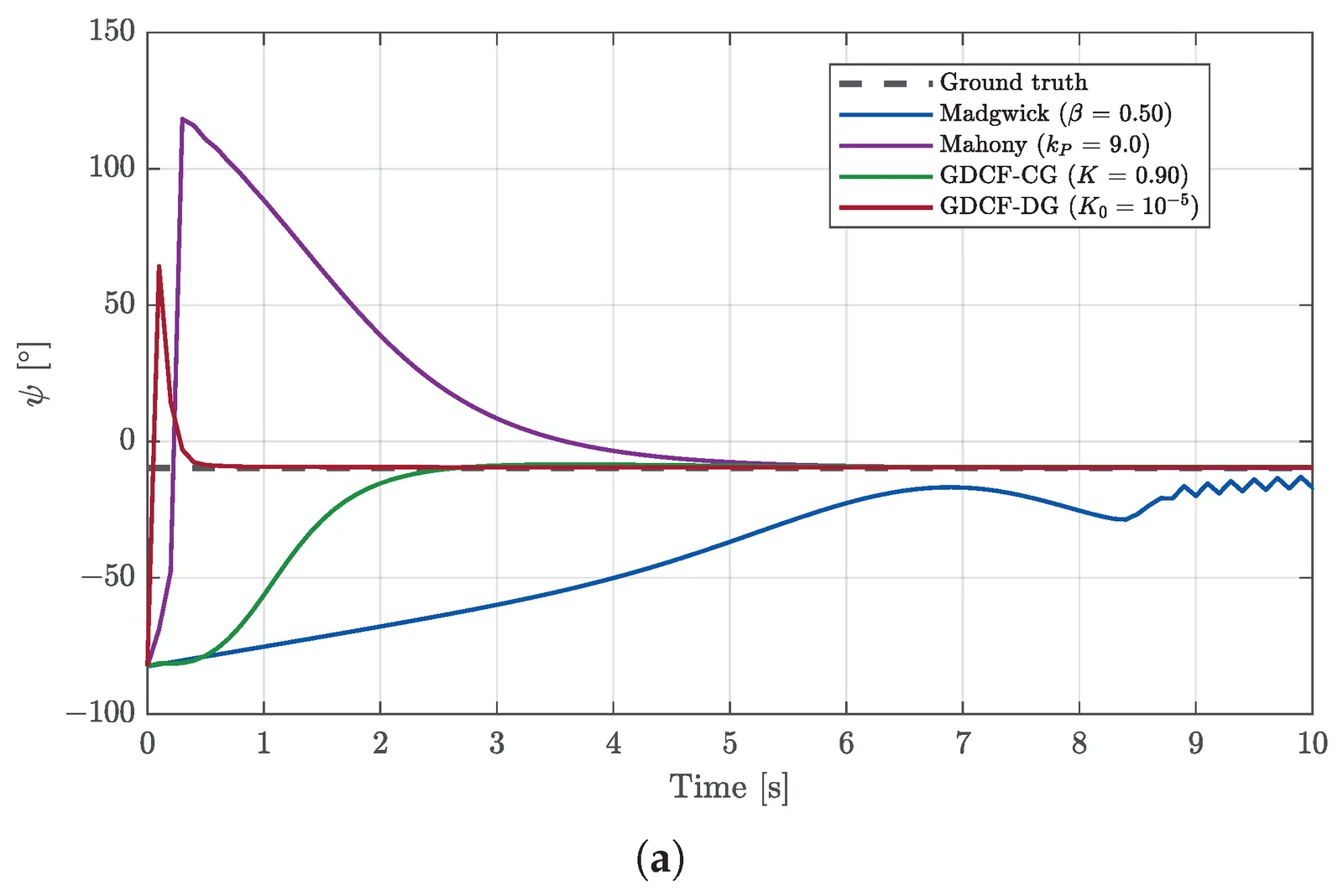

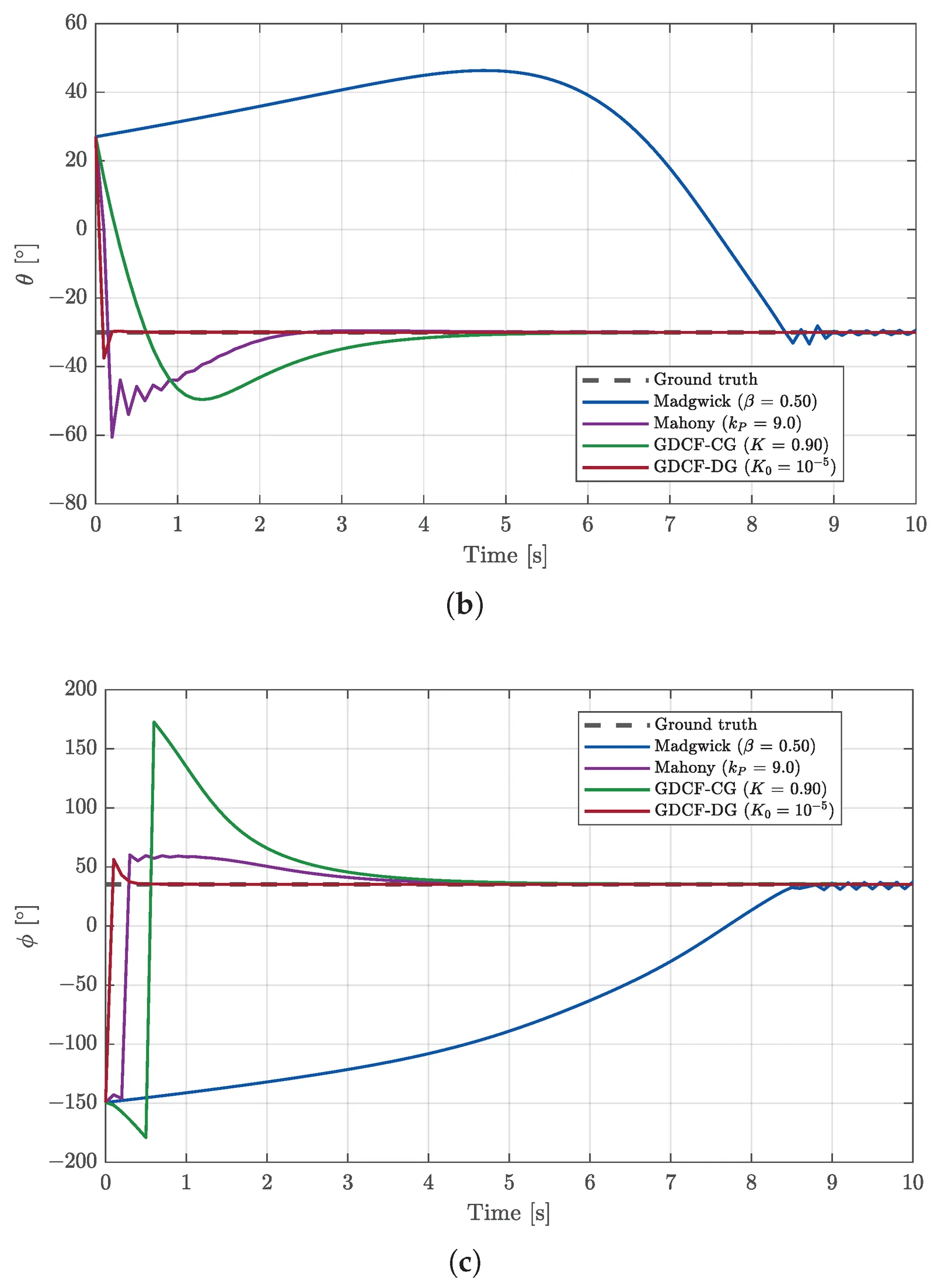
Static noiseless simulation—comparison of true and estimated Euler angles for the Madgwick algorithm, the Mahony algorithm, GDCF-CG and GDCF-DG algorithms. (**a**) Yaw angle ψ. (**b**) Pitch angle θ. (**c**) Roll angle ϕ.

Figure 5 presents the estimated attitude obtained using the Madgwick algorithm, the Mahony filter, GDCF-CG, and GDCF-DG, together with the ground-truth orientation. Although all compared algorithms internally use quaternion form, the estimated orientations are represented in terms of Euler angles using (6) in order to provide a more intuitive visualization of the convergence transient. At t=0, all algorithms share the same initial quaternion corresponding to the 175° offset from the true orientation. The divergence of the trajectories after the first update step illustrates the differences in correction aggressiveness among the compared approaches.

For the GDCF-CG implementation, the complementary filter gain was set to K=0.90, thereby assigning greater weight to the gyroscope-based estimate. The same gain is used in the dynamic noisy scenario of Section 4.2, where sufficient weighting of the gyroscope-based estimate is required to suppress the influence of measurement noise, and is retained here to provide a common steady-state weighting for the comparison. For GDCF-DG, the corresponding value of $K_{0}$ is determined according to (28) for the residual cost-function level of each scenario, while targeting the same steady-state weighting. Under noiseless conditions, a smaller value of *K* would accelerate the convergence of GDCF-CG, but would increase the influence of accelerometer and magnetometer measurement noise under noisy conditions, thereby degrading steady-state performance. The proposed dynamic gain is specifically intended to alleviate this trade-off by providing stronger correction during the convergence transient while preserving the desired steady-state weighting. Figure 5 shows that the proposed GDCF-DG substantially shortens the convergence transient compared with GDCF-CG, the Mahony filter, and the Madgwick algorithm, even when the latter two are configured using the aggressive initialization-oriented settings described above.

**Figure 6:**
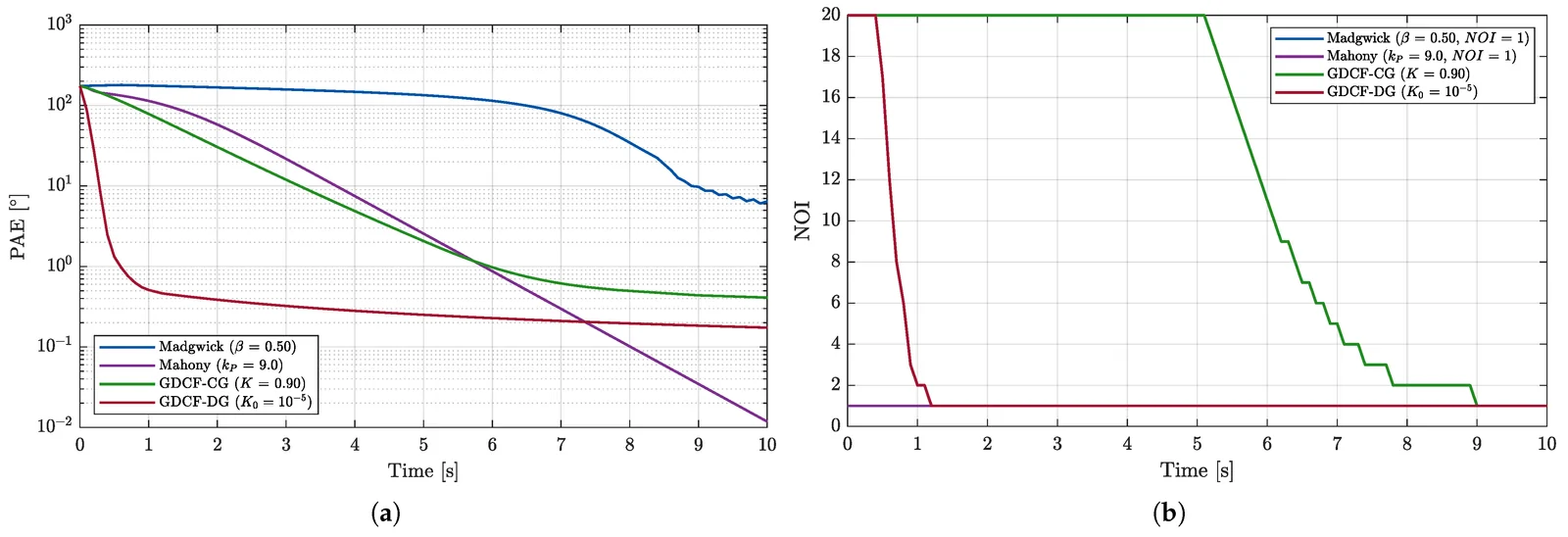
Static noiseless simulation results comparing the Madgwick algorithm, the Mahony filter, GDCF-CG, and GDCF-DG. (**a**) Principal angle error (PAE). Note the narrowed time span compared with Figure 4. (**b**) Number of iterations (NOI).

Figure 6 presents the principal angle error (PAE) and the number of iterations (NOI) for the considered static noiseless scenario.

The convergence advantage of the proposed dynamic-gain formulation is most clearly quantified through the principal angle error shown in Figure 6a. Under the convergence criterion defined above, GDCF-DG with $K_{0}=1\times 10^{-5}$ converges at t=0.3 s, whereas GDCF-CG requires t=3.2 s, corresponding to an approximately tenfold speedup. At their aggressive settings, the Mahony filter requires t=3.8 s and the Madgwick algorithm t=8.9 s. The proposed formulation therefore converges approximately 13 and 30 times faster than the Mahony and Madgwick algorithms, respectively, even when both reference algorithms are configured using the aggressive settings described above.

This acceleration of the transient response does not degrade the steady-state estimation accuracy. Once convergence is reached, both GDCF variants achieve the same steady-state PAE of 0.17°. In contrast, the principal angle error of the Mahony filter continues to decrease throughout the steady-state portion of the simulation. Because the magnitude of its correction decreases with the attitude error, the estimate progressively approaches the true attitude without reaching a measurement-induced error floor. This behavior results from the idealized noiseless conditions rather than from an inherent steady-state advantage of the filter: the measurements correspond exactly to the true attitude and therefore introduce no disturbance that would establish a nonzero residual error level. When sensor noise is present, the achievable steady-state accuracy is instead limited by the measurement disturbances, as demonstrated in Section 4.2.

Figure 6b shows the number of iterations at each time step. For the Madgwick algorithm and the Mahony filter, this quantity is fixed at 1, since both algorithms perform exactly one correction step per sample. For both GDCF-CG and GDCF-DG, the initial NOI is equal to $N_{\max}=20$, reflecting the large initial estimation error and the corresponding high value of the cost function *J*. As the estimates approach the true orientation, the NOI decreases and, once steady state is reached, the termination criterion is satisfied immediately at every sample, so that the inner gradient-descent loop terminates with NOI =0 for both GDCF variants. The advantage of the dynamic-gain formulation in terms of iterative workload therefore manifests primarily during the transient phase: GDCF-DG spends fewer samples in the high-NOI regime than GDCF-CG, which lowers the total number of iterations required to acquire the correct orientation. This property is particularly relevant for embedded scenarios involving frequent re-initialization (cold start, fault recovery, or sleep-to-active transitions), where reducing the computational workload associated with orientation acquisition may be beneficial.

Increasing the gain of the Madgwick algorithm to β=0.50 substantially accelerates convergence, but it does so at the cost of the accuracy retained afterwards: on this record the error left once the transient has decayed is 5.08°, averaged over the final half of the simulation interval. The normalized gradient correction employed in the Madgwick formulation has a magnitude scaled by β and does not decrease proportionally with the magnitude of the attitude error as the estimate approaches the true orientation. Consequently, at this relatively high value of β, the estimate does not settle at the true orientation but oscillates around it.

The reported steady-state PAE should not, however, be attributed entirely to this behavior. Part of the residual error results from a difference in the heading reference against which the estimate is compared. The Madgwick algorithm constructs its reference geomagnetic field from the magnetometer measurement, resulting in a heading reference associated with the local magnetic field. In contrast, the reference attitude used in this simulation is expressed with respect to geographic north, while the magnetic-field vector obtained from the World Magnetic Model includes the local magnetic declination. Consequently, part of the reported steady-state PAE reflects the difference between these heading references rather than the estimation accuracy of the algorithm itself. This does not affect the conclusions regarding convergence time, which is the primary quantity of interest in this comparison, because the resulting residual error remains well below the 10° convergence threshold defined above.

In contrast to the acquisition strategy suggested for the Madgwick algorithm, which switches between two gain values, GDCF-CG exhibits smooth steady-state behavior under noiseless conditions using a single gain setting, but its fixed complementary-filter gain limits the attainable convergence speed from large initial orientation errors.

The proposed GDCF-DG overcomes this limitation by dynamically adjusting the complementary-filter gain according to the current value of the cost function. During the transient phase, the gain adaptation favors the gradient-descent correction, thereby accelerating convergence, while near the equilibrium point the filter behavior gradually transitions toward gyroscope-dominated propagation. Consequently, the proposed formulation achieves substantially faster convergence while preserving the smooth steady-state behavior of the constant-gain formulation using a single parameter configuration.

**Figure 7:**
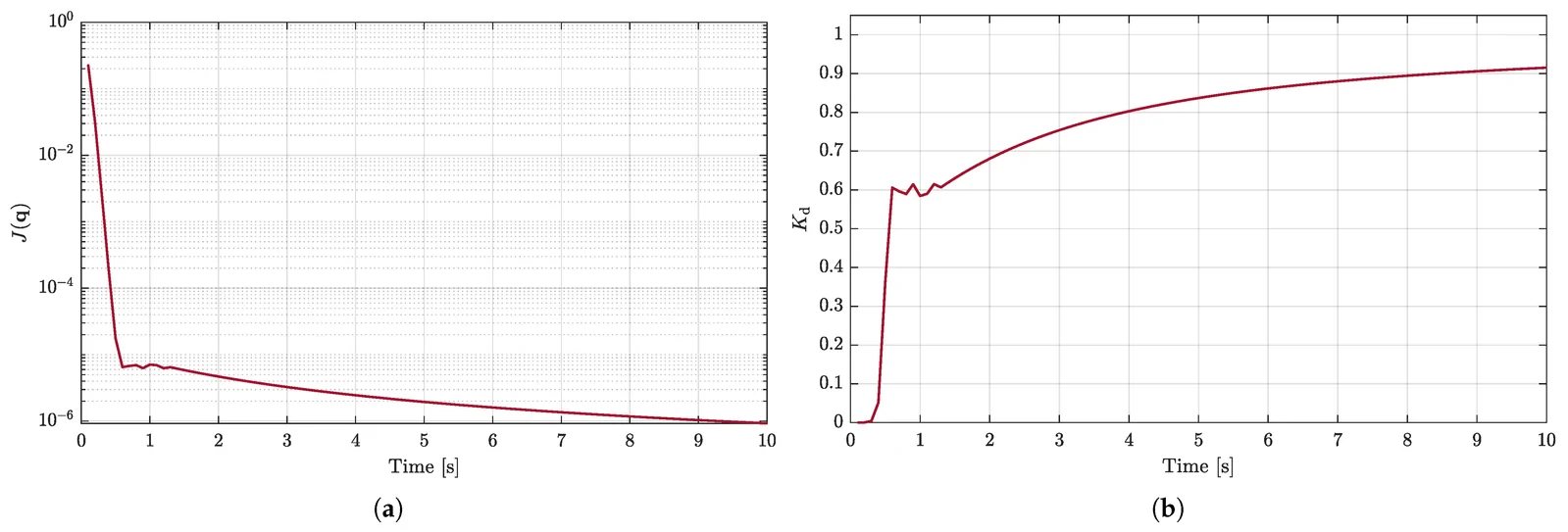
Static noiseless simulation during convergence from a 175° initial orientation offset using $K_{0}=1\times 10^{-5}$. (**a**) Evolution of the cost function *J*. (**b**) Evolution of the complementary-filter gain $K_{d}$ of the GDCF-DG algorithm.

The mechanism responsible for the convergence acceleration achieved by the proposed dynamic-gain formulation is illustrated in Figure 7. Figure 7a shows the evolution of the cost function *J*, while Figure 7b shows the corresponding complementary-filter gain $K_{d}$ during the first 10 s of the simulation. As the cost function decreases, $K_{d}$ progressively increases and approaches the target steady-state value of K=0.90, remaining close to this value after approximately 10 s.

At the beginning of the simulation, the large initial orientation error produces a high value of the cost function, which, according to (26), results in a low value of the complementary filter gain. Consequently, greater weight is assigned to the gradient-descent-based estimate, enabling rapid correction of the initial attitude mismatch.

As the estimated orientation approaches the true solution, the cost function decreases and the complementary filter gain gradually increases, thereby shifting the filter behavior toward gyroscope-dominated propagation. The proposed strategy therefore enables an effective transition from aggressive transient correction to smooth steady-state operation within a single adaptive complementary-filter framework.

### 4.2. Dynamic Noisy Simulations

In this simulation scenario, the object rotates at a constant angular velocity of 20 deg/s about the axis $n={\left[1,\;-1,\;0\right]}^{T}$, in contrast to the static simulations in which the object remained stationary. The initial rotation angle about n is set to $\alpha_{0}=45{}^{\circ}$.

The simulation is conducted as previously described. At each sampling instant (0.1 s), the ground-truth quaternion is computed and used to generate the corresponding synthetic sensor data. The estimated orientations are represented in terms of Euler angles for visualization purposes, while the estimation accuracy is evaluated using the principal angle error (PAE) at every time step. The compared algorithms are the Madgwick algorithm, the Mahony filter, GDCF-CG, and GDCF-DG.

Unlike the static noiseless scenario, where a very small $\Delta J_{\max}$ can be used, in the noisy scenarios a larger value of $\Delta J_{\max}=1\times 10^{-3}$ is adopted. Under measurement noise, the cost function does not reach zero even at the correct orientation, and reducing $\Delta J_{\max}$ below this level only increases the number of iterations without improving accuracy.

The previous subsection demonstrated that introducing a dynamic gain into the complementary filter can substantially reduce the transient time required for the estimator to recover from an incorrect initial quaternion. Here, as in the static simulations, GDCF-CG is configured with K=0.90, thereby assigning greater weight to the gyroscope-based estimate. This choice is consistent with the typical operating principle of complementary filtering for MARG-based attitude estimation, where the gyroscope provides reliable short-term orientation estimation while the gradient-descent correction primarily compensates for drift and accumulated attitude error.

In the proposed GDCF-DG formulation, however, the effective complementary-filter gain varies over time according to (26). Consequently, the parameter $K_{0}$ cannot be directly compared with the fixed gain *K* used in GDCF-CG. Instead, $K_{0}$ is determined according to the procedure introduced in Section 3.3, with the objective of reproducing approximately the same steady-state weighting as the fixed gain K=0.90 used in GDCF-CG. The constant-gain filter is run once over the same dynamic noisy record with correct initialization, so that transient recovery does not affect the determination of the residual level, yielding $\bar{J}=4.3\times 10^{-4}$. The selection rule (28) then gives $K_{0}=3.9\times 10^{-3}$, which is rounded to $K_{0}=0.004$ for use in this simulation. The resulting dynamic gain is subsequently verified over the dynamic noisy record, yielding a time-averaged value of $\bar{K_{d}}\approx 0.90$. Thus, the design procedure provides approximately the same effective complementary weighting as GDCF-CG while allowing the gain to vary dynamically during operation.

**Figure 8:**
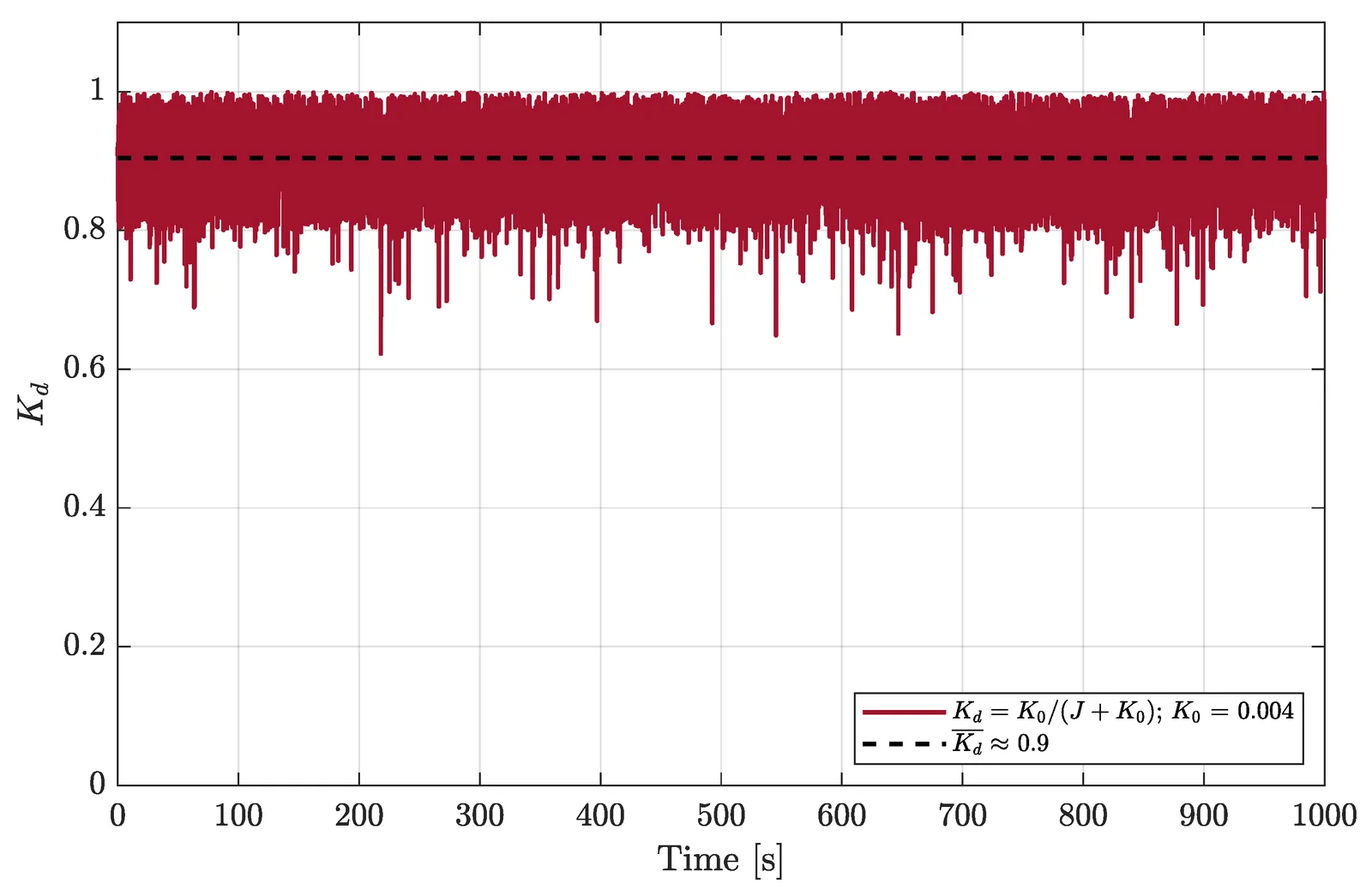
Dynamic noisy simulation—complementary-filter gain $K_{d}$ of the GDCF-DG algorithm as a function of time for $K_{0}=0.004$. The dashed line indicates the time-averaged value $\bar{K_{d}}\approx 0.90$.

The time evolution of the complementary filter gain $K_{d}$ is shown in Figure 8. According to (26), the gain varies over time because the cost function J(t) fluctuates in the presence of rotational motion and measurement noise. For most of the simulation, $K_{d}$ remains within the range from 0.8 to 1.00, indicating that in steady state GDCF-DG relies predominantly on the gyroscope-based quaternion estimation. This behavior is desirable, since the gyroscope provides smooth short-term estimation, while the gradient-descent correction primarily compensates for accumulated attitude error.

The temporary drops of $K_{d}$ reflect the adaptive response of GDCF-DG to increases in the cost function caused by noise and residual estimation mismatch. Despite these fluctuations, the time-averaged value remains $\bar{K_{d}}\approx 0.90$, confirming the intended steady-state weighting obtained using the $K_{0}$ selection procedure. Therefore, even under dynamic noisy conditions, the proposed GDCF-DG preserves the fundamental operating principle of the complementary filter while retaining the ability to temporarily strengthen the gradient-descent correction when required.

The simulations were performed using the following parameter values: β=0.041 for the Madgwick algorithm, $k_{P}=1$ rad/s for the Mahony filter, K=0.90 for GDCF-CG, and $K_{0}=0.004$ for GDCF-DG. For the Madgwick algorithm, β=0.041 corresponds to the value derived for the MARG implementation in [30]. For the Mahony filter, $k_{P}=1$ rad/s corresponds to the value used in the experiments reported in [33]. Thus, both reference algorithms are evaluated using gain values reported in their respective original works rather than values selected specifically for the present comparison. Consistent with the static simulations, the worst-case initial quaternion was used at the moment of filter initialization, corresponding to an initial offset of 175° from the true orientation.

**Figure 9:**
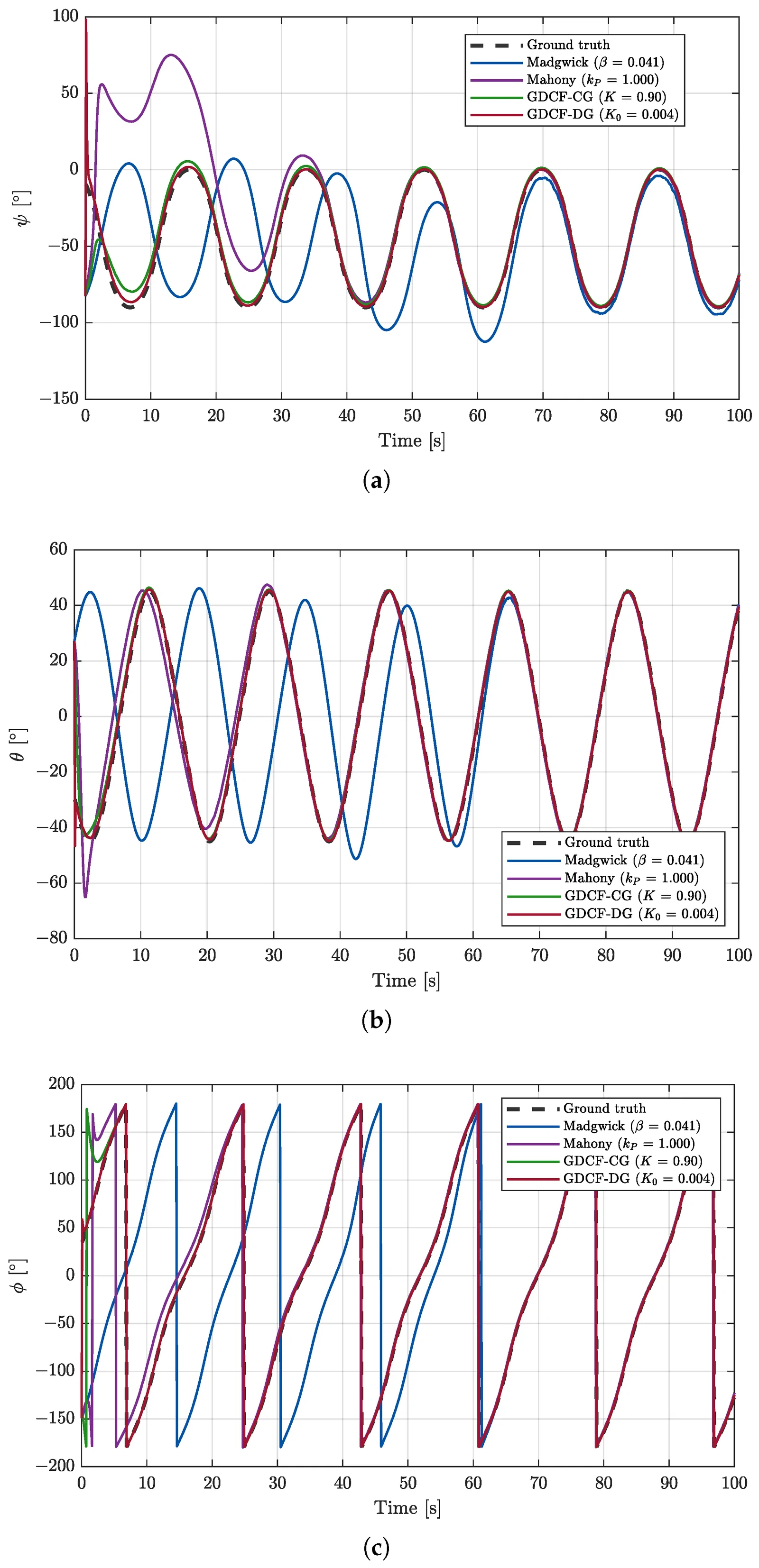
Dynamic noisy simulation—comparison of true and estimated Euler angles for the Madgwick algorithm, the Mahony algorithm, GDCF-CG and GDCF-DG algorithms. (**a**) Yaw angle ψ. (**b**) Pitch angle θ. (**c**) Roll angle ϕ.

Figure 9 presents the estimated attitude obtained using the Madgwick algorithm, the Mahony filter, GDCF-CG, and GDCF-DG, together with the ground-truth orientation. As in the static simulations, the estimated orientations are represented in terms of Euler angles using (6) in order to provide an intuitive visualization of the convergence transient and the subsequent steady-state tracking behavior. At t=0, all algorithms are initialized with the same quaternion corresponding to the 175° offset from the true orientation, while differences between the trajectories emerge after the first filter update step.

**Figure 10:**
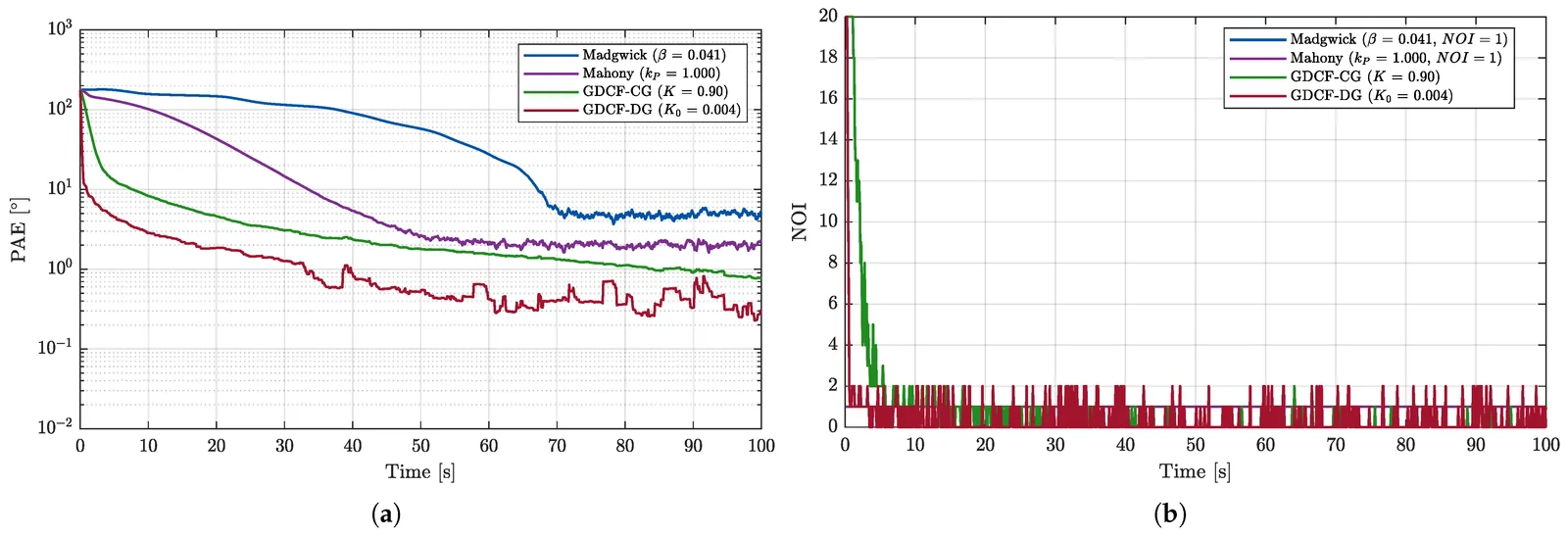
Dynamic noisy simulation results comparing the Madgwick algorithm, the Mahony filter, GDCF-CG, and GDCF-DG. (**a**) Principal angle error (PAE). (**b**) Number of iterations (NOI).

From the Euler-angle trajectories, it is apparent that, following the large initial orientation error, GDCF-DG converges most rapidly toward the true orientation, while GDCF-CG exhibits a somewhat slower transient response. In contrast, the Madgwick algorithm and the Mahony filter exhibit slower convergence under the same initialization conditions. A quantitative comparison of the algorithm performance is provided by the PAE and NOI plots shown in Figure 10.

The behavior observed in the Euler-angle trajectories is quantified more clearly through the PAE results shown in Figure 10a. Using the same convergence criterion as in the static case, i.e., the time required for the PAE to fall below 10°, the Madgwick algorithm converges at t=67.2 s and the Mahony filter at t=33.6 s, whereas GDCF-CG requires t=7.7 s and GDCF-DG only t=1.0 s. Thus, in the considered dynamic noisy scenario, the proposed dynamic-gain formulation reduces the convergence time by approximately 8× relative to GDCF-CG, 34× relative to the Mahony filter, and 67× relative to the Madgwick algorithm.

Over the full simulation interval (excluding t=0), the average PAE is 11.40° for the Madgwick algorithm, 4.31° for the Mahony filter, compared with 0.71° for GDCF-CG, and 0.42° for GDCF-DG. In steady state, evaluated over the final 50% of the simulation interval, the average PAE is 4.84° for the Madgwick algorithm, 2.01° for the Mahony filter, 0.22° for GDCF-CG, and 0.31° for GDCF-DG. These results indicate that the proposed GDCF-DG achieves the fastest transient convergence while preserving steady-state accuracy comparable to that of GDCF-CG. Although GDCF-CG achieves a slightly lower steady-state PAE in the considered simulation, both formulations exhibit comparable steady-state accuracy, while GDCF-DG provides a substantial improvement in convergence speed.

It should be noted that the residual steady-state PAE values are also influenced by algorithmic and discrete-time characteristics of the compared estimators. In particular, the residual error of the Mahony filter may be influenced by its discrete-time implementation at the considered sampling rate, while for the Madgwick algorithm the magnetic-reference convention additionally contributes to the reported PAE, as discussed previously. Therefore, the absolute PAE values reported here should not be interpreted as intrinsic steady-state accuracy limits of the respective algorithms. Since the present work primarily focuses on transient convergence, no algorithm-specific modifications were introduced to compensate for these effects.

Additional insight into the computational behavior of the compared algorithms is provided by the NOI results shown in Figure 10b. For the Madgwick algorithm and the Mahony filter, the NOI is fixed at 1, since both algorithms perform exactly one correction step per sample. In contrast, both GDCF-CG and GDCF-DG initially reach the maximum number of iterations ($N_{\max}=20$) immediately after initialization due to the large initial orientation error. However, once the transient recovery phase is completed, the required NOI rapidly decreases to very low values.

Consequently, despite their iterative formulation, the average NOI values of GDCF-CG and GDCF-DG over the entire simulation are only 0.28 and 0.26, respectively, both corresponding to less than one third of the fixed NOI of the two reference algorithms. During steady-state operation, evaluated over the final 50% of the simulation interval, the average NOI decreases further to 0.22 and 0.24, respectively. These results demonstrate that the adaptive termination criterion substantially limits the iterative gradient-descent workload during normal filter operation.

Although GDCF-DG exhibits substantially faster transient convergence than GDCF-CG, both algorithms retain nearly identical average NOI values. Thus, the substantial improvement in convergence speed is achieved without increasing the average iterative gradient-descent workload. As shown by the operation-count analysis in Section 3.4, the dynamic-gain calculation itself introduces only one additional addition and one division per sample relative to GDCF-CG, while the overall arithmetic workload is determined primarily by the respective NOI values.

### 4.3. Convergence–Accuracy Trade-Off

For the configurations considered in this study, the convergence–accuracy trade-off of each algorithm is primarily controlled by its respective gain parameter: β for the Madgwick algorithm, $k_{P}$ for the Mahony filter, *K* for GDCF-CG, and $K_{0}$ for GDCF-DG. Consequently, a comparison at a single operating point depends on the selected parameter values. In this subsection, each parameter is therefore swept over a broad range of values to characterize the resulting trade-off between convergence speed and steady-state accuracy.

Each parameter is swept over 61 logarithmically spaced values using the dynamic noisy record. For each parameter value, the steady-state PAE is evaluated over the final 50% of a simulation initialized at the correct orientation, while the convergence time is evaluated separately from the 175° initial offset. Through (28), sweeping $K_{0}$ corresponds to varying the design gain of the proposed filter. For each algorithm, only parameter values for which the filter converges within the simulation interval and subsequently remains below the convergence threshold are considered. Among these, Pareto-dominated operating points, for which another value of the same parameter is no worse in either metric and strictly better in at least one, are excluded from the plotted curve. The resulting parameter ranges are $\beta \in [3.1\times 10^{-3},\;0.74]$, $k_{P}\in \left[3.7\times 10^{-2},\;10.0\right]$ rad/s, K∈[0.17,0.99], and $K_{0}\in \left[2.1\times 10^{-3},\;9.4\times 10^{-2}\right]$.

**Figure 11:**
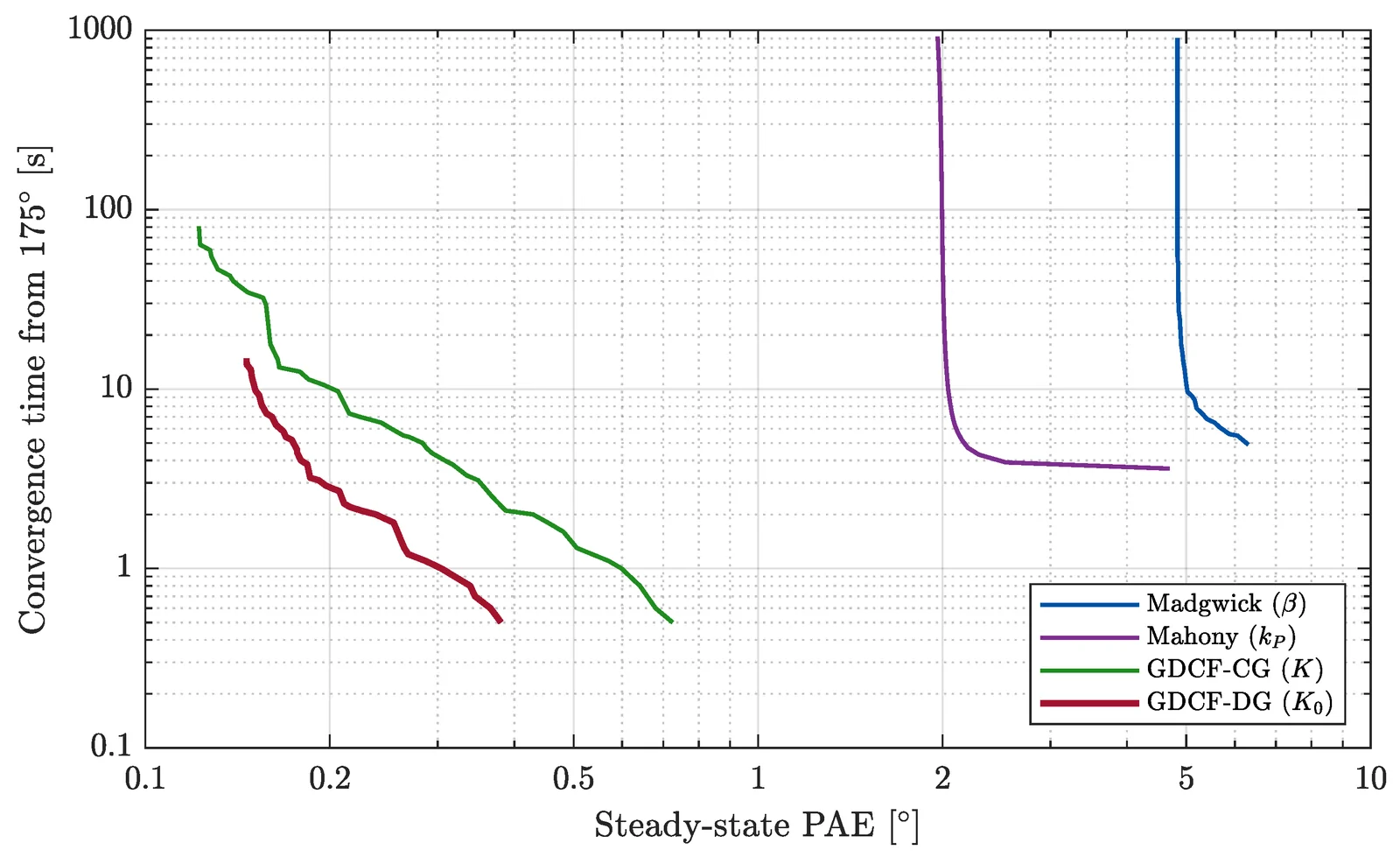
Dynamic noisy simulation—convergence time from the 175° initial offset against steady-state PAE for the Madgwick algorithm, the Mahony filter, GDCF-CG, and GDCF-DG, obtained by sweeping β, $k_{P}$, *K* and $K_{0}$, respectively.

The resulting curves are shown in Figure 11. Over the common range of steady-state PAE, the curve of GDCF-DG lies below that of GDCF-CG; at 0.30°, for example, GDCF-DG converges in 1.0 s and GDCF-CG in 4.2 s. The dynamic gain therefore improves the convergence–accuracy trade-off itself, rather than merely providing a different operating point through parameter tuning. Only at the high-accuracy end does GDCF-CG attain a marginally lower steady-state PAE, at the cost of a considerably longer convergence time.

The curves of the reference algorithms are nearly vertical over most of their range, so that lowering the gain increases the convergence time without reducing the steady-state PAE. Their shortest convergence times over the entire parameter ranges considered are 3.6 s for the Mahony filter and 4.9 s for the Madgwick algorithm, both longer than the 1.0 s achieved by GDCF-DG as described in Section 4.2. The faster convergence of GDCF-DG therefore cannot be attributed solely to the particular gain values selected for the reference algorithms in the preceding comparison.

### 4.4. Monte Carlo Robustness Analysis

The results of Section 4.2 are obtained for a single initial quaternion and a single realization of the measurement noise. To assess the robustness of these results with respect to both factors, the dynamic noisy simulation is repeated over 1000 independent Monte Carlo realizations. In each realization, a new noise sequence is generated using the noise variances specified above, and a new initial quaternion corresponding to a uniformly distributed orientation on SO(3) is generated. Quaternions whose PAE relative to the true initial orientation is below 30° are rejected, ensuring that each realization starts from a substantially incorrect orientation. The resulting initial PAE ranges from 30.3° to 179.8°, with a mean of 128.3°.

The trajectory, sampling interval, estimator parameters (β=0.041, $k_{P}=1$ rad/s, K=0.90, and $K_{0}=0.004$), and gradient-descent parameters are identical to those used in Section 4.2, with no parameter readjustment between realizations. Within each realization, all four algorithms receive identical sensor measurements and the same initial quaternion, providing a paired comparison. Convergence time is defined as the first instant at which the PAE falls below 10°, while the steady-state PAE is averaged over the final 50% of the simulation interval.

As summarized in Table 3, all four algorithms converge in every realization. GDCF-DG exhibits the shortest mean convergence time and the smallest standard deviation.

The steady-state PAE values are also reported to assess their sensitivity to the measurement-noise realization and initial orientation. The steady-state PAE exhibits little variation between realizations, with standard deviations below 0.04°, and is consistent with the results of Section 4.2.

Overall, the Monte Carlo analysis confirms that the convergence advantage of GDCF-DG is robust to variations in both the initial orientation and the measurement-noise realization.

## 5. Experimental Results

**Figure 12:**
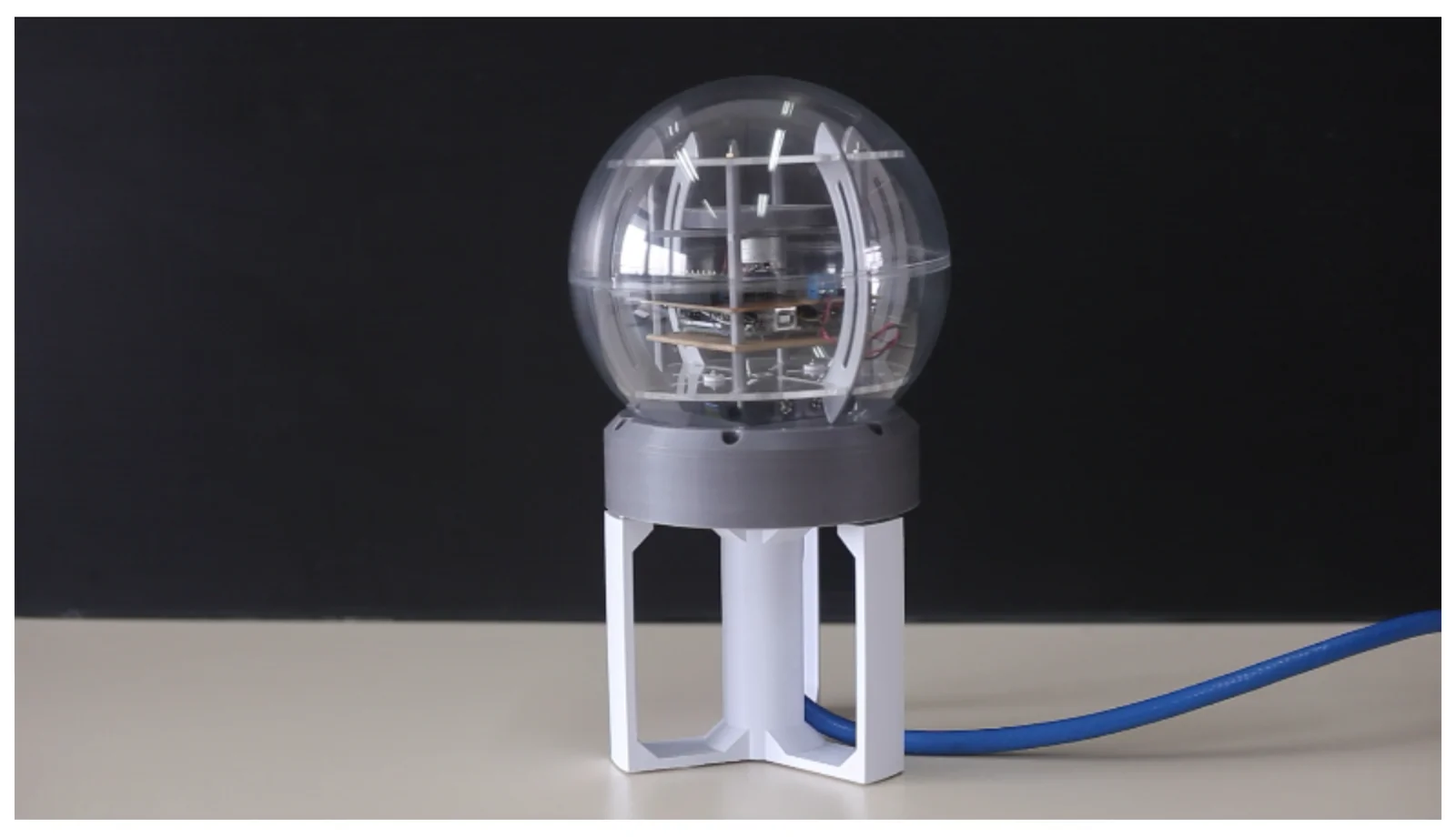
Experimental setup—photograph of the ADCS model used for experimental validation of the proposed gradient-descent-based complementary filter with dynamic gain for attitude estimation.

The proposed GDCF-DG was experimentally validated using a laboratory model of a satellite attitude determination and control system (ADCS), shown in Figure 12. The experimental platform was developed for educational purposes at the University of Zagreb, Faculty of Electrical Engineering and Computing, Department of Communication and Space Technologies.

The ADCS model consists of a microcontroller-based embedded system equipped with an MPU9250 MARG sensor. A block diagram of the experimental platform is provided in the Supplementary Materials accompanying this paper. The sensor measurements were sampled by the microcontroller at a frequency of 10 Hz and wirelessly transmitted to a computer via a Bluetooth module, where they were subsequently processed using the implemented attitude estimation algorithms. The DC motor and its driver shown in the block diagram were part of the ADCS model but were not activated during the experiment. Nevertheless, because the DC motor contains permanent magnets, its presence introduces additional distortions into the local magnetic field, making proper magnetometer calibration particularly important for this experimental setup. Prior to the experiments, the accelerometer, gyroscope, and magnetometer were calibrated, with the complete calibration procedures described in detail in the Supplementary Materials accompanying this paper.

The proposed GDCF-DG algorithm was not implemented directly on the microcontroller; instead, all algorithms were executed on the computer using the same recorded measurement sequence, thereby enabling a direct comparison under identical input conditions. During the experiments, the platform was manually rotated by approximately ±45° about three mutually orthogonal axes using the following rotation sequence: rotation about the *z*-axis, followed by rotation about the *y*-axis, and finally rotation about the *x*-axis.

Two experimental initialization scenarios were considered. In the first scenario, the initial quaternion approximately corresponded to the true initial orientation of the platform and was initialized as $q_{0}={\left[1,\;0,\;0,\;0\right]}^{T}$. In the second scenario, the initial quaternion approximately represented the opposite orientation obtained by a 180° rotation about the *z*-axis, corresponding to $q_{0}={\left[0,\;0,\;0,\;1\right]}^{T}$. These two scenarios were selected in order to evaluate both steady-state tracking performance and transient recovery from large initial orientation errors.

The experimental results presented hereafter were obtained using the parameter values μ=0.07, $N_{\max}=20$, $\Delta J_{\max}=4.16\times 10^{-2}$, $K_{0}=0.40$, K=0.90, β=0.041, $k_{I}=0.3$, and $k_{P}=1$ rad/s. The value β=0.041 corresponds to the value derived for the MARG implementation in [30], while $k_{I}=0.3$ and $k_{P}=1$ rad/s correspond to the values used in the experimental evaluation reported in [33]. Unlike in the simulations, where $k_{I}$ was set to zero because the synthetic gyroscope measurements contained no bias, the integral correction is enabled in the experimental evaluation to account for gyroscope bias in the real sensor measurements.

The value of $K_{0}$ was not obtained by tuning. Following the procedure described at the end of Section 3.3, the constant-gain filter (17) was run once over the recorded data with the design gain K=0.90, and the residual level was determined as the average value of J(q) after the initial transient, yielding $\bar{J}=4.4\times 10^{-2}$. The selection rule (28) then gives $K_{0}=\frac{K}{1-K}\bar{J}=0.396$, for which $K_{0}=0.40$ is adopted. The resulting dynamic gain was subsequently verified over the recorded data, yielding an average value of $\bar{K_{d}}\approx 0.90$. No reference attitude is required for this procedure, since J(q) is evaluated directly from the sensor measurements.

Unlike the simulations, where the synthetic sensor data was generated using norm-preserving reference vectors corrupted only by additive Gaussian noise, the experimental measurements additionally contain magnetic-field distortions, non-gravitational accelerations caused by platform rotation, calibration residuals, and other non-ideal effects inherent to real MARG measurements. Detailed information on the calibration of the sensors used in the experiments, along with raw sensor data demonstrating the presence of the aforementioned acceleration and magnetic disturbances, is provided in the Supplementary Materials accompanying this paper. These non-ideal effects contribute to a substantially higher residual cost-function level than that observed in the dynamic noisy simulation, as anticipated at the end of Section 3.3.

The measured residual level of the experimental setup, $\bar{J}=4.4\times 10^{-2}$, exceeds that obtained in the dynamic noisy simulation using the same procedure, $\bar{J}=4.3\times 10^{-4}$, by a factor of approximately 100. Since (28) makes $K_{0}$ proportional to $\bar{J}$ for a fixed design gain *K*, it consequently yields $K_{0}=0.40$ for the experiment and $K_{0}=0.004$ for the dynamic noisy simulation. Thus, $K_{0}$ is not independently tuned between the two scenarios; in both cases, it is determined using the same design gain K=0.90 and the residual level of the corresponding measurement setup. The two operating points correspond to the intersections of the design value K=0.90 with the respective curves in Figure 2, and the mean gains $\bar{K_{d}}$ measured over both records agree with the approximation in (27) to within 0.01.

**Figure 13:**
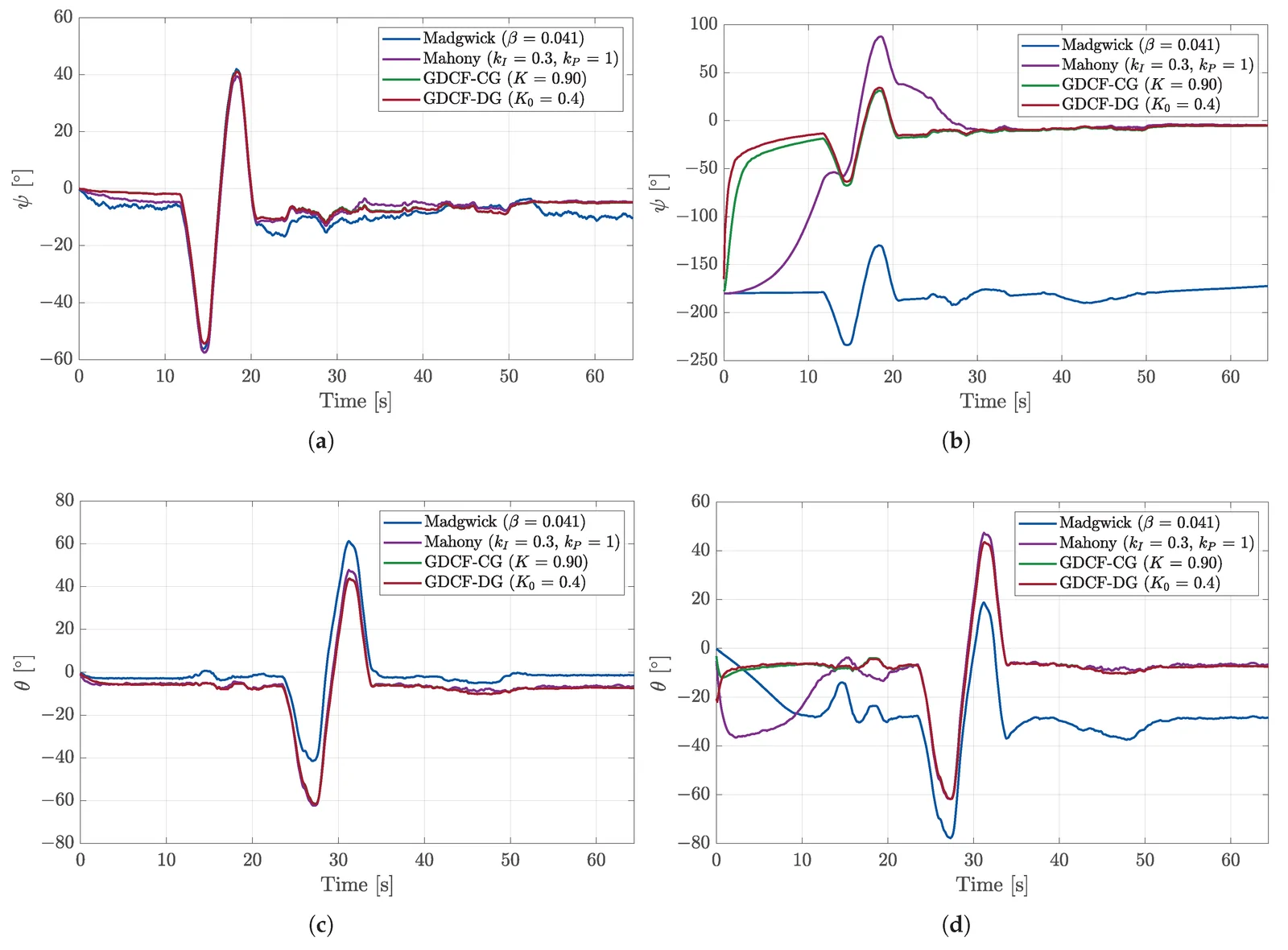

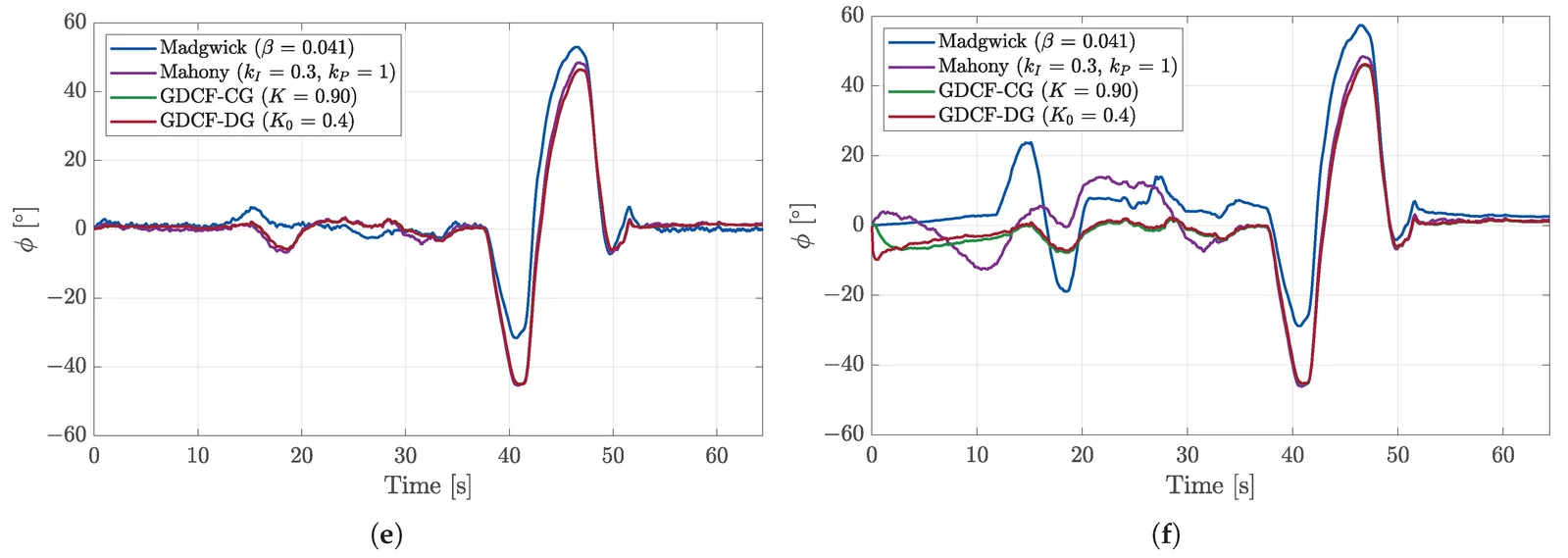
Experimental attitude estimation results for the considered initialization scenarios. The left column corresponds to the correctly initialized quaternion $q_{0}={\left[1,\;0,\;0,\;0\right]}^{T}$, while the right column corresponds to the worst-case initialization quaternion $q_{0}={\left[0,\;0,\;0,\;1\right]}^{T}$. (**a**,**b**) Yaw angle ψ. (**c**,**d**) Pitch angle θ. (**e**,**f**) Roll angle ϕ.

Figure 13 presents the experimentally obtained Euler-angle estimates for the considered initialization scenarios. In the first scenario, where the initial quaternion approximately corresponds to the true initial orientation of the platform, all four algorithms exhibit stable attitude tracking and follow the same motion, although their estimates do not coincide exactly. The observed offsets can be attributed in part to differences in the magnetic-reference formulations used by the algorithms.

The Madgwick algorithm determines its magnetic reference from the measured magnetic field, whereas GDCF-CG, GDCF-DG, and the Mahony filter use the fixed inertial-frame reference vector $m^{i}$. Consequently, changes or distortions in the locally measured magnetic field affect the two formulations differently. In the proposed formulation, inconsistency between the measured magnetic field and the fixed reference vector contributes directly to the cost function J(q). Thus, magnetic-field disturbances remain reflected in the residual cost-function level rather than being incorporated into an updated magnetic reference. This behavior is consistent with the elevated experimental residual level discussed above, although magnetic disturbances are not its only contributing factor.

More pronounced differences between the compared algorithms become visible in the second scenario corresponding to the worst-case initialization quaternion. In this case, both GDCF-CG and GDCF-DG undergo a transient recovery phase while converging toward the true platform orientation. Owing to the dynamic complementary filter gain, GDCF-DG adapts more aggressively during the transient interval and therefore converges faster than GDCF-CG. In contrast, the Madgwick and Mahony algorithms exhibit slower transient responses under the same initialization conditions, with the Madgwick algorithm exhibiting the slowest convergence.

After the transient recovery phase, the estimated Euler angles produced by GDCF-CG and GDCF-DG exhibit comparable behavior, indicating that the proposed dynamic-gain formulation primarily affects the transient convergence behavior while preserving the steady-state tracking characteristics of the complementary filter. These experimental observations further support the conclusions obtained in the simulation study presented in Section 4.

**Figure 14:**
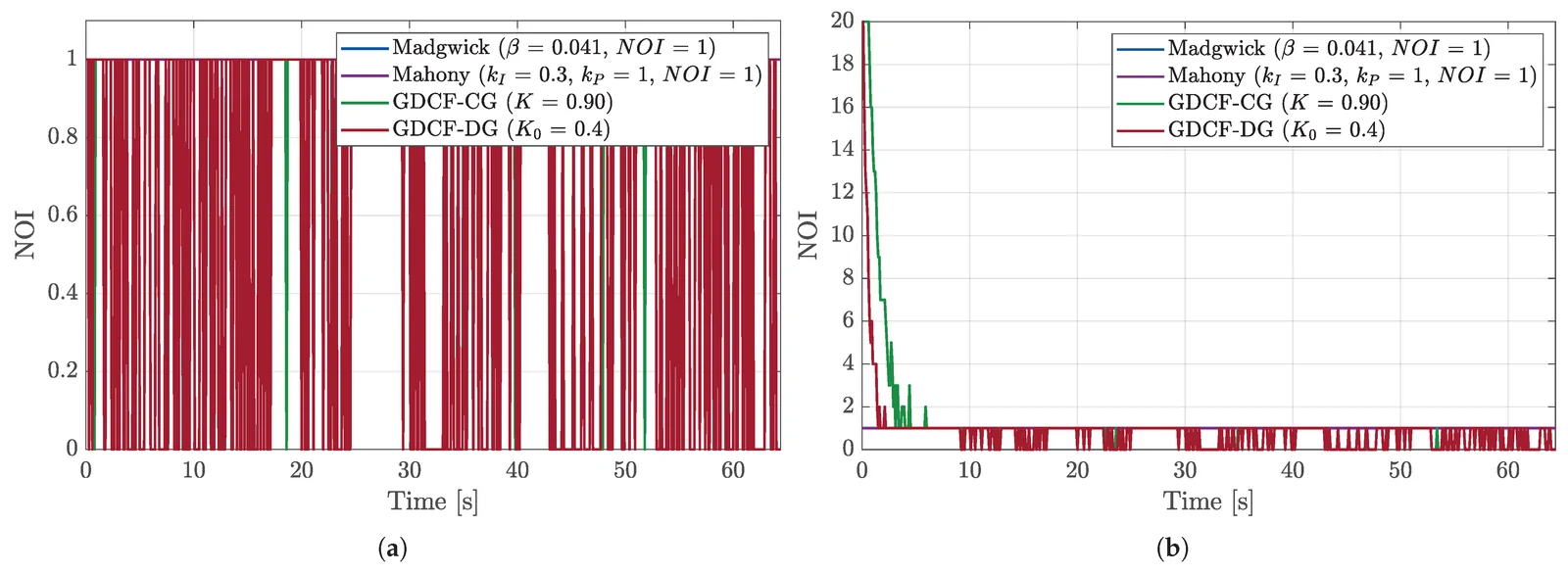
Number of iterations (NOI) required for convergence in the experimental scenarios. (**a**) Correct initialization using $q_{0}={\left[1,\;0,\;0,\;0\right]}^{T}$. (**b**) Worst-case initialization using $q_{0}={\left[0,\;0,\;0,\;1\right]}^{T}$.

Additional insight into the computational behavior of the compared algorithms is provided by the NOI results shown in Figure 14. In the correctly initialized scenario shown in Figure 14a, both GDCF-CG and GDCF-DG exhibit low NOI values throughout the experiment, indicating that only a small number of gradient-descent iterations is required when the initial orientation estimate is close to the true platform attitude.

More pronounced differences become visible in the worst-case initialization scenario shown in Figure 14b. Immediately after initialization, both GDCF-CG and GDCF-DG require a larger number of iterations due to the large initial orientation mismatch. However, because GDCF-DG converges more rapidly during the transient recovery phase, the corresponding high-NOI interval is substantially shorter than in GDCF-CG.

This behavior is also reflected in the average NOI values calculated for the GDCF algorithms. In the correctly initialized scenario, GDCF-CG and GDCF-DG achieve practically the same average NOI, 0.569 and 0.578, respectively. In the worst-case initialization scenario, the average NOI increases to 1.182 for GDCF-CG and 0.856 for GDCF-DG. For comparison, the Madgwick algorithm and the Mahony filter each perform one correction step per sample, represented by the fixed NOI of one in Figure 14; however, these correction steps are not computationally equivalent to a single GDCF gradient-descent iteration.

Using the operation counts derived in Section 3.4, the experimentally observed average NOI values show that the arithmetic workload of GDCF-DG remains comparable to that of the reference algorithms under correct initialization. Moreover, in the worst-case initialization scenario, the reduction in average NOI from 1.182 for GDCF-CG to 0.856 for GDCF-DG compensates for the small additional cost of the dynamic-gain calculation. Consequently, GDCF-DG achieves both faster transient convergence and a lower average arithmetic workload than GDCF-CG in this scenario. These experimental results support the simulation findings and demonstrate that the proposed dynamic-gain formulation improves transient convergence without requiring an increase in the average computational workload.

**Figure 15:**
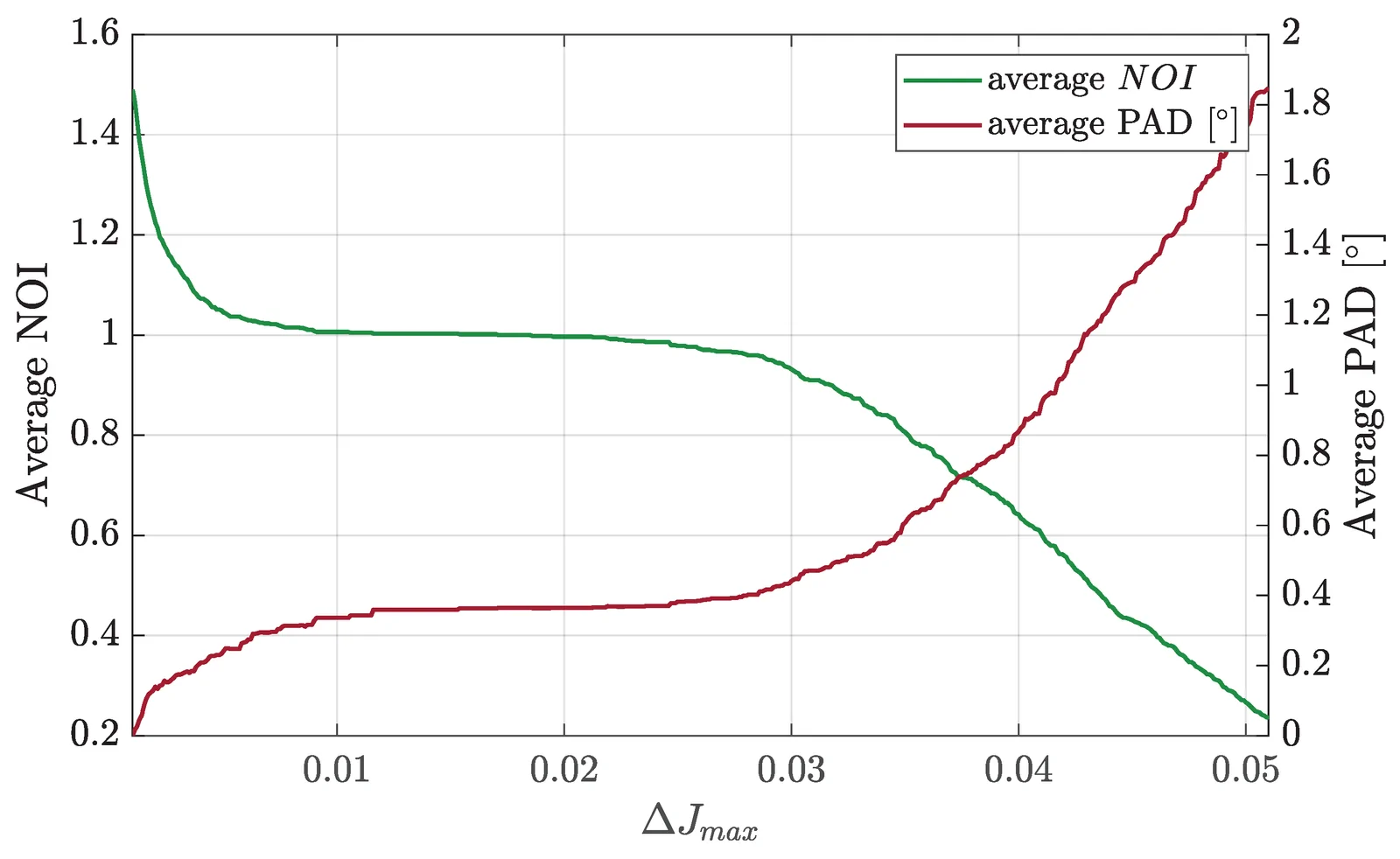
Average principal angle deviation (PAD) and average number of iterations (NOI) as a function of $\Delta J_{\max}$.

The influence of the termination criterion $\Delta J_{\max}$ on the average number of iterations (NOI) and the average principal angle deviation (PAD) is shown in Figure 15. Since no ground-truth orientation is available in the experimental setup, PAD is evaluated by comparing two attitude estimations obtained using different values of $\Delta J_{\max}$. The reference estimation corresponds to the conservative value $\Delta J_{\max}=1\times 10^{-3}$ adopted in the dynamic noisy simulation scenario.

The results demonstrate a clear trade-off between the iterative gradient-descent workload and agreement with the reference estimate. For very small values of $\Delta J_{\max}$, the average NOI decreases rapidly while the average PAD remains low. In the interval approximately between $\Delta J_{\max}=0.01$ and $\Delta J_{\max}=0.03$, both quantities remain nearly constant, indicating that the termination criterion can be significantly relaxed without substantially affecting the attitude estimation performance. Beyond approximately $\Delta J_{\max}=0.03$, the average NOI decreases rapidly, but this reduction is accompanied by a pronounced increase in the average PAD. This region therefore represents the practical trade-off boundary between the iterative gradient-descent workload and agreement with the reference estimate, forming a characteristic knee point in the trade-off curve. Consequently, smaller values of $\Delta J_{\max}$ provide higher agreement with the reference estimate at the expense of an increased number of gradient-descent iterations, whereas larger values substantially reduce the iterative workload at the cost of additional deviation from the reference estimate.

Based on these results, the value $\Delta J_{\max}=4.16\times 10^{-2}$ was selected for the experimental evaluation. At this operating point, the average PAD remains below 1° relative to the reference estimate, while the average NOI is reduced to approximately 0.58. Thus, the selected termination criterion substantially limits the average iterative gradient-descent workload while maintaining sub-degree agreement with the reference estimate. In conjunction with the operation-count analysis presented in Section 3.4, these results demonstrate the computational benefit of the adaptive termination criterion without relying on NOI alone as a measure of computational burden.

## 6. Conclusions

This paper presented a dynamic-gain extension of a gradient-descent-based complementary filter for attitude estimation. The proposed GDCF-DG uses the cost function already available within the gradient-descent procedure to adapt the complementary-filter gain, thereby avoiding the need for an additional error metric or computationally demanding optimization procedure. The gain parameter $K_{0}$ is determined from the conventional complementary-filter gain *K* and the residual cost-function level $\bar{J}$ measured from the data, rather than being selected by trial and error. Simulation, Monte Carlo, and experimental results demonstrated substantially faster convergence from incorrect initial conditions than GDCF-CG, the Madgwick algorithm, and the Mahony filter, while maintaining comparable steady-state performance. The operation-count analysis, together with the observed average NOI values, showed that this improvement is achieved without a substantial increase in the average arithmetic workload. The presented results should nevertheless be interpreted in light of certain limitations, including the dependence of the residual level $\bar{J}$, and hence of $K_{0}$, on the selected termination criterion $\Delta J_{\max}$, and the absence of an external ground-truth attitude reference during experimental validation. Future work will focus on experimental validation using an external attitude reference system and on further investigation of lightweight adaptive gain mechanisms, with particular emphasis on computationally constrained attitude estimation systems.

## Figures and Tables

**Table 1:** Number of arithmetic operations per step of the proposed algorithm, listed in the order of execution within one sample.

Step	Executed	×	+/−	÷	x
Gyroscope integration $q_{\omega }$ (incl. normalization)	1	32	19	1	1
Normalization of $a^{b}$ and $m^{b}$	1	12	4	2	2
Cost function J(q), (11)	NOI+1	46	38	0	0
Gradient ∇J(q), (14)	NOI	144	101	0	0
Gradient-descent step, (7)	NOI	4	4	0	0
Quaternion normalization, (8)	NOI	8	3	1	1
Dynamic gain $K_{d}$, (26)	1	0	1	1	0
Complementary fusion (incl. normalization), (17)	1	16	8	1	1

**Table 2:** Number of arithmetic operations per sample. The Mahony and Madgwick algorithms are counted without gyroscope-bias compensation ($k_{I}=0$, ζ=0), as used in Section 4.

Algorithm	×	+/−	÷	x
Mahony	105	62	3	3
Madgwick	160	98	4	5
GDCF-CG	106+202NOI	69+146NOI	4+NOI	4+NOI
GDCF-DG	106+202NOI	70+146NOI	5+NOI	4+NOI

**Table 3:** Monte Carlo results for the dynamic noisy scenario (1000 realizations, mean ± standard deviation).

Algorithm	Convergence Time [s]	Steady-State PAE [°]
Madgwick	56.7±39.3	4.83±0.03
Mahony	23.0±12.2	2.01±0.01
GDCF-CG	7.8±3.9	0.22±0.02
GDCF-DG	1.2±0.6	0.31±0.02

## Data Availability

The original contributions presented in this study are included in the article. Further inquiries can be directed to the corresponding author.

## Supplementary Materials

The following supporting information can be downloaded at: https://www.mdpi.com/article/10.3390/s26206475/s1.
