# Supplementary material for: A Dynamic-Gain Gradient-Descent-Based Complementary Filter for Attitude Estimation
Source: Sensors (Basel). 2026 Oct 13;26(20):6475. doi: 10.3390/s26206475 (PMC13709011; doi:10.3390/s26206475)
Supplement: Supplementary file 1 [file sensors-26-06475-s001.zip › sensors-4412933-supplementary.pdf]

A Dynamic-Gain Gradient-Descent-Based Complementary Filter for Attitude Estimation

Supplemental Material

Andre Poščić, Darko Erceg, Josip Vuković and Josip Lončar*
University of Zagreb Faculty of Electrical Engineering and Computing
Department of Communication and Space Technologies
Unska 3, 10000 Zagreb, Croatia
*E-mail: josip.loncar@fer.hr

1 Experimental Setup

The proposed attitude estimation algorithm is experimentally validated using a laboratory model of a satellite attitude determination and control system (ADCS), shown in Fig. S1. The experimental platform was developed for educational purposes at the University of Zagreb, Faculty of Electrical Engineering and Computing, Department of Communication and Space Technologies.

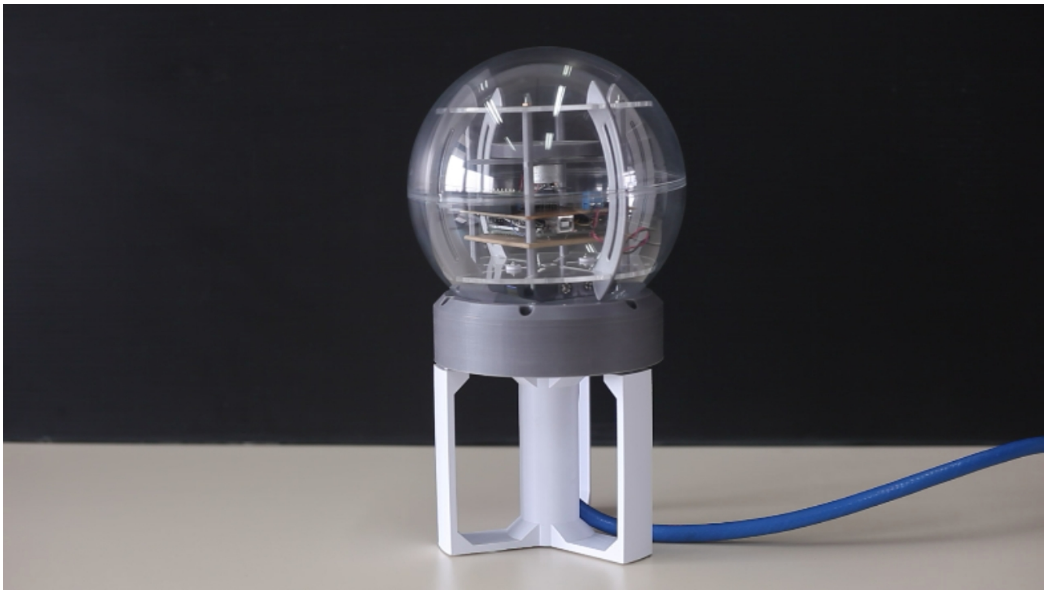

Fig. S1: Photograph of the of the setup used for experimental verification of the proposed algorithm.

The Block diagram of the setup is shown in Fig. S2. The ADCS model consists of a microcontroller, an MPU9250 MARG sensor comprising a three-axis accelerometer, magnetometer, and gyroscope, a DC reaction wheel motor with its corresponding driver circuitry, and a Bluetooth wireless communication interface. For the experiments presented in this work, the reaction wheel motor is not actively used for attitude control. Nevertheless, its permanent-magnet structure produces a magnetic field in the vicinity of the magnetometer. This field introduces a significant hard-iron disturbance to the measured local geomagnetic field, making magnetometer calibration necessary prior to attitude estimation.

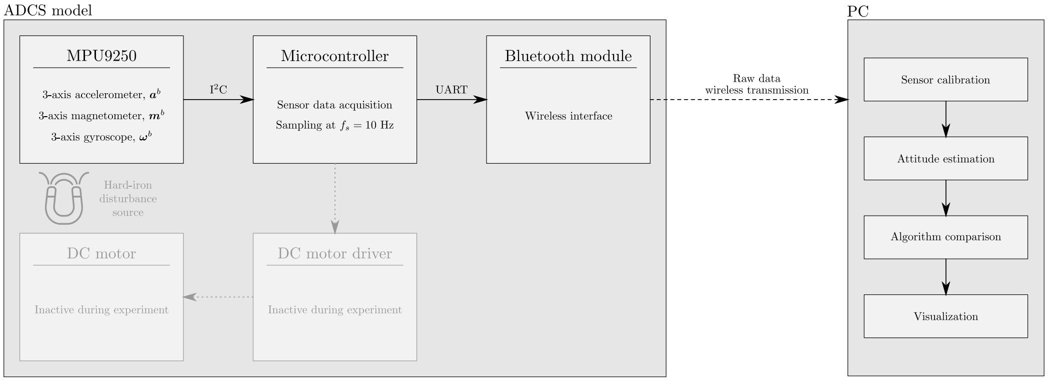

Fig. S2: Block diagram of the setup used for experimental verification of the proposed algorithm.

During the experiments, the microcontroller is used exclusively for sensor data acquisition and wireless transmission of the raw MPU9250 measurements to a computer via the Bluetooth communication interface. No attitude estimation or additional sensor-data processing is performed on the microcontroller. All subsequent processing, including sensor calibration and execution of the attitude estimation algorithms, is performed offline on the computer.

The use of offline processing allows the proposed attitude estimation algorithm to be evaluated in greater detail and compared directly with other attitude estimation algorithms using identical input measurements. In this way, differences in the obtained attitude estimates can be attributed to the algorithms themselves rather than to differences in sensor measurements or experimental conditions.

2 Sensor Calibration

The MPU9250 MARG sensor, comprising a three-axis magnetometer, accelerometer, and gyroscope, is calibrated using a set of static measurements. For the magnetometer and accelerometer calibration, measurements are collected at a large number of different sensor orientations. To obtain these measurements, the ADCS model is rotated by a small angular increment about different axes between consecutive samples. After each rotation, sufficient time is allowed for the system to mechanically stabilize before the next sample is acquired. Consequently, the measurements used for calibration can be considered static. A total of 278 static samples are collected for both the magnetometer and the accelerometer.

For the gyroscope calibration, the ADCS model is kept stationary in a single arbitrary orientation while the measurements are collected. Since the ideal angular velocity of a stationary sensor is zero, these measurements can be used to determine the gyroscope bias. A total of 284 static gyroscope samples are collected.

The calibration parameters are estimated from the collected static measurements using MathWorks MATLAB.

2.1 Magnetometer Calibration

MathWorks MATLAB's `magcal` function, available in the Navigation Toolbox, estimates magnetometer calibration parameters from a set of uncalibrated three-axis magnetic field measurements. Ideally, magnetometer measurements collected at different sensor orientations should lie on a sphere centered at the origin. In practice, hard-iron and soft-iron effects distort this sphere into an offset ellipsoid.

The `magcal` function fits an ellipsoid to the uncalibrated measurements and estimates the hard-iron bias vector $\boldsymbol{b}_m$ and the soft-iron correction matrix $\boldsymbol{A}_m$. Assuming that individual magnetometer measurements are represented as row vectors, the calibrated magnetic field measurement $\boldsymbol{m}_\mathrm{c}$ is obtained as

$$\boldsymbol{m}_\mathrm{c} = (\boldsymbol{m}_\mathrm{u} - \boldsymbol{b}_m)\,\boldsymbol{A}_m, \tag{S1}$$

where $\boldsymbol{m}_\mathrm{u} \in \mathbb{R}^{1\times3}$ is the uncalibrated magnetometer measurement expressed in μT, $\boldsymbol{m}_\mathrm{c} \in \mathbb{R}^{1\times3}$ is the calibrated magnetometer measurement expressed in μT, $\boldsymbol{b}_m \in \mathbb{R}^{1\times3}$ is the estimated hard-iron bias vector expressed in μT, and $\boldsymbol{A}_m \in \mathbb{R}^{3\times3}$ is the dimensionless soft-iron correction matrix.

Subtracting $\boldsymbol{b}_m$ from each sample of magnetic field compensates for the hard-iron offset, while multiplication by $\boldsymbol{A}_m$ compensates for the soft-iron distortion. After calibration, the measurements ideally lie on a sphere centered at the origin. The radius of this sphere, r_m, returned by the `magcal` function, represents the estimated magnetic field magnitude associated with the calibration data and is expressed in μT.

Based on the collected magnetometer measurements, the following calibration parameters are obtained:

$$\boldsymbol{A}_m = \begin{bmatrix} 0.9688 & -0.0505 & -0.0319 \\ -0.0505 & 0.9924 & 0.0593 \\ -0.0319 & 0.0593 & 1.0473 \end{bmatrix}, \tag{S2}$$

$$\boldsymbol{b}_m = \begin{bmatrix} 124.8244 & 102.1960 & -72.3186 \end{bmatrix}\ \mu\mathrm{T}, \tag{S3}$$

$$r_m = 30.5960\ \mu\mathrm{T}. \tag{S4}$$

It should be noted that the reference magnetic field vector used in the proposed algorithm is $\boldsymbol{m}^i = [22.2,\ 1.7,\ 42.7]^T\ \mu\text{T}$, with a magnitude of $\|\boldsymbol{m}^i\| = 48.1562\ \mu\text{T}$. Although the magnitude of the reference magnetic field differs from the value r_m obtained during calibration, this discrepancy does not affect the proposed attitude estimation algorithm. The algorithm uses only the direction of the magnetic field rather than its magnitude. Therefore, both the reference magnetic field vector and each calibrated magnetometer measurement are normalized to unit length before being provided to the algorithm.

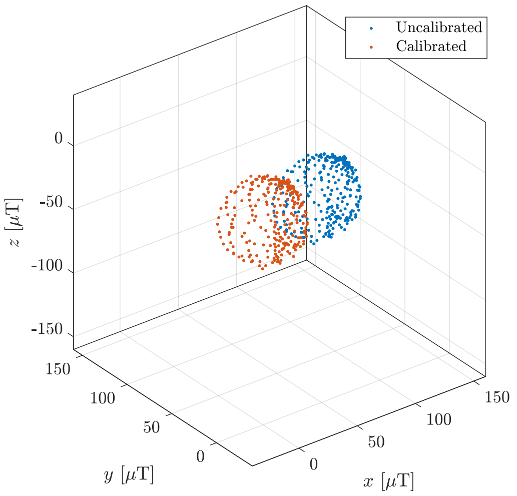

(a) 3D view of the static magnetometer measurements.

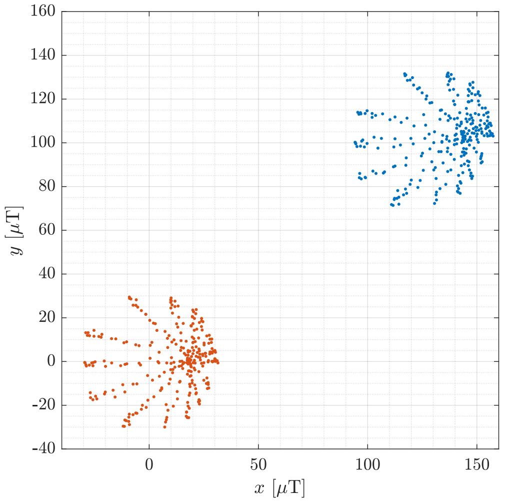

(b) Static magnetometer measurements in the x-y plane.

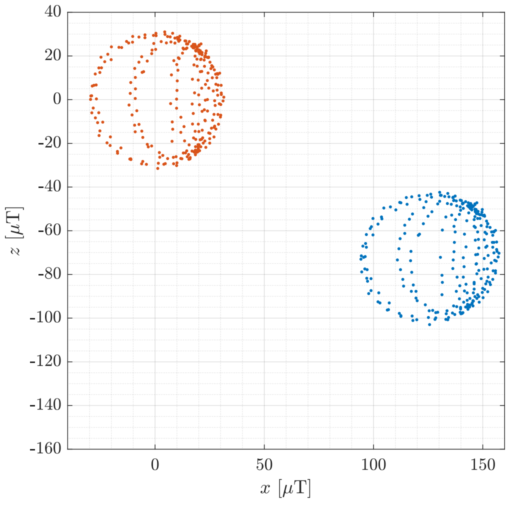

(c) Static magnetometer measurements in the x-z plane.

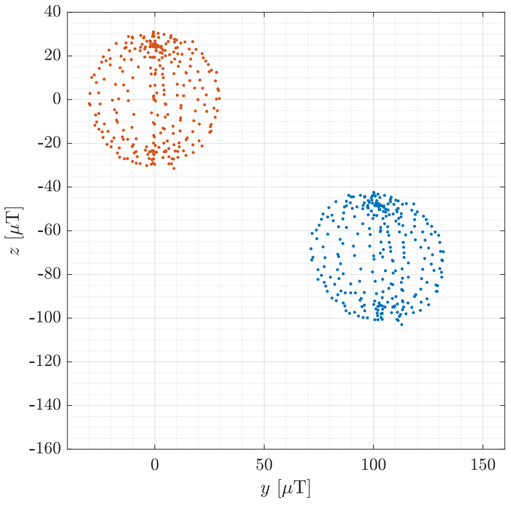

(d) Static magnetometer measurements in the y-z plane.

Fig. S3: Uncalibrated (blue) and calibrated (red) static magnetometer measurements.

Fig. S3 shows the static magnetometer measurements used to estimate the hard-iron bias vector $\boldsymbol{b}_m$ and the soft-iron correction matrix $\boldsymbol{A}_m$. For clarity, the measurements are presented both in

three-dimensional space (Fig. S3a) and projected onto the x-y (Fig. S3b), x-z (Fig. S3c), and y-z (Fig. S3d) planes. The uncalibrated measurements form an offset ellipsoid, whereas the calibrated measurements form an approximately spherical distribution centered at the origin. In particular, the projections onto the coordinate planes clearly illustrate the displacement of the uncalibrated ellipsoid from the origin. This displacement corresponds to the estimated hard-iron bias vector $\boldsymbol{b}_m = [124.8244,\ 102.1960,\ -72.3186]\ \mu$T, whose components represent the offsets along the x, y, and z axes, respectively. The calibration removes this offset and simultaneously corrects the ellipsoidal distortion through the soft-iron correction matrix $\boldsymbol{A}_m$.

In the absence of soft-iron distortion, no correction of the shape of the measurement distribution would be required and $\boldsymbol{A}_m$ would ideally be the identity matrix. The estimated matrix has diagonal elements close to unity and relatively small off-diagonal elements. The deviation of the diagonal elements from unity represents axis-dependent scaling, while the nonzero off-diagonal elements account for cross-axis coupling required to transform the ellipsoidal measurement distribution into a sphere.

Fig. S4 shows the x, y, and z components of the same uncalibrated and calibrated magnetometer measurements as a function of the sample index.

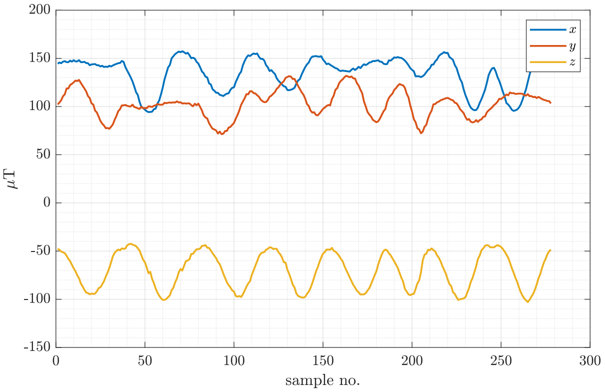

(a) Uncalibrated static magnetometer measurements.

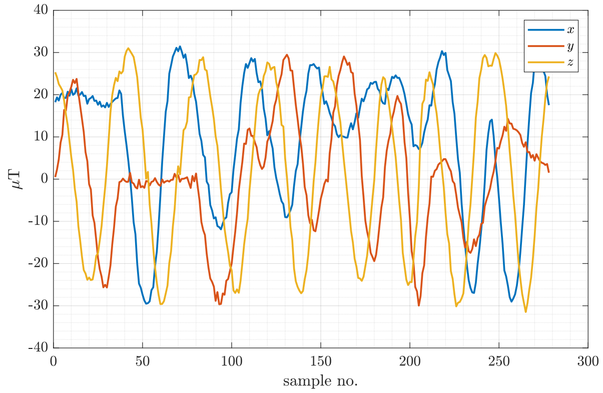

(b) Calibrated static magnetometer measurements.

Fig. S4: Comparison between the uncalibrated and calibrated static magnetometer measurements.

The norms of the uncalibrated and calibrated magnetometer measurements shown in Fig. S4 are presented in Fig. S5. A clear difference between the two sets of measurements can be observed. The magnitude of the uncalibrated magnetic field varies considerably with sensor orientation due

to hard-iron and soft-iron distortions. In contrast, the magnitude of the calibrated magnetic field remains approximately constant regardless of sensor orientation and is approximately equal to the estimated calibration radius $r_m = 30.5960\ \mu$T. This behavior is consistent with the expected result of a successful magnetometer calibration, since rotating a stationary sensor within a spatially uniform and constant magnetic field changes the measured field direction but not its magnitude.

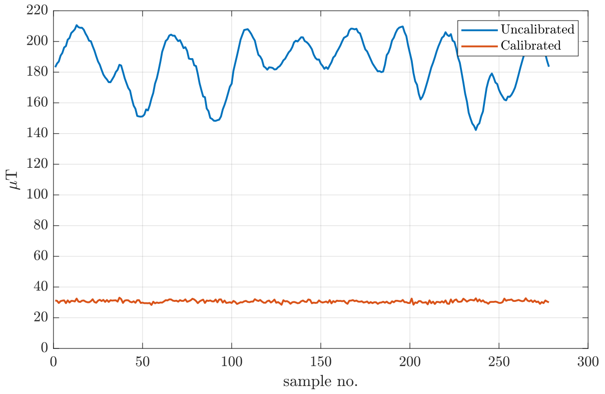

Fig. S5: Comparison between the norms of the uncalibrated and calibrated static magnetometer measurements.

2.2 Accelerometer Calibration

The accelerometer is calibrated using the same ellipsoid-fitting approach described previously for the magnetometer. Since the measurements used for both calibrations are acquired simultaneously at the same static sensor orientations, the same set of 278 samples is used. For a stationary accelerometer, the measured acceleration is determined by gravity and, ideally, measurements obtained at different sensor orientations should therefore lie on a sphere whose radius corresponds to the magnitude of the gravitational acceleration. Accelerometer bias, scale-factor errors, and cross-axis effects cause deviations from this ideal spherical distribution.

The `magcal` function is therefore also applied to the accelerometer measurements. Although originally intended for magnetometer calibration, the underlying ellipsoid-fitting procedure is equally applicable in this case, as the calibration is based on the geometric distribution of the three-axis measurements rather than on the physical nature of the measured quantity. Following the same formulation as in the magnetometer calibration, the calibrated accelerometer measurement is obtained as

$$\boldsymbol{a}_{\mathrm{c}} = (\boldsymbol{a}_{\mathrm{u}} - \boldsymbol{b}_a)\, \boldsymbol{A}_a, \tag{S5}$$

where $\boldsymbol{a}_{\mathrm{u}} \in \mathbb{R}^{1\times3}$ and $\boldsymbol{a}_{\mathrm{c}} \in \mathbb{R}^{1\times3}$ are the uncalibrated and calibrated accelerometer measurements expressed in $\mathrm{m/s^2}$, respectively, $\boldsymbol{b}_a \in \mathbb{R}^{1\times3}$ is the estimated accelerometer bias vector expressed in $\mathrm{m/s^2}$, and $\boldsymbol{A}_a \in \mathbb{R}^{3\times3}$ is the dimensionless correction matrix.

Based on the collected accelerometer measurements, the following calibration parameters are obtained:

$$\boldsymbol{A}_a = \begin{bmatrix} 1.0038 & 0.0002 & 0.0016 \\ 0.0002 & 1.0047 & 0.0006 \\ 0.0016 & 0.0006 & 0.9915 \end{bmatrix}, \tag{S6}$$

$$\boldsymbol{b}_a = \begin{bmatrix} 0.1020 & 0.1142 & 0.6971 \end{bmatrix} \ \mathrm{m/s^2}, \tag{S7}$$

$$r_a = 9.8696 \ \mathrm{m/s^2}. \tag{S8}$$

The estimated correction matrix $\boldsymbol{A}_a$ is close to the identity matrix, with diagonal elements close to unity and very small off-diagonal elements. Compared with the magnetometer calibration, this indicates considerably smaller ellipsoidal distortion of the accelerometer measurements. Nevertheless, the estimated bias vector $\boldsymbol{b}_a$ indicates a noticeable displacement of the measurement distribution from the origin, particularly along the z-axis.

The reference acceleration vector used in the proposed algorithm is $\boldsymbol{a}^i = [0,\ 0,\ 9.81]^T$ $\mathrm{m/s^2}$, with a magnitude of $\|\boldsymbol{a}^i\| = 9.81$ $\mathrm{m/s^2}$. The estimated calibration radius $r_a = 9.8696$ $\mathrm{m/s^2}$ is close to this reference value. As with the magnetic field measurements, the proposed attitude estimation algorithm uses the direction of the acceleration vector rather than its magnitude. Therefore, both the reference acceleration vector and each calibrated accelerometer measurement are normalized to unit length before being provided to the algorithm.

Fig. S6 shows the uncalibrated and calibrated static accelerometer measurements. As in the magnetometer calibration, the measurements are shown in three-dimensional space and projected onto the three coordinate planes. The uncalibrated measurements exhibit a displacement from the origin corresponding to the estimated bias vector $\boldsymbol{b}_a = [0.1020,\ 0.1142,\ 0.6971]$ $\mathrm{m/s^2}$. The comparatively small deformation of the uncalibrated measurement distribution is consistent with the correction matrix $\boldsymbol{A}_a$ being close to the identity matrix. After calibration, the measurements

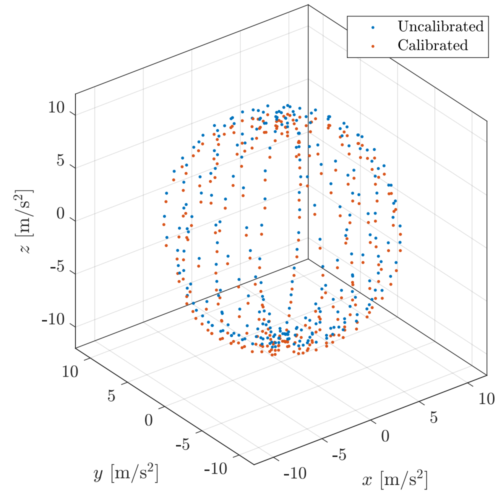

(a) 3D view of the static accelerometer measurements.

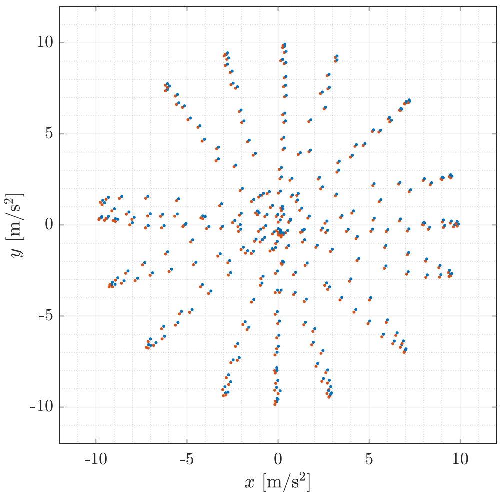

(b) Static accelerometer measurements in the x-y plane.

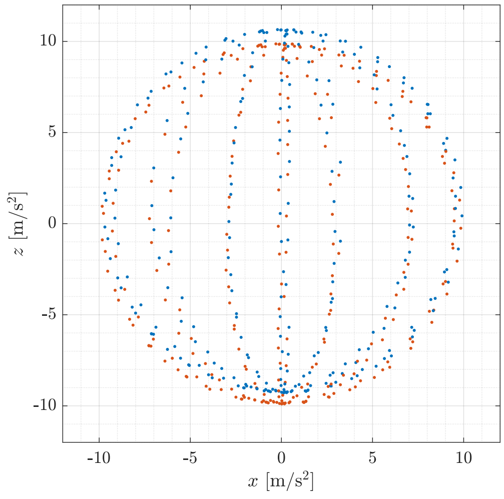

(c) Static accelerometer measurements in the x-z plane.

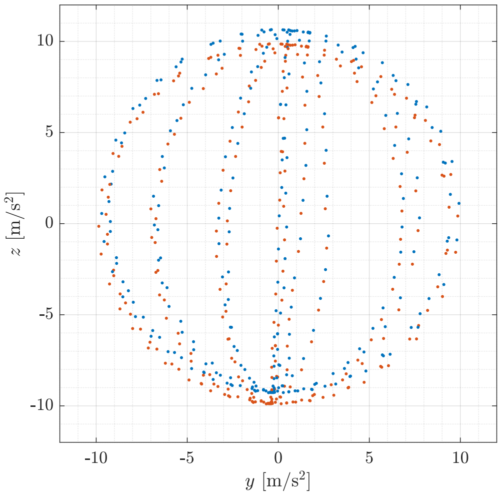

(d) Static accelerometer measurements in the y-z plane.

Fig. S6: Uncalibrated (blue) and calibrated (red) static accelerometer measurements.

form an approximately spherical distribution centered at the origin.

Fig. S7 shows the x, y, and z components of the same uncalibrated and calibrated accelerometer measurements as a function of the sample index.

The norms of the uncalibrated and calibrated accelerometer measurements are shown in Fig. S8. The uncalibrated measurements exhibit variations in magnitude with sensor orientation, although these variations are considerably smaller than those observed for the magnetometer. After calibration, the magnitude remains approximately constant and is approximately equal to the estimated calibration radius $r_a = 9.8696$ m/s^2. This behavior is consistent with the expected result for a stationary accelerometer, since changing the sensor orientation with respect to the gravity vector

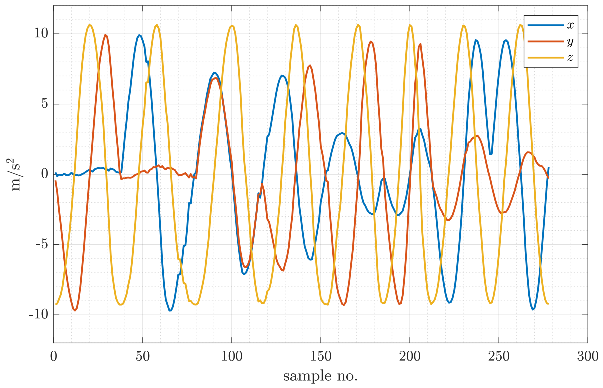

(a) Uncalibrated static accelerometer measurements.

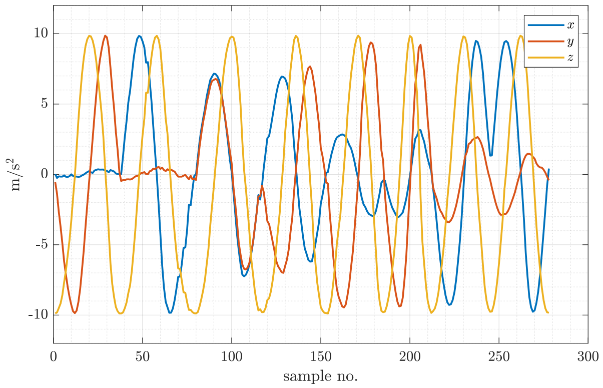

(b) Calibrated static accelerometer measurements.

Fig. S7: Comparison between the uncalibrated and calibrated static accelerometer measurements.

changes its measured components but not its magnitude.

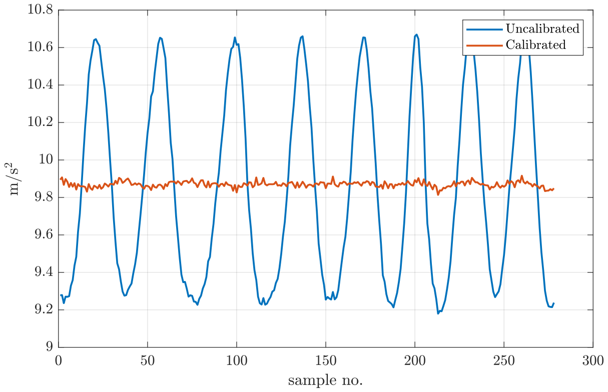

Fig. S8: Comparison between the norms of the uncalibrated and calibrated static accelerometer measurements.

2.3 Gyroscope Calibration

Unlike the magnetometer and accelerometer, the gyroscope does not require measurements at different sensor orientations for bias calibration. Instead, the ADCS model is kept stationary in a single arbitrary orientation while the gyroscope measurements are collected. Since the sensor is stationary, the true angular velocity is expected to be zero along all three axes. Therefore, any nonzero mean value of the measured angular velocity can be attributed to the gyroscope bias.

The gyroscope bias vector $\boldsymbol{b}_\omega$ is estimated as the mean of the collected static measurements. The calibrated angular velocity $\boldsymbol{\omega}_\mathrm{c}$ is then obtained by subtracting the estimated bias from each uncalibrated measurement:

$$\boldsymbol{\omega}_\mathrm{c} = \boldsymbol{\omega}_\mathrm{u} - \boldsymbol{b}_\omega, \tag{S9}$$

where $\boldsymbol{\omega}_\mathrm{u} \in \mathbb{R}^{1\times3}$ and $\boldsymbol{\omega}_\mathrm{c} \in \mathbb{R}^{1\times3}$ are the uncalibrated and calibrated gyroscope measurements expressed in rad/s, respectively, and $\boldsymbol{b}_\omega \in \mathbb{R}^{1\times3}$ is the estimated gyroscope bias vector expressed in rad/s.

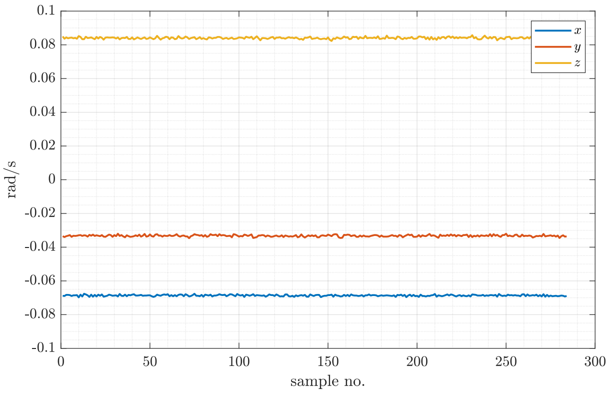

(a) Uncalibrated static gyroscope measurements.

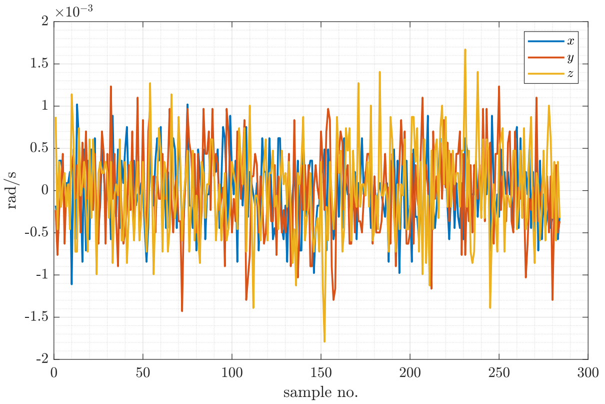

(b) Calibrated static gyroscope measurements.

Fig. S9: Comparison between the uncalibrated and calibrated static gyroscope measurements.

Based on the collected static gyroscope measurements, the estimated bias vector is

$$\boldsymbol{b}_\omega = \begin{bmatrix} -0.0686 & -0.0333 & 0.0841 \end{bmatrix} \text{ rad/s}. \tag{S10}$$

Fig. S9 shows the x, y, and z components of the uncalibrated and calibrated static gyroscope measurements. The uncalibrated measurements exhibit a nonzero offset along all three axes, corresponding to the estimated bias vector $\boldsymbol{b}_{\omega}$. After subtracting the estimated bias, the calibrated measurements are centered around zero, as expected for a stationary sensor.

The norms of the uncalibrated and calibrated gyroscope measurements are shown in Fig. S10. Since the true angular velocity is zero during the calibration procedure, the norm of an ideal gyroscope measurement would also be zero. The calibrated measurements exhibit a substantially reduced norm compared with the uncalibrated measurements, with the remaining nonzero values resulting primarily from measurement noise. This confirms that the constant gyroscope bias is effectively removed by the calibration.

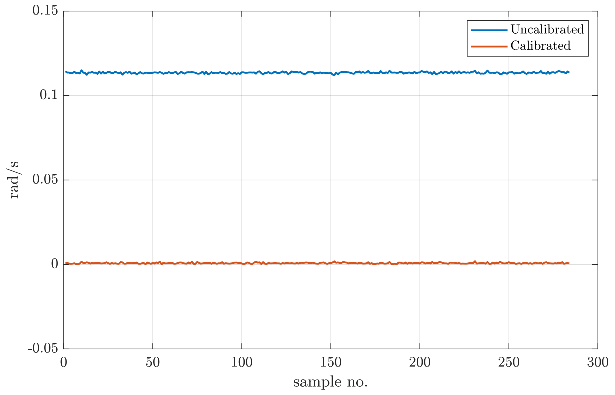

Fig. S10: Comparison between the norms of the uncalibrated and calibrated static gyroscope measurements.

3 Calibration of Experimental Measurements

Following the determination of the sensor calibration parameters, the experimental measurements used for verification of the proposed attitude estimation algorithm are calibrated using the parameters obtained in the previous section. The calibration parameters are determined exclusively from the previously collected static calibration data and remain unchanged throughout the experimental measurements.

During the experiment, the MPU9250 sensor measurements are sampled at a frequency of 10 Hz, wirelessly transmitted, and stored to a computer for subsequent processing using the implemented attitude estimation algorithms. The ADCS platform is manually rotated by approximately $\pm 45°$ about each of the three mutually orthogonal axes. The rotations are performed sequentially, first about the z-axis, followed by the y-axis, and finally about the x-axis.

Prior to attitude estimation, the stored raw experimental measurements are calibrated using the previously determined calibration parameters. The magnetometer and accelerometer measurements are corrected according to Equ. S1 and Equ. S5, respectively, using the corresponding bias vectors and correction matrices. Similarly, the gyroscope bias is removed from the raw angular velocity measurements according to Equ. S9. No additional calibration parameters are estimated from the experimental data.

Fig. S11 shows the uncalibrated and calibrated magnetometer, accelerometer, and gyroscope measurements obtained during the experiment. The effect of the previously determined calibration parameters can be observed by comparing the uncalibrated measurements in the left column with the corresponding calibrated measurements in the right column.

The norms of the uncalibrated and calibrated experimental measurements are shown in Fig. S12. Unlike the static measurements used for sensor calibration, the measurements collected during the experiment are obtained while the platform is being manually rotated. Consequently, the norms of the calibrated sensor measurements are not perfectly constant throughout the experiment.

For the magnetometer, an ideal rotation at a fixed position in a spatially uniform and constant magnetic field would change only the direction of the measured magnetic field vector, while its magnitude would remain constant. In practice, however, the local magnetic field may not be perfectly homogeneous due to nearby ferromagnetic materials, electrical equipment, current-carrying conductors, and other sources of magnetic disturbance. Furthermore, manual manipulation of the platform can introduce small translational displacements, causing the sensor to experience spatial variations of the local magnetic field. Consequently, variations in the norm of the calibrated magnetometer measurements may remain even after the sensor-specific hard-iron and soft-iron effects have been compensated.

For the accelerometer, the static calibration procedure assumes that the measured acceleration is determined only by gravity. During the experiment, this assumption is no longer valid, since manual rotation of the platform also introduces translational and rotational accelerations. The accelerometer therefore measures a combination of the gravitational contribution and motion-induced specific force. As a result, the magnitude of the calibrated accelerometer measurements can temporarily deviate from the gravitational acceleration during platform motion, even when the sensor itself is properly calibrated.

The remaining variations in the calibrated magnetometer and accelerometer measurements provide an additional challenge for the proposed attitude estimation algorithm and are therefore particularly relevant for its experimental verification. In the analyses presented in the manuscript, the algorithm was evaluated using synthetic static measurements without noise and synthetic dynamic measurements affected by measurement noise. The experimental measurements extend this evaluation by additionally exposing the algorithm to perturbations that naturally arise from the

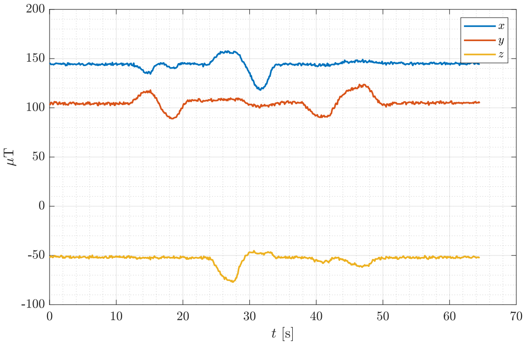

(a) Uncalibrated magnetometer measurements.

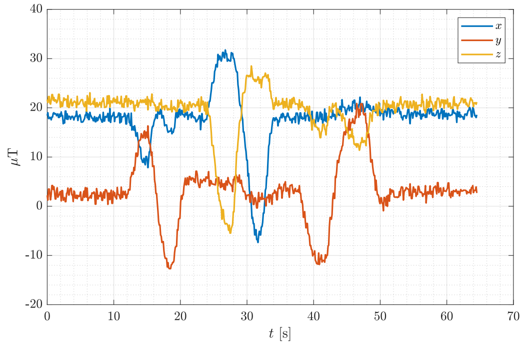

(b) Calibrated magnetometer measurements.

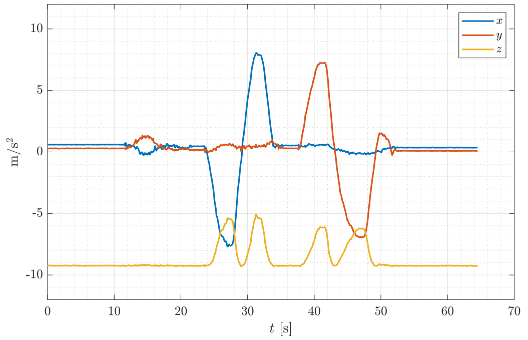

(c) Uncalibrated accelerometer measurements.

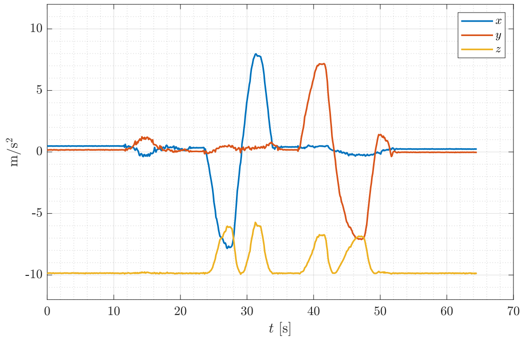

(d) Calibrated accelerometer measurements.

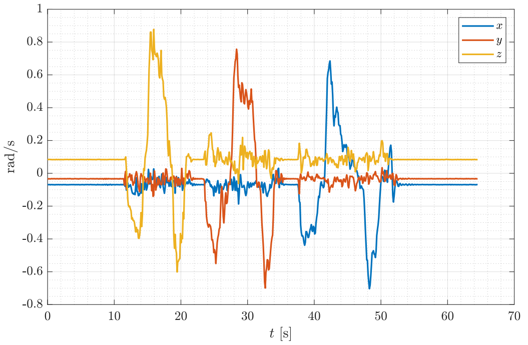

(e) Uncalibrated gyroscope measurements.

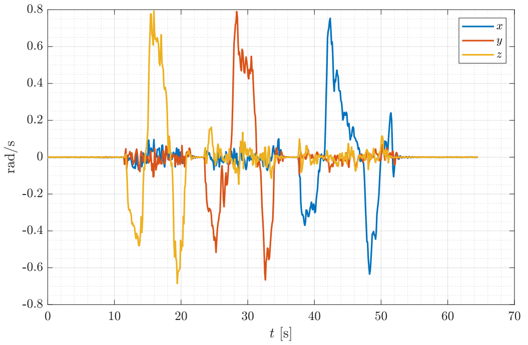

(f) Calibrated gyroscope measurements.

Fig. S11: Comparison between the uncalibrated and calibrated magnetometer, accelerometer, and gyroscope measurements used for experimental verification of the proposed attitude estimation algorithm.

actual sensor motion and the surrounding environment, including local magnetic field disturbances and motion-induced accelerometer components. These effects introduce deviations from the ideal reference-vector assumptions beyond those considered in the synthetic tests and therefore provide an additional means of evaluating the robustness of the proposed algorithm under realistic operating conditions.

The gyroscope measurements have a different interpretation. During static calibration, the expected angular velocity is zero, whereas during the experiment the platform is intentionally rotated. Therefore, the norm of the calibrated gyroscope measurements is expected to increase during peri-

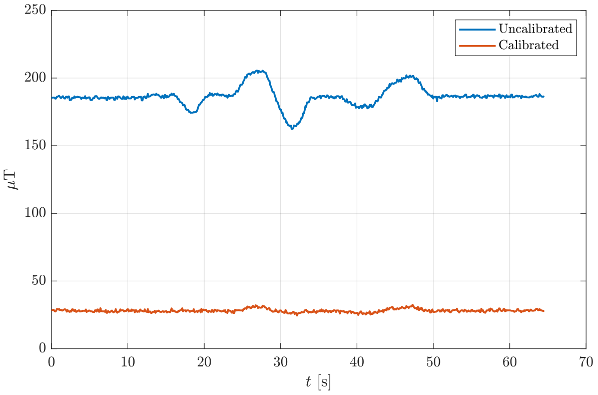

(a) Norm of the magnetometer measurements.

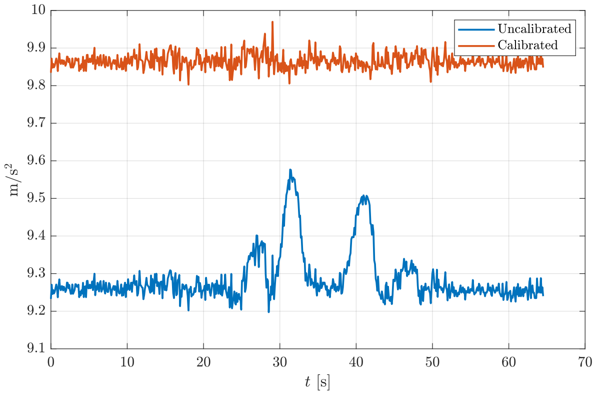

(b) Norm of the accelerometer measurements.

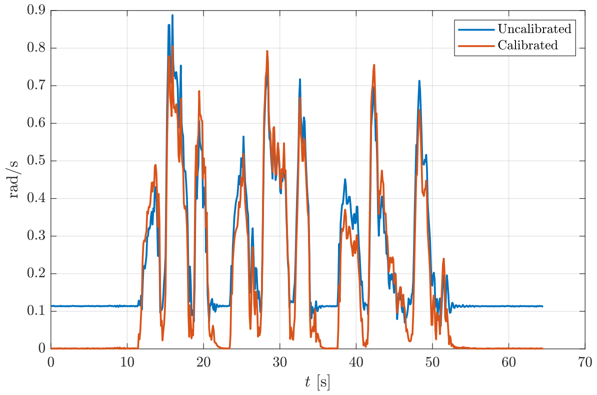

(c) Norm of the gyroscope measurements.

Fig. S12: Comparison between the norms of the uncalibrated and calibrated magnetometer, accelerometer, and gyroscope measurements used for experimental verification of the proposed attitude estimation algorithm.

ods of rotation and approach zero when the platform is stationary. Gyroscope calibration removes the estimated constant bias but does not, and should not, remove the actual angular velocity of the platform.

These variations are therefore not necessarily an indication of inadequate sensor calibration.

The calibration compensates for the systematic sensor errors identified from the static calibration measurements, while the experimental measurements additionally contain physical effects associated with the actual motion of the platform and the surrounding environment.

4 Calibrated Data Used in Experimental Verification

For completeness and reproducibility, the calibrated measurements obtained from the MPU9250 MARG sensor and used in the experimental verification of the proposed algorithm are provided below. The measurements are sampled with a sampling period of $\Delta T = 0.1$ s. Each row contains nine comma-separated values corresponding to a single time step. The first three values represent the calibrated accelerometer measurements expressed in m/s^2 (a_x, a_y, and a_z, respectively), the next three values represent the calibrated magnetometer measurements expressed in μT (m_x, m_y, and m_z, respectively), and the final three values represent the calibrated gyroscope measurements expressed in rad/s (ω_x, ω_y, and ω_z, respectively).

```
0.487729186072811,0.163444652200863,-9.82085200818638,18.418845939461,2.20477287244472,21.6693341646795,-0.00164341901408448,0.000435066901408453,-0.000591478873239432
0.487666580696293,0.153799814937651,-9.85884223065795,18.9959657379207,1.26396633285318,21.0622453114019,-0.000311419014084477,-0.000896933098591547,0.000207521126760563
0.468478714808099,0.163431527822955,-9.8351264880696,17.8237201786772,3.4989704655762,22.2975421793379,-0.000311419014084477,0.00016906690140845,0.000207521126760563
0.482885453125094,0.168240971940401,-9.84459694912133,17.8237201786772,3.4989704655762,22.2975421793379,8.85809859155207e-05,-0.00049693309859155,0.000739521126760567
0.4829028455573,0.177868848917661,-9.83509540146232,18.0787896384424,2.53553107685954,20.9853137170247,-0.00150941901408447,-0.000629933098591551,7.45211267605683e-05
0.482892989137732,0.16824379885149,-9.83984850614216,17.9752735020989,1.29748398640542,20.737952079573,-0.000444419014084471,-0.000363933098591548,-0.000192478873239435
0.482881400228041,0.182671983008432,-9.84933691195752,17.5556469841437,2.41616308274723,21.52905456568,-0.000311419014084477,0.000701066901408448,-0.000458478873239437
0.487670063811879,0.168233652916772,-9.85883375051497,18.1206989726608,4.20810468960777,22.6866875042245,-0.000178419014084469,3.60669014084491e-05,-0.000591478873239432
0.473261003498646,0.153801985053953,-9.84936894269514,18.1206989726608,4.20810468960777,22.6866875042245,-0.00124341901408448,-9.79330985915461e-05,-0.000858478873239435
0.478108376877338,0.168251117284827,-9.82561269627973,18.8771114852064,3.40460619977034,21.5471607925348,-0.000577419014084479,0.000302066901408451,-0.000325478873239443
0.478067809514812,0.187478605079915,-9.85408907309731,19.0057128306407,3.95614922319638,22.1105161494202,-0.000844419014084469,0.000435066901408453,-0.000325478873239443
0.478033023045391,0.168222850535102,-9.87309315994653,18.7376250215778,2.87334364992895,21.3420301203392,-0.00177641901408447,0.00016906690140845,-0.000858478873239435
0.482880239108694,0.177860368774685,-9.84933973886861,18.7986950900916,1.48005428440474,21.6157923965283,-0.000444419014084471,-0.00049693309859155,-0.000991478873239443
0.478075913977765,0.15861758707568,-9.84461013836585,18.7986950900916,1.48005428440474,21.6157923965283,-0.000710419014084473,-9.79330985915461e-05,0.00100652112676056
0.487679921820755,0.177858703573235,-9.85407965430392,18.3866680508923,3.2243841287193,21.016234926525,-0.000311419014084477,0.000435066901408453,-0.000591478873239432
0.487677599824517,0.168236479827862,-9.85408530753581,18.5261545145209,3.75564667856067,21.2213655987205,-0.000844419014084469,0.00016906690140845,-0.00072447887323944
0.473267378391937,0.148993197731295,-9.84462332662706,18.7807024835406,1.83345040698942,21.6369083256876,-0.000178419014084469,-0.000230933098591547,-5.94788732394408e-05
0.482863416442255,0.139367642750273,-9.8541098038344,18.0422835463551,2.28355272371661,22.7553190763522,-0.000311419014084477,-0.000762933098591552,7.45211267605683e-05
0.473263325494884,0.163424208799326,-9.84936328946325,18.0422835463551,2.28355272371661,22.7553190763522,-0.00124341901408448,0.000568066901408454,-0.000991478873239443
0.478083449990403,0.15862041398677,-9.83986169538669,18.7376250215778,2.87334364992895,21.3420301203392,-0.000444419014084471,3.60669014084491e-05,-0.000192478873239435
0.478077074854656,0.163428196587307,-9.84460731204505,18.2509741721034,3.00594712426911,20.1157708497462,-0.000444419014084471,0.000568066901408454,-0.00072447887323944
0.492469902023265,0.173042596394201,-9.86357183273431,19.171451335029,1.08844959509948,21.2251413355616,-0.000844419014084469,-0.000363933098591548,-0.000458478873239437
0.47804925437395,0.173039113278616,-9.86359443919862,18.4084674207419,3.18381974031564,20.2997855298677,-0.00124341901408448,3.60669014084491e-05,-5.94788732394408e-05
0.482876755993109,0.163426530795565,-9.84934821901158,18.4084674207419,3.18381974031564,20.2997855298677,-4.54190140844746e-05,0.000302066901408451,-0.000858478873239435
0.473248255043216,0.16341855556744,-9.85885918389037,16.7644032341941,1.21398252085521,21.3043135461755,-0.000577419014084479,3.60669014084491e-05,-0.00072447887323944
0.473270861507522,0.163427035710416,-9.84461484648408,17.4123392250785,1.57206955660823,22.0192578389712,-0.000178419014084469,-0.00049693309859155,0.000473521126760565
0.482895879341829,0.14900117295942,-9.83511236174827,16.9039042804862,1.7452433204313,21.5094436810862,-0.000844419014084469,0.00110106690140845,-0.000325478873239443
0.478095628421909,0.177867687798314,-9.83510293747496,19.3360220389393,0.933208128438344,21.7462657590568,-4.54190140844746e-05,-0.000230933098591547,0.000340521126760557
0.492475116039665,0.163423199559917,-9.85882904298703,19.3360220389393,0.933208128438344,21.7462657590568,-0.00124341901408448,-0.000363933098591548,-5.94788732394408e-05
0.468483359043032,0.182676980035823,-9.83511518101554,17.7131260729829,2.59402857691828,21.7130702967122,-0.00111041901408447,0.00016906690140845,0.000340521126760557
0.478086932863533,0.17305324724377,-9.839853215834,18.7703239648216,2.81249727486034,20.2673596908757,-0.000977419014084477,0.000701066901408448,0.000473521126760565
0.482887775121332,0.177863195685775,-9.84459129588944,17.6373495403126,3.69476843211143,22.4928667099959,-0.00111041901408447,0.000435066901408453,-0.000458478873239437
0.497285816306341,0.177858198658384,-9.85881302683142,18.2869586778475,2.2991687724462,20.0735398215753,-0.000977419014084477,-0.000230933098591547,-0.000591478873239432
0.48767122468877,0.173044262428399,-9.85883092419417,18.2869586778475,2.2991687724462,20.0735398215753,-0.000444419014084471,0.00110106690140845,-5.94788732394408e-05
0.478058521287403,0.1489887053763,-9.85411168661515,18.2111821451772,3.39990862763936,20.853336234859,-0.000577419014084479,0.000701066901408448,0.000739521126760567
0.468449735024111,0.168231835592929,-9.85411545001275,17.6700486110201,3.63392181985781,21.4181920913639,-0.000577419014084479,-9.79330985915461e-05,-0.000458478873239437
0.482862846660788,0.168232491797425,-9.85884128652761,18.0030132013699,3.63627075416393,21.765106988432,-4.54190140844746e-05,-0.000762933098591552,-5.94788732394408e-05
0.482915594028429,0.168252278404174,-9.82560516026709,18.2760590088556,2.31945093699991,20.4317639962579,-0.000311419014084477,-9.79330985915461e-05,-0.000458478873239437
0.487701367165714,0.173055569482464,-9.83983814380873,18.2760590088556,2.31945093699991,20.4317639962579,-0.000577419014084479,-0.00049693309859155,0.000207521126760563
0.482906898470053,0.16343783784963,-9.83035543862613,17.9823660859791,0.96437697504766,21.0750607401702,0.000221580985915529,-0.00049693309859155,-0.00072447887323944
0.482881970009508,0.15380713396128,-9.8446054292643,17.5305620517937,3.1026594479052,21.2130645610447,-0.000844419014084469,0.000302066901408451,-0.000192478873239435
0.478094467302562,0.173056073564567,-9.83510576438605,18.0498974079718,2.90921017849106,21.3646507382864,-0.000178419014084469,0.000701066901408448,-0.000591478873239432
0.482856471525041,0.173040274397963,-9.86358690318598,18.2902445489545,1.65322903452553,21.1059818903742,-0.00111041901408447,0.000435066901408453,-0.000591478873239432
0.478111859992924,0.182684955263947,-9.82560421613675,18.2902445489545,1.65322903452553,21.1059818903742,-0.000444419014084471,-0.000363933098591548,0.000340521126760557
0.468447413027873,0.158609611847556,-9.85412110324464,19.3185505826611,2.24536895434025,20.0396088787216,-0.000311419014084477,-9.79330985915461e-05,-0.000192478873239435
0.487677599824517,0.168236479827862,-9.85408530753581,18.4330314290343,1.53855196235224,22.3435521180922,-0.000844419014084469,-9.79330985915461e-05,-0.000325478873239443
0.482915594028429,0.168252278404174,-9.82560516026709,17.419953009757,2.1977262561858,20.6285936307776,-0.000178419014084469,-0.00156193309859155,-5.94788732394408e-05
0.482848937086012,0.173037448077166,-9.86833435463393,18.2831519650743,1.98633604588328,20.7688732297771,-0.000311419014084477,-9.79330985915461e-05,0.000207521126760563
0.492461206707345,0.168229160561777,-9.86832211050306,18.2831519650743,1.98633604588328,20.7688732297771,-0.00137641901408447,-0.00049693309859155,-0.000991478873239443
0.473266808610469,0.177858046778447,-9.84935480932027,18.5981239302136,2.34208203585973,21.1369030680086,-0.000844419014084469,0.000701066901408448,0.000207521126760563
0.482877917112456,0.168238145029312,-9.84934539210049,18.0498974079718,2.90921017849106,21.3646507382864,-0.000844419014084469,0.000302066901408451,0.000872521126760561
0.482862846660788,0.168232491797425,-9.85884128652761,17.0973678245439,1.21633145516133,21.6512284432437,-0.000444419014084471,0.000302066901408451,-0.000458478873239437
0.482876755993109,0.163426530795565,-9.84934821901158,17.0973678245439,1.21633145516133,21.6512284432437,8.85809859155207e-05,0.00016906690140845,0.000473521126760565
0.473255791055855,0.163421382478529,-9.8541107409112,17.0973678245439,1.21633145516133,21.6512284432437,-0.000577419014084479,0.00016906690140845,-0.000591478873239432
0.468425967440458,0.163411741216207,-9.86836261433012,17.2335819353221,2.39353222107241,20.8239196830977,-0.000710419014084473,3.60669014084491e-05,0.000739521126760567
0.482883131128855,0.158618748195027,-9.84460260235321,17.5523614216434,3.06209505950153,20.4966151643874,-0.00150941901408447,0.00016906690140845,-0.000858478873239435
0.48284777620912,0.168226838565539,-9.86833718095473,17.7927092228286,1.8061216261768,20.237943617103,-0.000311419014084477,0.000568066901408454,-0.000325478873239443
0.487626011760158,0.173027302142447,-9.88731959900674,18.5261545145209,3.75564667856067,21.2213655987205,-0.000178419014084469,0.00016906690140845,0.000340521126760557
0.482911542704985,0.182683290062498,-9.83034413157207,18.5261545145209,3.75564667856067,21.2213655987205,-0.000710419014084473,-0.000230933098591547,-0.00112447887323944
0.487678760701408,0.173047089339488,-9.85408248121501,19.3583426414532,1.85140739167376,19.3020424463167,0.000221580985915529,-0.000762933098591552,0.000340521126760557
0.487692670276185,0.168242133059748,-9.84458941310869,17.9812138315203,3.6768351425676,22.4815563850894,-4.54190140844746e-05,-0.000629933098591551,0.000872521126760561
0.478051576612644,0.182662341746109,-9.86358878537644,18.8057876739718,1.14694727304698,21.9529010571255,-0.000444419014084471,-0.00049693309859155,-0.000325478873239443
0.48287618778525,0.192291380433009,-9.85407871017358,17.6134311364783,1.66881264065054,20.7703778556502,-0.000710419014084473,-0.00129593309859155,-0.000591478873239432
0.492508741632195,0.177868344593103,-9.83982778245861,17.6134311364783,1.66881264065054,20.7703778556502,-0.000710419014084473,-0.000230933098591547,0.000739521126760567
0.48285531064815,0.168229664886336,-9.86358972950677,19.080968035049,1.89664589425291,23.0584967940957,0.000221580985915529,0.000435066901408453,-0.000192478873239435
0.482846615089773,0.163415224331792,-9.86834000786582,18.4515449145707,2.14392643807985,20.5946626879239,0.000354580985915523,0.000435066901408453,-5.94788732394408e-05
0.48289531113397,0.177866022596864,-9.83984285291027,18.3011442179464,1.63294686997182,20.7477577156916,-0.000977419014084477,3.60669014084491e-05,-0.000192478873239435
0.492497722503971,0.163431679702892,-9.84458470558075,17.9033037327274,2.71105557577959,20.8224150253587,-0.000178419014084469,-0.00049693309859155,-0.000192478873239435
0.46845726946314,0.168234661913726,-9.8493679985648,17.9033037327274,2.71105557577959,20.8224150253587,-0.000977419014084477,0.00083506690140845,-0.000591478873239432
0.487654993360211,0.168227999684886,-9.86832964494209,17.3801614826331,2.59167964261215,21.366155399644,-0.000577419014084479,-0.00049693309859155,-5.94788732394408e-05
0.473272022626869,0.168238649944163,-9.84461201957299,17.3839680998085,2.90451254706383,20.6708251333185,-0.000444419014084471,0.000302066901408451,-0.000192478873239435
0.482901684453653,0.173057234683914,-9.83509822837341,18.214468111882,2.75396871182992,21.8857751617816,-0.000710419014084473,-0.000896933098591547,-0.000325478873239443
0.497273636301226,0.158610924256547,-9.86357277627435,17.1769513785941,0.428416565364737,20.1761012892645,-0.00150941901408447,0.000302066901408451,-0.000591478873239432
0.482886614001985,0.173051581452028,-9.84459412280053,17.1769513785941,0.428416565364737,20.1761012892645,-0.00124341901408448,-0.00049693309859155,-0.000991478873239443
0.473280719516399,0.173052086366879,-9.83986075027303,18.0248124756218,3.59570654364903,21.0486607336511,-0.00137641901408447,0.000968066901408451,0.000207521126760563
0.478086932863533,0.17305324724377,-9.839853215834,17.3730683989507,2.92479477091379,21.0290503552932,0.00102058098591552,-0.000363933098591548,0.000340521126760557
0.473243041026817,0.173037952401724,-9.86360197363765,17.4668375381719,1.47065879313595,20.2281380128503,-0.00111041901408447,3.60669014084491e-05,0.000739521126760567
0.492479760274598,0.182668651772784,-9.85881773593297,18.1076815921723,2.16185967709813,20.6059729809645,-0.000977419014084477,-0.00182893309859155,0.000606521126760559
0.478079396850894,0.173050420332681,-9.84460165881317,18.1076815921723,2.16185967709813,20.6059729809645,-0.000577419014084479,0.000435066901408453,0.000872521126760561
0.482910381585638,0.177871675828751,-9.83034695848316,18.6161165872901,1.98868492089315,21.1157870795532,-0.000844419014084469,0.000568066901408454,-0.000591478873239432
0.473272022626869,0.168238649944163,-9.84461201957299,17.7278323910161,2.88657925751999,20.659514808412,-0.00137641901408447,-0.00129593309859155,-0.000591478873239432
0.482880239108694,0.177860368774685,-9.84933973886861,17.5812533435073,2.68842371903637,20.1172754756193,-0.00124341901408448,0.000302066901408451,-0.00072447887323944
0.49729335231898,0.177861025569474,-9.85406458385225,17.1082675254017,1.19604923131138,21.293003221269,0.000354580985915523,-9.79330985915461e-05,-0.000325478873239443
0.482884292005747,0.163429357706654,-9.84459977603242,17.1082675254017,1.19604923131138,21.293003221269,-0.00190941901408448,0.000701066901408448,-0.000458478873239437
0.487681082940102,0.182670317806982,-9.85407682739283,19.0776826000123,2.5425776338222,22.0260532036345,-0.00124341901408448,-0.00262793309859155,-0.000192478873239435
0.473266808610469,0.177858046778447,-9.84935480932027,17.3621688255565,2.94507675757873,21.3872713880995,0.000886580985915528,0.000568066901408454,-0.00112447887323944
0.482903415354468,0.149003999870509,-9.83036391876911,17.2913662151203,1.64618154179071,20.0652387838994,-0.000710419014084473,0.00136706690140845,7.45211267605683e-05
0.482851259324705,0.18266067654466,-9.86832870081175,18.2329815150269,3.35934423923569,20.1368868382017,-0.00217541901408447,-0.00249393309859155,-0.000591478873239432
0.478073021957603,0.177859207655338,-9.84934727488124,18.2329815150269,3.35934423923569,20.1368868382017,0.00128658098591553,0.00123406690140845,-0.000991478873239443
0.502128545450102,0.173061878676391,-9.83506808747008,18.0646040983435,3.20175297933392,20.3110958229083,-4.54190140844746e-05,3.60669014084491e-05,-0.000325478873239443
0.478095628421909,0.177867687798314,-9.83510293747496,18.3790543167393,2.59872643675983,22.4068990754223,-0.00350741901408447,-0.00142893309859155,-0.000458478873239437
0.487700206288823,0.168244959970837,-9.83984097012952,18.8744715271641,0.379314607100342,20.835999125121,-0.000178419014084469,0.00123406690140845,-0.000458478873239437
0.492476277159012,0.168234813793664,-9.85882621607594,17.3474624119256,2.65252625486577,22.440830018276,-0.000311419014084477,3.60669014084491e-05,0.000340521126760557
0.47329114597559,0.153813292108018,-9.83037616230969,17.3474624119256,2.65252625486577,22.440830018276,-0.00164341901408448,3.60669014084491e-05,0.000207521126760563
0.478069538842018,0.163425369676218,-9.84935575502422,19.8738856390068,1.34512586738023,20.1489658022364,0.00155258098591553,-0.000230933098591547,7.45211267605683e-05
0.492518006972039,0.153817936100495,-9.83034602140635,17.0362981602346,2.60961288163043,21.3774656926846,-0.000444419014084471,3.60669014084491e-05,0.000340521126760557
```

```
0.482872704669664,0.177857542453888,-9.85408719031656,18.0319051550997,3.26259935440253,21.3857662523719,-0.00257541901408448,-0.00129593309859155,-0.000591478873239432
0.482858793763735,0.182663502865456,-9.8635812493638,18.3442216612496,0.593054560117936,21.0426349330441,-0.00031141901408477,0.00030206690140851,-5.94788732394408e-05
0.492525541411068,0.153820762421291,-9.8255985699584,18.3442216612496,0.593054560117936,21.0426349330441,-0.000178419014084469,-0.000363933098591548,0.000207521126760563
0.478055629509697,0.168231330678078,-9.85884882254025,18.8842044414252,3.07149130865372,21.884270026054,-0.00031141901408477,-0.0010299330985915,0.000207521126760563
0.487693831153076,0.173052742571375,-9.84458658678789,19.0956748528843,2.18918845791075,22.0049376895491,-0.00111041901408447,0.0011010669014085,-0.00072447887323944
0.487646296228226,0.163413558540049,-9.87308091483234,17.2046900134582,2.76720356153757,21.2032593718658,-0.00044441901408447,-0.000629933098591551,-5.94788732394408e-05
0.487678760701408,0.173047089339488,-9.85408248121501,18.0929748699346,1.86931693555153,21.6595289436347,-0.00150941901408447,3.60669014084491e-05,-0.000991478873239443
0.482884292005747,0.163429357706654,-9.84459977603242,18.0929748699346,1.86931693555153,21.6595289436347,-4.54190140844746e-05,-0.000363933098591548,7.45211267605683e-05
0.487678760701408,0.173047089339488,-9.85408248121501,18.1327667507375,1.47535660245195,20.9219667596945,0.000221580985915529,0.000435066901408453,-5.94788732394408e-05
0.492468741146374,0.168231986882574,-9.86357465905511,18.1495912482036,3.83442640246937,22.3073474003827,-0.000844419014084469,-0.000762933098591552,0.000739521126760567
0.482891828018385,0.163432184617744,-9.83985133305325,18.4226529608409,2.51759783784126,20.9740034239841,-0.000444419014084471,-0.0010299330985915,-0.00112447887323944
0.497274797178118,0.163421533768174,-9.86356994995356,18.9419882214212,2.3241487463159,21.1255927431021,-0.000844419014084469,0.00030206690140851,-0.000325478873239443
0.468435823875726,0.173036791282377,-9.86360950965028,18.9419882214212,2.3241487463159,21.1255927431021,-0.000977419014084477,-0.0011629330985915,-0.000192478873239435
0.492439759546321,0.173031289340136,-9.88256461311976,16.9257035228722,1.70467916921265,20.7929984735974,-0.000977419014084477,0.00083506690140845,-0.000192478873239435
0.473249416162563,0.168230169801187,-9.85885635697928,18.8095947909494,1.45977206055478,21.2575671745536,0.00115358098591553,-9.79330985915461e-05,-0.000325478873239443
0.473251738158802,0.177852393546561,-9.85885070374739,17.1180138755279,3.88822546269192,22.3412778652478,-0.00137641901408447,-0.000762933098591552,-0.000458478873239437
0.482850098205359,0.177849062310913,-9.86833152772284,18.1365735140362,1.78818833663296,20.2266332921964,-0.000977419014084477,0.00016906690140845,0.000207521126760563
0.468489733936323,0.177868192713165,-9.83036956494746,18.1365735140362,1.78818833663296,20.2266332921964,0.000886580985915528,0.00030206690140851,-0.000192478873239435
0.482900523576761,0.168246625172287,-9.83510105469421,18.2291748022537,3.04651151267278,20.8322202464035,-0.00177641901408447,-0.000762933098591552,0.00100652112676056
0.53574575611959 6,0.168248090051509,-9.85400995370386,18.2689664249754,2.65255794835765,20.0946553356607,0.00315058098591553,-0.0389799330985916,-0.000325478873239443
0.381847010607078,0.15374883158028,-9.90174102413958,18.9201889471692,2.3647129568308,21.8420389978831,-0.0105644190140845,0.0230720669014085,0.00406852112676057
0.622258599757243,0.0912910109364672,-9.84917209109604,18.5621394244695,3.04886038768265,21.1791340961796,0.0135375809859155,0.0453100669014085,0.015920521126 7606
0.44924857402904,0.278886280957216,-9.85408057825698,16.7137119106147,1.62821830902029,22.400103678893,-0.0336014190140845,-0.0618839330985915,-0.0591824788732394
0.353079339813189,0.177823367502531,-9.85903909147448,16.7137119106147,1.62821830902029,22.400103678893,0.00115358098591553,-0.0317899330985916,-0.130289478873239
0.429991526111671,0.1537887093864,-9.85418421353601,18.4444523306905,2.4770334494376,20.2575540273267,0.0103415809859155,0.00909006690140845,-0.237749478873239
0.430066333049715,0.245222133379499,-9.82089438009684,18.4444523306905,2.4770334494376,20.2575540273267,-0.0165564190140845,0.0104220669014085,-0.279695478873239
0.492560372324466,0.298158137490378,-9.82551376911893,18.8662117843486,3.4248884236203,21.9053860145095,-0.0126954190140845,0.0132180669014085,-0.288217478873239
0.439660663329239,0.32219112613022 6,-9.8445743138526,17.0003132502861,3.31639917250847,21.4196971952256,-0.00550441901408447,0.0302630669014084,-0.284222478873239
0.43481922924774,0.274068356696794,-9.85885445461154,17.0003132502861,3.31639917250847,21.4196971952256,-0.000710419014084473,0.0194770669014084,-0.301933478873239
0.420363637813614,0.249997505045104,-9.87788397326255,16.3768299780866,5.94303805635583,20.6994719988123,-0.0152254190140845,-0.00875293309859155,-0.312319478873239
0.381955466267673,0.322168713524909,-9.85890907711611,18.9195578748492,6.03593576837171,21.5207607860157,-0.0355984190140845,0.0232050669014085,-0.362787478873239
0.343523369822689,0.384708213636699,-9.85418417062452,15.4667322840295,6.88148960021462,20.959649127947,-0.0513114190140845,-0.00968493309859155,-0.355330478873239
0.367570787521081,0.408772906793814,-9.84938490888573,16.1615524496498,6.51251611959101,21.2741382178891,-0.0553064190140845,-0.0393799330985916,-0.371442478873239
0.382036101205106,0.500198507438726,-9.83506425783649,16.1615524496498,6.51251611959101,21.2741382178891,-0.0587684190140845,-0.0214029330985916,-0.371176478873239
0.319578622413414,0.567547647715356,-9.82562674931155,15.4454541600504,7.88081851404674,19.9483225732335,-0.0521104190140845,-0.0261969330985916,-0.382228478873239
0.204190310748249,0.721456768087296,-9.86370171619432,16.3484593020932,7.27547392224946,19.3510357362095,-0.0614314190140845,0.0102890669014085,-0.412189478873239
0.13702419065831 7,0.885057261482574,-9.80673375498455,15.5560628484082,8.78575865243936,20.5327939185744,-0.00577141901408447,0.0256020669014085,-0.441484478873239
0.233001419368414,0.596371811462247,-9.86372999705111,14.2590379333186,10.7820359708981,20.5094026892787,0.0424325809859155,0.0431790669014084,-0.413787478873239
0.309958841483072,0.634894421828291,-9.83984559183449,14.2590379333186,10.7820359708981,20.5094026892787,-0.0423904190140845,0.0256020669014085,-0.440952478873239
0.175463823115939,0.822520588281873,-9.80671021849697,14.9500364498234,10.1002384606501,21.5192261397812,-0.0437214190140845,-0.00941893309859155,-0.428435478873239
0.117664538452217,0.74067849552831,-9.86857349780044,13.6002022415765,10.4442311589951,20.1526836184578,-0.0679564190140845,-0.0465699330985916,-0.478636478873239
-0.026439857516087,0.913871328917623,-9.8307132320784,12.9440058476742,13.1317102291212,20.5071289144229,-0.0656934190140845,-0.0657459330985916,-0.480766478873239
-0.25238285319961 8,0.952295353079611,-9.85003756478866,12.0590075411669,13.3836638947418,21.0832997000766,-0.0538414190140845,-0.0781289330985916,-0.457597478873239
-0.300349954087763,1.06297109955274,-9.80256645076348,12.0590075411669,13.3836638947418,21.0832997000766,-0.0519774190140845,0.0105550669014084,-0.401803478873239
-0.170623863303911,0.971574676241506,-9.82615792312799,11.86173679774,13.5997520241821,21.6368499270793,0.00661258098591553,0.0313280669014084,-0.323238478873239
-0.136985642885556,0.933092903645952,-9.82612778927819,9.7554755261499,14.7472449033335,21.4098336074384,-0.0515784190140845,0.0200100669014085,-0.408594478873239
-0.184875157686908,1.23623046006551,-9.75955181664569,9.44050315680619,14.3915068524122,21.0418042435768,-0.0300064190140845,0.0132180669014085,-0.336155478873239
-0.338802927669847,1.10144888411786,-9.80735256360509,8.93535403279542,13.9187418706965,21.5644322137871,0.0228585809859155,-0.0243329330985916,-0.146668478873239
-0.377240828984199,1.13993332722728,-9.80264178989807,8.93535403279542,13.9187418706965,21.5644322137871,-0.0256114190140845,0.00336506690140845,-0.0551874788732394
-0.223463266031293,1.0581705865954 1,-9.81669304615639,9.74838294226972,15.0803519146912,21.0727249468412,-0.0313374190140845,0.0120200669014085,-0.0675714788732394
-0.213720527439003,1.18811319257432,-9.75487685393515,8.87428428609464,15.3120243488437,21.2906705698164,0.0200625809859155,-0.00662193309859155,0.00753052112676056
-0.329249960754857,1.03407667040875,-9.83586574019617,8.42309679209147,13.7790847680438,21.7499530805469,0.0221925809859155,-0.0199379330985915,0.0358945211267606
-0.314814653546343,1.06776227038495,-9.83107490512926,10.1031469343351,15.0421364012975,20.70318939996,-0.0334684190140845,-0.00249393309859155,0.0549355211267606
-0.237795765618102,1.17365941610851,-9.7786632428275,10.1031469343351,15.0421364012975,20.70318939996,-0.0202854190140845,0.00136706690140845,0.0962155211267606
-0.343547080718301,1.20249872305997,-9.78355951367867,9.2574180809419,13.9413728421931,22.2695681755275,0.00394958098591552,0.00456306690140845,0.151210521126761
-0.199342200698138,1.16885809482429,-9.77860578576802,7.843213709543,16.3245876407,19.5968244547109,0.0929005809859155,-0.0316569330985915,0.358940521126761
-0.170591968612196,1.0100730560881,-9.81189097220387,9.4546886969051,13.7252849499378,21.7160221376932,0.0620075809859155,-0.0241999330985916,0.548826521126761
-0.338815675882821,1.11106645935347,-9.81684280421003,11.0525008267843,12.7509605789053,21.4332233743682,0.00341758098591553,-0.0191399330985915,0.651359521126761
-0.27636124594176 9,1.05814933510944,-9.83102027340726,11.0525008267843,12.7509605789053,21.4332233743682,0.0324455809859155,0.0337250669014085,0.776530521126761
-0.0841173469683612,1.02450800615398,-9.84498297953558,13.0159752633668,11.7181455864203,20.4226663837111,0.0496235809859155,-0.0119489330985915,0.776663521126761
-0.11299750318987 7,0.957134984117977,-9.85931210367426,14.4198024890604,10.3139696662036,21.7258609634798,0.0319135809859155,-0.0729359330985916,0.668138521126761
-0.141897396985558,0.807970548440997,-9.87368928175971,15.7265738817397,11.0098683419404,22.7975226475859,0.0307145809859155,-0.0348519330985915,0.649096521126761
-0.108137949563614,0.990832395472809,-9.83079610722563,16.2579759515876,8.08367121378478,21.1843912540398,0.0404355809859155,-0.107290933098592,0.608482521126761
-0.0602057011452784,0.673277961602539,-9.86889188361493,16.2579759515876,8.08367121378478,21.1843912540398,0.0956965809859155,-0.101298933098592,0.793175521126761
0.103269343269784,0.596360250861476,-9.83069633586453,17.1140820330776,8.20539484292074,20.9875605129318,0.0193965809859155,0.0478400669014084,0.677725521126761
0.3676850482935,0.601248850462937,-9.8065388284874,17.6127855320202,5.34003621007485,20.4491001217714,0.0128715809859155,0.00988906690140845,0.640041521126761
0.156079789100742,0.577104956473765,-9.86860932231369,17.8242414113415,4.45773411721528,20.569768263255,-0.00111041901408447,-0.0690749330985916,0.640574521126761
-0.012193230013132,0.567430789406649,-9.88787149749006,18.6346301178986,2.5940602111139,19.3668945668048,0.00275158098591553,-0.0496329330985915,0.626991521126761
0.141700848134433,0.500143282746667,-9.83069258716428,18.6346301178986,2.5940602111139,19.3668945668048,0.0425665809859155,-0.0355179330985915,0.648563521126761
0.199360809379823,0.43760842686981,-9.83538735117892,17.8466867393281,0.745939212714075,20.1745961854028,0.0403025809859155,0.00775906690140845,0.634049521126761
0.232911472831096,0.317309800204352,-9.87813828349473,19.4695827052844,-0.914881235765858,20.2077916477474,0.0415005809859155,-0.0369829330985916,0.604487521126761
0.218467034710563,0.249945914515821,-9.882948906742,19.5993364007753,-3.07579655774851,19.3646482178373,0.0504225809859155,-0.00196193309859155,0.511408521126761
0.26171971389227 3,0.274006772945477,-9.89236185687012,18.7356166169982,-3.82317793814018,20.9521420605506,-0.0108314190140845,-0.0144789330985916,0.497293521126761
0.2523138817842,0.509813001626245,-9.79727749137978,18.7356166169982,-3.82317793814018,20.9521420605506,-0.0196194190140845,-0.0300589330985916,0.575991521126761
0.223350221492654,0.346192114452307,-9.84964771884789,19.6996767956147,-5.82180302063442,20.6286215939507,0.0196625809859155,-0.0368499330985915,0.664942521126761
0.122459051114142,0.442404291317373,-9.83075664962284,19.6849704775815,-6.11435370123613,21.6821770822509,0.0637385809859155,-0.0349859330985916,0.514205521126761
0.11753857699459,0.249927186771358,-9.8736102644602,17.9166476999155,-7.36414602567412,19.7002456541986,0.0654695809859155,0.0152160669014085,0.491168521126761
0.324213528520092,0.134509234344568,-9.86860563768553,18.4070910572916,-7.18392470981997,20.2311756500806,-0.0104314190140845,0.0351900669014085,0.477186521126761
0.396341852542602,0.148968967802135,-9.85423978938835,18.4070910572916,-7.18392470981997,20.2311756500806,-0.0322694190140845,0.0112210669014084,0.385306521126761
0.3482748573668 7,0.153768971812235,-9.8543123163092,17.6409470804366,-9.07261015591971,20.3224268247292,-0.0531754190140845,-0.0357849330985915,0.367329521126761
0.271335990393074,0.129685853247491,-9.86869135542452,16.3412673665601,-10.1016159110812,19.5878717385019,-0.0206844190140845,0.00123406690140845,0.378914521126761
0.329015097874749,0.115265947730712,-9.86860940971027,16.5129461552976,-10.589965197509,20.4461043127398,0.0434985809859155,0.0600910669014084,0.355078521126761
0.492464643805344,0.0575813774263314,-9.84939533588154,16.5129461552976,-10.589965197509,20.4461043127398,0.0428325809859155,0.0574280669014084,0.316462521126761
0.550200004657812,0.120155404691644,-9.82552793129822,16.5129461552976,-10.589965197509,20.4461043127398,0.0108745809859155,0.0261350669014084,0.234702521126761
0.487800510137726,0.240445242936962,-9.78757065429989,15.5931156425772,-12.3436905330924,19.6580073990651,0.0148685809859155,-0.0132799330985916,0.0840985211267606
0.343475767323841,0.187446096162756,-9.85430006571509,15.0377951183694,-11.4434482040158,19.5486499975617,0.0199285809859155,-0.0110169330985916,0.0228445211267606
0.36272799104578,0.22594729805531,-9.8447514147029,15.080872965877,-12.4833484529248,19.8435267405442,-0.0105644190140845,-0.0163429330985915,0.114325521126761
0.377116743760924,0.187451396602184,-9.85899575975301,15.2672436042416,-12.67914641946,19.6482022098862,0.00248458098591553,-0.0144789330985916,0.0721135211267606
0.357917267649083,0.148970986038499,-9.83530828391439,15.2672436042416,-12.67914641946,19.6482022098862,0.0253885809859155,0.00669406690140845,-0.00964647887323944
0.405962638850511,0.0816221945219484,-9.84001995376057,15.2672436042416,-12.67914641946,19.6482022098862,-0.0102984190140845,0.0102890669014085,-0.0479964788732394
0.357905089217577,0.129723712226955,-9.84006704182611,15.8644883671442,-11.9068237734307,21.4589334935229,-0.0122954190140845,-0.00449193309859155,-0.0329494788732394
0.362707092352268,0.139344270180586,-9.84480229556076,14.7228241033797,-11.7991933114321,19.1806191394684,0.00461558098591552,0.00988906690140845,-0.110448478873239
0.386686055118342,0.00462644382572075,-9.85908810365864,15.0268954493775,-11.423166039462,19.9068741722443,0.0269865809859155,0.00389706690140845,-0.241478478873239
0.454008165793719,0.0190850164063968,-9.84472978980049,15.0268954493775,-11.423166039462,19.9068741722443,0.0215265809859155,0.0166810669014084,-0.460127478873239
0.473202974783063,-0.0242203334390088,-9.85896942515876,15.6857320780629,-11.0853612187883,20.2635942584915,0.0192635809859155,0.00642706690140845,-0.540689478873239
0.232825600747242,0.086358090206476,-9.8972667455775,15.9614182159294,-9.37689732304121,19.6414135066532,-0.0527764190140845,-0.0468369330985916,-0.455866478873239
0.290538333821447,0.0863833297228824,-9.87818353933483,17.6158618713458,-8.38610608889163,20.0064331052954,0.0188635809859155,0.00123406690140845,-0.546947478873239
0.415479815492122,-0.00500577397443402,-9.88753721877448,18.9951242155355,-8.14501435850806,19.2658579868292,0.0496235809859155,0.0113540669014085,-0.570516478873239
0.367490676347531,0.0767966427182783,-9.84957995099359,18.9951242155355,-8.14501435850806,19.2658579868292,0.0169995809859155,-0.0228679330985916,-0.685434478873239
0.271294214562462,0.0190211098260937,-9.87825325561557,17.9699934367371,-4.75309173424948,19.3156208407785,-0.00896641901408447,-0.0409779330985916,-0.622316478873239
0.223249257400721,0.0526934388944934,-9.86881193688243,19.4555229923844,-4.87865559561369,21.5826237907685,0.0422995809859155,0.00150006690140845,-0.619519478873239
0.314642553358143,0.0960367990090073,-9.83540719696812,18.1840895508415,-2.61010993630916,20.1474543644744,0.00861058098591553,-0.00129593309859155,-0.608600478873239
0.213668882713558,0.096001110978593,-9.85455722949889,19.6952387524402,-2.46340616687195,21.0026740634826,-0.0138934190140845,-0.0387139330985916,-0.603940478873239
0.242496836887727,0.0719491885741797,-9.85927459292431,19.6952387524402,-2.46340616687195,21.0026740634826,0.0279185809859155,-0.0352519330985915,-0.450939478873239
0.223280582310968,0.120056667788236,-9.85927646865153,18.4908157088325,0.791201283070533,21.5848712188939,0.00634658098591553,-0.00289393309859155,-0.507399478873239
0.237696608855372,0.163356011609297,-9.86872431618541,19.1283737961282,2.12834208284214,20.9302672600855,0.0264535809859155,0.0632870669014085,-0.463722478873239
0.271397434650245,0.134519076276956,-9.83070395986404,19.4973224045554,1.42390553199267,21.2349465566065,-0.0349334190140845,-0.00529093309859155,-0.482764478873239
0.199194917915134,0.124842690303411,-9.89254744491693,18.6199233000632,2.30151764745607,20.4204536713513,-0.00403941901408447,-0.0321889330985915,-0.321907478873239
0.122329696760035,0.18738138149802,-9.87363947750416,18.6199233000632,2.30151764745607,20.4204536713513,-0.0220164190140845,0.00416406690140845,-0.361988478873239
0.184836305860869,0.163348893498189,-9.85931131160288,18.5735600175189,3.98734963593438,19.0931333379078,-0.0124294190140845,-0.0522959330985915,-0.170237478873239
0.107919498457476,0.230679412319797,-9.87363664353954,18.1392127800101,4.81347227795841,20.9377987062746,-0.00390641901408448,-0.00821993309859155,-0.149997478873239
0.131949936416496,0.211440770183328,-9.87361027151373,19.1713256088813,5.7184438726579,19.1760911798317,0.00634658098591553,0.0104220669014085,-0.0541224788732394
0.184826426295581,0.0911825305136754,-9.85460526933859,18.544668095655,4.36102097639955,19.4724730266758,0.00794458098591553,0.0272000669014084,0.0176515211267606
0.194464215715338,0.129681410167535,-9.84507169035168,18.544668095655,4.36102097639955,19.4724730266758,0.0160675809859155,0.0289310669014084,0.0188495211267606
0.199251148398361,0.13929631488928,-9.85930283851343,19.127726869182,5.79957264946523,20.6089899731463,0.00448258098591553,0.00190006690140845,0.0233775211267606
0.218440900686695,0.11042197586945,-9.8782824382815,17.6307777848923,4.98664703416339,20.4279846076859,0.00940958098591553,0.0302630669014084,0.0349615211267606
0.256901844303907,0.100811867505031,-9.87347936358033,18.2008038044474,4.37895426594338,19.4837833515824,0.0327125809859155,0.0493050669014084,0.0172515211267606
0.372256710337042,-0.000191480434700983,-9.8638619800132,18.5865931570357,6.03358707125059,21.1738500781159,0.0224585809859155,0.0434460669014085,0.00819652112676056
0.425194120951871,0.0383309778089495,-9.83051935885905,18.5865931570357,6.03358707125059,21.1738500781159,0.00315058098591553,0.0125530669014084,0.0141885211267606
0.290547030710977,0.0911967661455986,-9.87343227003487,18.8018561533346,5.46410976589882,20.5991843370277,0.00687958098591553,0.000568066901408454,-0.0143074788732394
0.353067115364098,0.0334944725764204,-9.84487955490766,17.9675495240856,5.30182069668106,20.0795645748901,0.00781158098591553,-0.00981793309859155,-0.00911447887323943
0.309744730146874,0.0286497951302425,-9.88293576040896,19.1386265381739,5.77929048491152,20.2507657984637,-0.00630341901408447,-0.00728793309859155,-0.00179047887323944
0.338613277718351,0.0479106943075491,-9.86863391148878,18.2716208195579,5.67784896103301,20.8058196669623,-0.00497241901408448,0.00669406690140845,0.00100652112676056
0.362683871662518,0.043119018560488,-9.8448588296505,18.2716208195579,5.67784896103301,20.8058196669623,0.00528158098591552,-0.00116293309859155,0.00127252112676056
0.333803738328567,0.0382863047207082,-9.8686471013236,17.963742407108,4.98899590917325,20.774898457462,0.00315058098591553,-0.00276093309859155,-0.000591478873239432
0.338635884182657,0.0479191744505249,-9.8543895740825,18.1141275709193,5.4999763449865,20.6218049868408,-0.00430641901408448,0.00256606690140845,0.000207521126760563
0.338649793514978,0.0431132134486643,-9.84489650656647,17.8171488274614,4.79084112857302,20.2326596026579,-0.000710419014084473,-0.00142893309859155,-0.000192478873239435
0.32895478979084,0.0527020207042378,-9.89713582353704,17.178959218569,7.12493026490432,20.565988906549,0.00181958098591553,-0.000496933098591551,-5.94788732394408e-05
0.357885351643412,0.0479322985859775,-9.84011509577288,17.178959218569,7.12493026490432,20.565988906549,-0.00177641901408447,0.00136706690140845,-0.000325478873239443
```

```
0.343440936410444,0.0431087210936694,-9.85438486655456,17.5370086456711,6.44078301194123,21.2288969501289,-0.00137641901408447,-0.00142893309859155,0.00100652112676056
0.328997682296823,0.0430967578351083,-9.86865181042515,17.6307777848923,4.98664703416339,20.4279846076859,0.000753580985915533,-9.79330985915461e-05,0.000340521126760557
0.343431078401567,0.0334836704372062,-9.85913896276561,17.9670283419469,4.34305605266009,21.8073384316767,-0.000444419014084471,0.000568066901408454,-0.000591478873239432
0.338565740977437,0.0382705049638109,-9.89712923165474,18.4051816320263,3.82975842655816,19.2673423544803,-0.000977419014084477,-0.00156193309859155,-5.94788732394408e-05
0.343438614414206,0.0334864973482957,-9.85439051978645,18.4051816320263,3.82975842655816,19.2673423544803,-0.000977419014084477,3.60669014084491e-05,-0.000192478873239435
0.338649793514978,0.0431132134486643,-9.84489650656647,15.5496017223929,5.44763584628674,20.5169620074788,0.000354580985915523,0.000701066901408448,0.000207521126760563
0.343471077313779,0.0431200275574421,-9.83539307770032,17.5762794717988,5.08805779763566,22.2191044338068,-0.000311419014084477,-0.000363933098591548,-0.000325478873239443
0.343447309730126,0.0382999331807193,-9.8496402420177,18.8378410632831,4.75732347502078,20.5569528344868,-0.000577419014084479,-0.000629933098591551,0.000872521126760561
0.343427025504514,0.0479146815052374,-9.8638789256018,17.4226073724736,5.22301732815741,21.3397590093713,-0.000710419014084473,-0.00129593309859155,-5.94788732394408e-05
0.367566622848511,0.110500368617074,-9.80682715216799,17.4226073724736,5.22301732815741,21.3397590093713,0.000620580985915525,-0.00355993309859155,0.000872521126760561
0.348293364916538,0.0431268419086754,-9.82588964726057,17.0934498672355,5.53349324065534,20.2975112770233,-0.00497241901408448,-0.0151449330985916,0.0341635211267606
0.165457618473833,-0.149415834661548,-9.90700664492192,17.3016203115201,5.29712288736506,19.3857358280459,0.0176655809859155,-0.134056933098592,0.0512075211267606
0.242567546484742,0.0527320031110364,-9.81180543631157,17.6089784150426,5.02721142256705,21.1444340043432,0.0120725809859155,-0.141379933098592,-0.0332154788732394
-0.0266505743821977,0.0718983038722681,-9.83595634790008,17.6312990626287,5.94541150029559,18.700207609023,0.0319135809859155,-0.229531933098592,-0.0333494788732394
-0.233394595307918,-0.0676840394121638,-9.84585824178518,17.6312990626287,5.94541150029559,18.700207609023,0.0323125809859155,-0.262688933098592,0.00273752112676057
-0.560282145850612,-0.091824704665204,-9.85588068067995,18.6045854099077,5.68019789533913,21.1527345640305,0.0260545809859155,-0.283195933098592,0.0626595211267606
-0.81978673412979,-0.130349033457175,-9.80882974531489,20.3603965838745,5.84251048180865,19.3261749800143,0.0155345809859155,-0.294647933098592,0.0797035211267606
-1.05527301734878,-0.0822796665268318,-9.78542949149486,20.1527474997179,7.037644249553,18.5101723237404,0.0233915809859155,-0.277735933098592,0.146017521126761
-1.39654527109924,-0.0823564472974828,-9.77646960615606,21.3282795584049,4.15670814884636,18.3073158725234,0.00621358098591553,-0.282130933098592,0.153341521126761
-1.77612846004902,0.0138279635408162,-9.69154234856599,21.3282795584049,4.15670814884636,18.3073158725234,-0.0106974190140845,-0.289853933098592,0.150012521126761
-2.06449057519785,-0.0295205989479584,-9.65403534746775,22.5178703611829,5.2395464585131,16.7296273101511,-0.00177641901408447,-0.313955933098592,0.162529521126761
-2.37202837441369,-0.0439891688371796,-9.58805186221672,23.6039607065536,5.08433563974988,14.9045723520081,-0.0181544190140845,-0.373078933098592,0.114458521126761
-2.70353432350511,-0.0391954790456484,-9.48411215907673,25.0737072744604,4.51723020596134,12.3306448257221,-0.0140264190140845,-0.370947933098592,0.0613275211267606
-3.03983164485037,0.0666300847853006,-9.38486709302919,25.0737072744604,4.51723020596134,12.3306448257221,-0.0313374190140845,-0.395049933098592,0.0537375211267606
-3.52016476494513,0.15324138409698 5,-9.17665413020137,25.0737072744604,4.51723020596134,12.3306448257221,-0.0686224190140845,-0.450311933098592,-0.0141744788732394
-4.09192740396203,0.148387715085452,-9.01611752365018,25.4720553466236,4.39788691082863,10.5282151526832,-0.00910041901408447,-0.432734933098592,0.0209805211267606
-4.41854019539108,0.138782648936949,-8.85520042505779,27.3815671843892,4.42524656795348,9.58100365337185,-0.0174894190140845,-0.453107933098592,-0.0430694788732394
-4.77400201989076,-0.000725444869378321,-8.68016052170091,27.769014714349,4.32618531001127,8.13679504500516,-0.0465174190140845,-0.516625933098592,-0.0246934788732394
-5.33104077456818,0.220647443059007,-8.36278272490219,28.7940357369538,5.56426422132046,6.03798510985195,-0.0328024190140845,-0.454039933098592,-0.0167044788732394
-5.43652543477523,0.292884371841356,-8.20621839240737,28.7940357369538,5.56426422132046,6.03798510985195,0.00488158098591553,-0.390389933098592,0.0460145211267606
-5.72942594907475,0.297742879227917,-8.00725648761258,28.2926928567092,5.40432389974937,5.86527608747994,0.0239235809859155,-0.358963933098592,0.0711815211267606
-5.99353834764971,0.273724722261706,-7.83675302290859,30.8518004948963,4.03607789507859,2.4987419199653,-0.00963241901408447,-0.339921933098592,0.0525395211267606
-6.3632467096685,0.297846853237146,-7.57617359525839,29.7302610999769,5.85683772058679,2.63825084470151,-0.0530424190140845,-0.306897933098592,-0.00392047887323943
-6.61282845605175,0.350845899017817,-7.34862593738688,30.5155500839649,4.67966770631125,1.11938489480188,-0.0122954190140845,-0.297443933098592,0.0247085211267606
-6.85279602328351,0.442335344066916,-7.12578903950835,30.5155500839649,4.67966770631125,1.11938489480188,0.00461558098591552,-0.194643933098592,0.0456145211267606
-6.89116902035513,0.437545535266574,-7.07362324315376,29.7281281424604,3.79031087756238,0.199304278276382,-0.0178884190140845,-0.0815919330985916,0.0203145211267606
-6.97759669816954,0.399074310673237,-7.00730825140518,29.4915869771103,5.35911628225314,-0.237353452768833,-0.0241474190140845,-0.0469699330985916,0.00153852112676056
-7.02558601594348,0.413524805768727,-6.95989366989614,30.1411970834543,3.96351657206233,-2.65668037305536,-0.0234814190140845,-0.0966389330985916,-0.0155054788732394
-7.04473966490126,0.471278126968115,-6.92190532256149,31.257776169994,4.54239011779561,-0.694360785726741,-0.0301394190140845,-0.228333933098592,-0.00645047887323943
-7.21748328532933,0.447295157877212,-6.72751944334713,31.257776169994,4.54239011779561,-0.694360785726741,-0.0178884190140845,-0.268947933098592,-0.0192344788732394
-7.38550474131568,0.418472651803172,-6.58535677053899,30.334675723184,3.43459571337259,-2.51490179971705,0.00488158098591553,-0.207427933098592,-0.0274904788732394
-7.42858769787781,0.500311972618 56,-6.48566617315785,30.1270119794258,4.62973047618533,-3.33089978883388,-0.00337441901408447,-0.120074933098592,-0.00884747887323943
-7.39492976277286,0.481081305467761,-6.47612883782686,31.7428152340383,3.30201727624817,-3.63480879791789,0.0296495809859155,-0.0851869330985915,0.0151215211267606
-7.53415281620901,0.461870023771901,-6.3624049657923,30.9651255981887,5.10484457009591,-3.50660972009964,0.0119395809859155,-0.151500933098592,0.0117925211267606
-7.78849891012494,0.442722337367872,-6.09692168328712,30.9651255981887,5.10484457009591,-3.50660972009964,-0.0358654190140845,-0.142844933098592,-0.0278894788732394
-7.83646621434606,0.423504848735146,-6.03053510012089,29.4681753188745,4.29192571480785,-3.68761882778886,-0.0289404190140845,-0.0921109330985916,-0.0316184788732394
-7.73562237580188,0.447545718222493,-6.09683596712174,29.9193627172799,5.82486547349658,-4.14689819664301,0.00474858098591553,-0.0276619330985916,0.0164525211267606
-7.65392421775397,0.558207584519632,-6.1251325164596,29.6332827058705,5.09544808375872,-4.89426877093474,0.0168665809859155,-0.0308579330985915,0.0453485211267606
-7.75001459031865,0.534148089302428,-6.09206024128024,29.6185758880351,4.80290552010088,-3.84070966638817,0.00288458098591553,-0.0357849330985915,0.00340352112676057
-7.75963557814347,0.47160263278917,-6.08736361564544,29.5046815384254,4.54390383076576,-5.45762837628488,0.000354580985915523,0.0447770669014085,-0.0149734788732394
-7.74528914791039,0.413848149880142,-6.12536049052393,29.5046815384254,4.54390383076576,-5.45762837628488,0.0329785809859155,0.0819290669014084,-0.00685047887323943
-7.69734852460704,0.384946857109872,-6.20127123545731,31.2092952078189,4.16169486991432,-4.46062086440506,0.0135375809859155,0.154768066901408,-0.0143074788732394
-7.62046700479184,0.514846357379273,-6.23905891566633,30.8545312157535,4.19991038330811,-4.09108531752381,-0.0349334190140845,0.288194066901408,-0.0337484788732394
-7.4094686551865,0.490654819061873,-6.55211492953865,31.0730798733043,2.98449462889882,-3.63330369405619,-0.0206844190140845,0.463033066901408,-0.0530564788732394
-7.06404830088299,0.350971377711885,-6.95524324931146,30.0256289280624,5.45820999157062,-1.13931904902886,0.0466945809859155,0.615635066901408,-0.0663724788732394
-6.69454282568788,0.297908076992802,-7.33928923939378,30.0256289280624,5.45820999157062,-1.13931904902886,0.0323125809859155,0.663839066901408,-0.00964647887323944
-5.9647323841408,0.345881091781731,-7.86990153552561,29.2218107313756,6.03001304801571,2.12843306679884,-0.0355984190140845,0.715372066901408,-0.0847484788732394
-5.38850900072988,0.355416503306641,-8.24883930232797,28.4049755265389,4.55556321219228,3.31547123228859,-0.00483841901408447,0.733481066901408,-0.0851484788732394
-4.64428264780358,0.133984306769154,-8.74160371009248,27.6719597358867,6.42626096641353,7.90526427135701,0.0211275809859155,0.789808066901408,-0.0634434788732394
-3.9430157235111,0.162817971535983,-9.08234868690375,27.2659986434525,5.91994699343123,11.0983664397642,-0.0391944190140845,0.760113066901408,-0.0566524788732394
-3.14541713581742,0.220613199582658,-9.30422483659818,27.2659986434525,5.91994699343123,11.0983664397642,-0.0237474190140845,0.628285066901408,-0.0839494788732394
-2.75632364466447,0.259095381796748,-9.47452290903548,24.6900509244607,4.92912505817166,13.0795215830335,-0.118291419014084,0.556245066901408,-0.146934478873239
-2.25176193218973,0.345752477920988,-9.58763451121958,23.5968680270757,5.41744282899639,14.5674668332873,-0.0573034190140845,0.571026066901408,-0.0515924788732394
-1.71844270700954,0.312114434527734,-9.7340086894689,21.4323013440742,6.35352075102666,16.826905210335,0.0506885809859155,0.532942066901408,0.0247085211267606
-1.12734081178767,0.158258068573278,-9.79964651723439,20.4688720916545,4.68092419225063,17.4717070899749,0.0605425809859155,0.487801066901408,0.0862285211267606
-0.863227339407705,-0.063075400685019,-9.93230759147657,20.4688720916545,4.68092419225063,17.4717070899749,0.0158005809859155,0.468892066901408,0.0275055211267606
-0.329461792722729,0.31237365224421,-9.86002997740748,19.8988461995631,5.28861672328564,18.41590415691,-0.0166904190140845,0.585141066901408,0.0457475211267606
0.655999470724369,0.197173058561049,-9.80632414359763,18.1969966556039,4.06612953773183,20.1791182814463,-0.0154914190140845,0.574089066901408,0.134698521126761
1.02614491289156,0.235755184819168,-9.80097285661492,17.6264498394549,3.71505065596139,22.8510919319706,-0.0141604190140845,0.523488066901408,-0.0621114788732394
1.36269984843706,0.389811194023509,-9.77661468857541,15.0870078588715,2.97621402051799,23.0622421309861,-0.00443941901408447,0.462234066901408,0.0264395211267606
1.89636026635753,0.173489033218185,-9.68094353582313,15.0870078588715,2.97621402051799,23.0622421309861,-0.0194864190140845,0.456375066901408,0.0819675211267606
2.30985340887624,0.130330773117701,-9.60910023028294,13.7115526316594,3.04794707764221,23.1074833668805,-0.0526434190140845,0.506976066901409,0.0646565211267606
2.95435941333196,0.23165789513412,-9.38012171963796,12.0064322166674,2.47139081447806,23.838252375675,-0.0671574190140845,0.543462066901408,0.0157865211267606
3.42087057851537,0.34251157899488,-9.24163182612958,11.1497904527666,1.39090323990827,25.7628564042661,-0.0494474190140845,0.521091066901408,0.0137895211267606
3.94997599950809,0.328338309474388,-9.01765144226294,9.3727011866866,2.22791950797464,26.5780883862767,-0.00763541901408447,0.490198066901408,0.0438835211267606
4.3348313293681,0.347789713216032,-8.82711246899802,9.3727011866866,2.22791950797464,26.5780883862767,0.0167335809859155,0.438798066901408,0.00406852112676057
4.70041763100648,0.352787495466055,-8.66035433383914,6.12044675574498,2.21144558836256,26.4958660567277,0.00381658098591553,0.425083066901408,0.0309675211267606
5.03727280259367,0.439604651010201,-8.43661501591546,6.63268846363592,2.3511035587205,26.3103467471145,-0.0196194190140845,0.476216066901408,0.0408215211267606
5.4702524732144,0.348439668446794,-8.19383778793655,3.36953436370243,2.35491180366212,26.5863485922481,-0.000178419014084469,0.453179066901408,0.0498755211267606
5.71556168248532,0.223476621298009,-8.07482482172083,2.25243412688997,0.817273554611627,26.351801814414,0.0102085809859155,0.488600066901408,0.0303015211267606
6.27879169142688,0.281653097961496,-7.56111509019806,2.25243412688997,0.817273554611627,26.351801814414,-0.0138934190140845,0.547057066901408,-0.000192478873239435
6.72635952004446,0.166499546299778,-7.2091231334489,0.202282261754969,2.97111738615351,28.5003766981008,-0.00683641901408447,0.465164066901408,0.0408215211267606
6.97198605711442,0.330305534751272,-6.93325384659966,-2.47993857714722,2.34695152368385,27.4739571059857,0.00834358098591553,0.473553066901408,-0.0429364788732394
7.21750994545168,0.349748708295044,-6.70020138127753,-2.26086071339719,2.09029860163539,26.2039572273984,0.00581358098591553,0.382604066901408,-0.0364114788732394
7.52573404124359,0.335609659156892,-6.32937680173012,-3.53544743584352,0.374788315945098,25.7853945583856,-0.00350741901408447,0.309766066901408,0.0189825211267606
7.66054226851233,0.263560606519296,-6.18201639278522,-3.53544743584352,0.374788315945098,25.7853945583856,0.0120725809859155,0.239324066901408,0.00167152112676057
7.79057344108973,0.254063011554175,-6.02513186947406,-5.81718003048528,1.69780394375129,25.3954790097938,0.00834358098591553,0.219217066901408,-0.0156384788732394
7.94973407653541,0.470788799280845,-5.72087845293728,-5.82807973134306,1.71808616760125,25.7537042317686,0.0229915809859155,0.128136066901408,-0.0533234788732394
7.96878566785591,0.302348121732514,-5.80641227817144,-4.74961101672217,0.937219231353685,25.3193168786227,0.00634658098591553,-0.0208709330985916,0.0145885211267606
7.89650199060342,0.268587406694457,-5.91575135179721,-6.75171736104106,-0.348463955489898,25.6609412006657,0.000886580985915528,-0.0518969330985916,-0.0244274788732394
7.79540328759269,0.316612961012721,-6.02033914358956,-6.75171736104106,-0.348463955489898,25.6609412006657,-0.0110974190140845,-0.0403119330985916,-0.0188344788732394
7.79063260936825,0.374355631952833,-6.00606842162401,-7.36482854250695,1.29912146749223,26.3102644470519,-0.0158914190140845,-0.0204709330985915,-0.00977947887323943
7.81469145732979,0.379181531093542,-5.99178457034827,-5.89295659502144,2.7985438582407,26.1752764703697,-0.00390641901408448,-0.0236669330985916,-0.00192347887323943
7.79541778826289,0.34548346329176,-6.01557473185565,-5.08257476572644,0.934870483707013,24.9724061388571,-0.00976541901408447,-0.0568239330985916,0.0163195211267606
7.76649938534393,0.306961156928047,-6.05837557052095,-5.82807973134306,1.71808616760125,25.7537042317686,-0.0164234190140845,-0.0778629330985915,0.0184505211267606
7.74250533081223,0.379133022445095,-6.04412551778455,-5.82807973134306,1.71808616760125,25.7537042317686,-0.00949941901408448,-0.169077933098592,-0.00951347887323943
7.51615774234104,0.427026628369183,-6.3198412790448,-4.22265522166484,1.36942654502322,24.080242813778,0.0140695809859155,-0.271876933098592,-0.0153724788732394
7.29936225298232,0.349819326727064,-6.61460725406663,-4.15184445655056,2.66831341755165,25.4022746886807,-0.0314704190140845,-0.334195933098592,0.0237765211267606
7.07783476587662,0.330367824920253,-6.8713622743062,-3.2564613308024,1.4373197142395,26.1956569190199,-0.0507794190140845,-0.405702933098592,0.00300352112676057
6.71681807361172,0.464795202909206,-7.20896294839946,-1.37966269167757,1.52551880244625,26.3231200419591,0.00235158098591552,-0.501045933098592,-0.0470644788732394
6.31730733771702,0.440442079380088,-7.54671718842906,-1.37966269167757,1.52551880244625,26.3231200419591,0.00994158098591553,-0.627813933098592,0.0526725211267606
5.70596350264172,0.290831874721171,-8.07480031994232,0.84099980258448,1.59579259946053,26.4392743616538,-0.0124294190140845,-0.666030933098592,0.0544035211267606
5.25373509008348,0.35793049750297 2,-8.32711870973136,3.24423400477745,1.15743523987592,27.0554362566731,-0.0417244190140845,-0.570155933098592,-0.0144404788732394
4.81106952715274,0.429823270740758,-8.61265433531334,3.65297454453319,0.0590373635449687,26.6225533099576,-0.0651604190140845,-0.546053933098592,-0.0563854788732394
4.3493351143965,0.602798140900443,-8.81269571162886,6.1493387094748,1.83777418860113,26.1165253206675,-0.0719514190140845,-0.493322933098592,-0.0328164788732394
3.8730621514564,0.564049136436835,-9.05561807122839,6.1493387094748,1.83777418860113,26.1165253206675,-0.0553064190140845,-0.496251933098592,0.0229775211267606
3.46423651309193,0.559048380260911,-9.2081996889642,9.22230052192819,1.71693988057036,26.7311823667523,-0.0411914190140845,-0.555774933098592,0.0585315211267606
2.85818742381755,0.52508246973462,-9.4465732437474,12.1383045419385,2.37700361141509,25.4340476711379,-0.0351994190140845,-0.547651933098592,0.0454815211267606
2.39185430564277,0.68370148805558,-9.51368513807163,12.718724807375,0.79025645742699,25.8593983140865,0.000620580985915525,-0.491857933098592,-0.0728974788732394
1.91095616284655,0.71237108663669 4,-9.65211566352565,14.7682297455639,2.30764319250819,23.3895461434605,-0.0209514190140845,-0.439791933098592,-0.0954014788732394
1.51188596575467,0.702615749533742,-9.71447255277615,14.7682297455639,2.30764319250819,23.3895461434605,-0.0174894190140845,-0.391188933098592,-0.114843478873239
1.20903991650074,0.798753722836597,-9.73863098312302,14.8478133765521,1.51972905790847,21.9144148596091,0.00568058098591553,-0.360694933098592,-0.0585164788732394
0.728199119047053,0.721605934400641,-9.82489578137421,16.2772457665848,0.387820533994758,21.8058266070884,0.0433655809859155,-0.342851933098592,-0.0495944788732394
0.343530426515421,0.66373913123378,-9.89200479082687,17.3910610560272,2.57139765594721,21.0079343668377,0.0614745809859155,-0.238054933098592,-0.0603804788732394
0.338728241361711,0.586765645780222,-9.8778142070168,17.9360028034348,2.65020896352597,19.7477404067266,0.0324455809859155,-0.0669439330985916,-0.0366784788732394
0.338776346310485,0.567540985486516,-9.84458839568884,17.9360028034348,2.65020896352597,19.7477404067266,0.0142035809859155,-0.0259309330985915,0.00939552112676056
0.223365360849472,0.533819696994983,-9.86852926643373,17.3550761460787,3.27818394682525,21.0501658693787,0.0345765809859155,-0.0107499330985915,-0.0250934788732394
0.343548912508677,0.49055568900217,-9.85412198330289,17.5703536745154,2.70870588359007,20.4754996503019,0.00687958098591553,0.0498370669014084,0.00753052112676056
0.396424285954847,0.490568460345164,-9.85403909345831,18.0280980381221,2.94977456689473,22.0811001349437,0.0340435809859155,0.0475740669014085,0.00912852112676056
0.458950039718358,0.51465092211512,-9.83493520935935,17.6314237430292,1.31541651806586,20.749261926491,0.0389705809859155,0.0237380669014084,0.00540052112676057
0.49255925746516,0.418429149093318,-9.84443488991827,17.6314237430292,1.31541651806586,20.749261926491,0.0934335809859155,-0.0160769330985915,0.00686552112676056
0.391449264661689,0.134502874695017,-9.90648570214753,16.3272913582426,3.64480855243898,20.3887629296605,0.0992925809859155,-0.0336539330985916,-0.00352147887323943
0.372361750213593,0.341416492251304,-9.84941694667688,17.3261843198124,3.65185510940165,21.4295024162704,-0.0186874190140845,0.000435066901408453,0.00180552112676056
0.386803252112443,0.30294138271734,-9.83042417334497,17.1365273610642,4.49359993841955,20.5923884350795,0.0244565809859155,-0.0186069330985916,-0.00804847887323944
0.357877291624436,0.201874937114486,-9.8685133165141,18.0357121764796,3.57542431979907,20.6904355116764,0.0403025809859155,-0.00435893309859155,0.00446852112676056
0.386738301609666,0.221133008790411,-9.8589609021043,18.0357121764796,3.57542431979907,20.6904355116764,0.00634658098591553,0.0309290669014084,0.00686552112676056
0.463680217676559,0.230785116044668,-9.83984190172641,18.6945474826769,3.91323613767161,21.0471560448631,0.000487580985915531,0.0188110669014084,0.00433552112676057
0.372324028853642,0.216320738352168,-9.85423789250053,17.957280895548,1.6508801089901,20.7590680087323,0.00781158098591553,-0.0219359330985915,-0.00458647887323944
0.37707790439445,0.182626653125403,-9.88273980943842,18.8842044414252,3.07149130865372,21.884270026054,0.00181958098591553,-0.00049693309859155,-0.00511947887323944
```

```
0.41076714442685,0.168217903130588,-9.85420587586997,18.7632314128073,3.14560422692185,19.9302499829864,-0.00390641901408448,0.00469606690140845,-0.00125747887323943
0.415570308680888,0.182650075317966,-9.85893830269353,18.1463053638903,4.48036526660067,21.2749073668717,8.85809859155207e-05,-0.00369293309859155,0.000473521126760565
0.410749181955021,0.18745387047836,-9.86843890681249,18.1463053638903,4.48036526660067,21.2749073668717,0.00155258098591553,-0.00129593309859155,-5.94788732394408e-05
0.425175042047127,0.177837956169368,-9.86367450213211,17.2978131945425,4.98429809985726,20.0810697106177,0.000886580985915528,0.00256606690140845,-0.000591478873239432
0.420357811145379,0.163400130992559,-9.86843796816207,18.2291748022537,3.04651151267278,20.8322202464035,0.000753580985915533,-0.00116293309859155,-0.000991478873239443
0.405955717063316,0.177836139087981,-9.85895620005628,18.1964758590099,3.10735788774139,21.9068906758671,0.000354580985915523,-9.79330985915461e-05,-0.000192478873239435
0.41558189601697,0.168221890570732,-9.84945088840938,18.90271784457,3.67686683605947,20.1353817024741,-0.000444419014084471,0.00123406690140845,0.000207521126760563
0.41076193041045,0.177837299964872,-9.85894866561725,18.90271784457,3.67686683605947,20.1353817024741,8.85809859155207e-05,-0.00049693309859155,0.0010065211267606
0.39637663768087,0.168225726478776,-9.83523669348005,18.1043957210652,2.80779941501879,19.5735309121655,-0.000577419014084479,-0.000230933098591547,-0.000192478873239435
0.405968465518746,0.168219568574494,-9.84946595886105,18.1147741441865,1.82875272503662,20.9430826888538,-0.000311419014084477,0.000701066901408448,-0.000192478873239435
0.410783375755409,0.173034165874101,-9.84470715512206,17.4656849619,4.18312384803287,21.6346330255511,-0.000710419014084473,-0.00102993309859155,-0.00152347887323943
0.401191390844599,0.168229714509212,-9.83048071448824,17.6776623956986,4.2595785194354,20.0275278831703,-0.000844419014084469,0.00016906690140845,0.000473521126760565
0.396347657654426,0.17302502952663,-9.85422565601348,17.6776623956986,4.2595785194354,20.0275278831703,-0.000577419014084479,0.00016906690140845,-5.94788732394408e-05
0.420376364712632,0.177839622203566,-9.85893359359198,16.9354364371332,4.39685587076603,21.8412693745304,-0.000178419014084469,-9.79330985915461e-05,-0.00139047887323944
0.405959769960369,0.16340512801995,-9.85421623722009,17.8673188227788,3.41784186665763,20.8646465278996,-0.000311419014084477,0.000568066901408454,-0.000858478873239435
0.410729465937267,0.168203769165433,-9.87794709923459,17.702226403991,2.61431074147198,22.0712944713948,-0.00137641901408447,-9.79330985915461e-05,7.45211267605683e-05
0.405937163496063,0.163396647876974,-9.86846057462638,17.702226403991,2.61431074147198,22.0712944713948,-0.000577419014084479,-0.00049693309859155,-0.00112447887323944
0.401163570363893,0.17784063120052,-9.8494678416418,18.2002824761853,3.42019079219306,21.2115604095416,8.85809859155207e-05,3.60669014084491e-05,-0.00072447887323944
0.425172720050889,0.168215732423995,-9.863680155364,17.0041202716659,3.62922413790502,20.72436645453,-0.000977419014084477,3.60669014084491e-05,-5.94788732394408e-05
0.410783375755409,0.173034165874101,-9.84470715512206,18.916903384669,3.01064493358511,20.8095995965904,0.000886580985915528,-0.00102993309859155,-0.00072447887323944
0.410769466423088,0.177840126875962,-9.85420022263809,18.916903384669,3.01064493358511,20.8095995965904,-0.000444419014084471,-0.00102993309859155,0.000207521126760563
0.405949911951493,0.153780077363487,-9.85897033343115,18.916903384669,3.01064493358511,20.8095995965904,-0.000444419014084471,-9.79330985915461e-05,0.000739521126760567
0.49247334093733,0.0623958185711678,-9.84464406599129,18.222081846035,3.3796264037894,20.4951110128843,-0.0288074190140845,-0.0211369330985915,0.0390895211267606
0.199307447786738,0.341371868785939,-9.8544356741229,16.728939525044,2.87953252266857,19.6187721799257,-0.101113419014084,-0.0917119330985915,0.099015211267606
0.295357205640162,0.259578234815492,-9.89706702258222,17.4656849619,4.18312384803287,21.6346330255511,-0.111766419014084,0.00509606690140845,0.0571995211267606
0.362717630344521,0.432808020444375,-9.88261542774163,18.9566950073906,2.61669136926998,20.0720346858477,-0.245991419014084,0.0286650669014085,0.0209805211267606
0.401381733023536,0.769654246240245,-9.80163870176236,18.9566950073906,2.61669136926998,20.0720346858477,-0.291798419014084,0.0112210669014084,-0.0815534788732394
0.339059434769275,1.17862166094453,-9.75876437163716,17.8248873694784,0.786503601117738,20.8910455820601,-0.313237419014084,-0.00382593309859155,-0.0639754788732394
0.315124725571832,1.43843806786717,-9.73490917631007,18.1398594302154,1.14224941320543,21.2590722784153,-0.311639419014084,0.00309806690140845,-0.0332154788732394
0.324837803300861,1.72231583778647,-9.7157345485412,17.6963006067076,0.234958827426408,20.3276896878899,-0.324289419014084,0.000568066901408454,0.013789521126760
0.315333008666186,1.95808531992238,-9.68237399416634,17.8222475388998,-2.23878822873727,20.1798797254778,-0.353585419014084,0.0113540669014085,0.00593352112676056
0.373253384332063,2.31899972591795,-9.58711063858171,17.8222475388998,-2.23878822873727,20.1798797254778,-0.369830419014084,0.00469606690140845,0.019382521126760
0.2823692056804,2.8675632618256,-9.41599951867868,18.9791403672431,-1.09510359452746,19.6768615607033,-0.352120419014084,-0.0106169330985915,0.00819652112676056
0.373778225258243,3.02638762869531,-9.36353316629419,17.378674792931,-3.14606935361021,19.248494972865,-0.286605419014084,-0.00875293309859155,0.0603955211267606
0.3691478958125,3.29104856083635,-9.29216453791227,18.284951242077,-4.39735286614851,19.6836517299207,-0.287670419014085,0.00869106690140845,0.062526521126760
0.398197951951781,3.53167468784645,-9.19701607118963,18.3955599117752,-3.49241179467023,20.2681220872658,-0.300720419014085,0.0213410669014085,0.0456145211267606
0.45620584038322,3.84932533210977,-9.04005236283933,18.3955599117752,-3.49241179467023,20.2681220872658,-0.322425419014085,0.0407830669014084,0.0204475211267606
0.523711785012313,4.09477236217131,-8.94483979429412,19.3814199600435,-5.531609382512,19.2281486077622,-0.321493419014084,0.0149490669014085,0.0205805211267606
0.55277110491783,4.3113480800985,-8.84021055805044,17.5155214259809,-5.64009863362383,18.7424597884783,-0.300986419014085,0.0104220669014085,0.0127245211267606
0.49056238745261,4.62895273886459,-8.71192291913497,17.3215365217952,-6.06995018280793,20.3284531315721,-0.276085419014084,0.0118870669014084,-0.0111114788732394
0.476391069580081,4.85034092932314,-8.58836490345646,18.153724598202,-7.97418847731296,18.4091300384645,-0.263968419014085,0.00802506690140845,-0.0420044788732394
0.46225105348778,5.07655103575708,-8.44581227260292,18.153724598202,-7.97418847731296,18.4091300384645,-0.244127419014084,-0.00608993309859155,-0.0380094788732394
0.447997392664568,5.20650297531144,-8.36029253334275,17.8894438610445,-8.74417007756572,16.9453132093918,-0.280213419014084,0.0142840669014084,-0.0232284788732394
0.457887401822753,5.44714989792524,-8.22244218840942,19.3084842964931,-8.89703126343568,15.4671725790134,-0.285540419014084,0.00709306690140845,-0.0286884788732394
0.487146721495067,5.71190941266873,-7.99908055106509,16.9189216524061,-10.0836517452251,17.2530058555125,-0.254114419014084,-0.00875293309859155,-0.00924747887323944
0.415190776701951,5.88513662192192,-7.93261857820895,17.2338941173475,-9.72791387219256,17.6210320774977,-0.260639419014084,-0.0132799330985916,-0.0365444788732394
0.377058412517592,6.09692505027299,-7.7616234290115,17.2338941173475,-9.72791387219256,17.6210320774977,-0.301386419014084,-0.0143459330985915,-0.0204324788732394
0.343911840735207,6.42423986881217,-7.49558999344216,16.5537802565541,-9.06639058475662,16.2529895345433,-0.295394419014084,0.00722606690140845,0.0161865211267606
0.435617618739837,6.60721458252065,-7.28642377868584,16.8083130145738,-10.9885928759997,16.6685344508753,-0.234140419014084,0.00949006690140845,0.0100615211267606
0.416538185624672,6.72272586915436,-7.21041694153961,17.6933264364834,-11.2405403440597,16.0923646177331,-0.200051419014084,0.0270670669014084,-0.0357464788732394
0.431313068021786,6.91048413636697,-7.01561181309811,19.0867597886341,-11.6656697582671,16.0260078713719,-0.173419419014084,0.0262680669014085,-0.0340144788732394
0.42673933865498,7.03084061012145,-6.88734964341599,19.0867597886341,-11.6656697582671,16.0260078713719,-0.112698419014084,0.00496206690140845,0.00633252112676057
0.450691095165793,7.00195047193501,-6.93481038198398,17.220860850367,-11.7741510703238,15.540319526458,-0.0710194190140845,0.0158820669014085,0.0119255211267606
0.465249725153594,7.07416837198188,-6.85877623632543,17.6142641150976,-9.49386180262398,15.8397178556295,-0.0792754190140845,0.00576106690140845,0.0141885211267606
0.475095048536723,7.16085287005071,-6.7257628053802,17.9194891336401,-11.8302998732614,15.1594736546702,-0.0359984190140845,0.00216606690140845,-0.00139047887323944
0.494245235692879,7.15120601847813,-6.77321977192344,19.0981807672283,-10.7271875159848,13.9400056507342,0.00701258098591553,0.00376406690140845,0.00420152112676056
0.475007512883596,7.14157632358674,-6.77800401061144,19.0981807672283,-10.7271875159848,13.9400056507342,0.0165995809859155,0.00123406690140845,7.45211267605683e-05
0.470197973736267,7.13195293872202,-6.77801719985597,17.6540560914982,-9.88782231361233,15.1021525298129,-0.00577141901408447,0.000435066901408453,0.00739752112676056
0.494263198164708,7.13197005113036,-6.75898674098093,18.7467032108747,-11.3349038519821,15.3419837089186,-0.00457241901408448,0.00229906690140845,0.00113952112676056
0.460554402334707,7.13193931026201,-6.79702405916187,16.8083130145738,-10.9885928759997,16.6685344508753,-0.0194864190140845,0.00762606690140845,-0.0105784788732394
0.460621651946157,7.16082959973809,-6.75902252963623,17.4960415969891,-11.0244594550873,16.6459138010623,-0.0112304190140845,-0.000896933098591547,-0.00591847887323944
0.465486262030975,7.18009499554522,-6.72576656936809,17.4960415969891,-11.0244594550873,16.6459138010623,0.0504225809859155,0.00229906690140845,-5.94788732394408e-05
0.43172585524905,7.12231476879523,-6.78757903148554,19.7673954148306,-11.3684214462381,15.6662779880396,0.171331580985916,-0.0131469330985916,0.00326952112676056
0.45059078974328,6.9297501883786,-6.98708168934483,17.2992921312987,-9.84960685951479,15.4716870294021,0.331390580985916,-0.0170089330985915,0.0105935211267606
0.498297153963967,6.6794638799882,-7.1770771614036,18.9009109926107,-10.5110994370701,14.493555845871,0.470409580985916,-0.0110169330985916,0.0361605211267606
0.502272399968372,6.19807024652351,-7.62842066679913,17.1090998119527,-9.9666328633077,16.3623469679126,0.646846580985916,-0.0252649330985915,0.021512521126760
0.506355028681808,5.69747356072808,-8.00855478532214,17.1090998119527,-9.9666328633077,16.3623469679126,0.683598580985916,-0.00209493309859155,-0.025359478873239
0.49076012916503,4.85513523475331,-8.62157658846309,17.9962313814293,-8.15206115265574,18.2251143110509,0.700243580985916,-0.0429749330985916,-0.0152394788732394
0.355490989259742,4.23906344715054,-8.95451562097202,18.34390238541,-7.85716171563666,17.5184705779425,0.736995580985916,-0.0814579330985916,0.00872952112676056
0.316648291347041,3.72415068054816,-9.12106196730398,18.0876805305161,-4.18126479600444,20.2372009096314,0.753507580985916,-0.0562909330985915,0.0101945211267606
0.248913872764306,3.21883338485322,-9.32088399959319,18.1878966678191,-2.29728514880137,19.4521204819026,0.691588580985916,-0.0506979330985915,0.0109935211267606
0.219639347977241,2.57882435943685,-9.4969846642763,18.1878966678191,-2.29728514880137,19.4521204819026,0.612358580985916,-0.0405779330985916,-0.00924747887323944
0.180808216086666,1.9869431004813,-9.64458345725849,17.8729388624792,-2.65302401690234,19.0840895927604,0.625274580985916,-0.0377819330985915,-0.0189674788732394
0.15642029204967,1.26999117894762,-9.75899701248184,18.3164975585234,-1.74573319393831,20.0154763724543,0.544313580985916,-0.0516309330985915,-5.94788732394408e-05
0.12253107962360,0.865783890447314,-9.84950068013148,19.5082219779496,1.40362354532772,20.8767255238002,0.413416580985915,-0.0292599330985915,-0.00471947887323944
0.122467201352224,0.538623889705584,-9.84019600996021,18.2689664249754,2.65255794835765,20.0946553356607,0.357889580985916,-0.00782093309859155,-0.0125764788732394
0.11274001095789,0.129658845682281,-9.84994823610404,18.6559082100118,1.59473135657803,20.3782221688104,0.419409580985916,0.00988906690140845,-0.0316184788732394
0.098210226091003,-0.322603219575552,-9.8502365527311,18.6559082100118,1.59473135657803,20.3782221688104,0.409821580985915,-0.0124819330985916,-0.0318844788732394
0.0597049386860539,-0.688253928695623,-9.82677143358288,17.5659009530798,6.06710466550658,20.8495557989949,0.445375580985916,-0.0183409330985915,-0.0253594788732394
0.0259675290428435,-1.27521600231506,-9.79393192250273,18.4290995547182,5.85572140187729,20.9898358130682,0.471874580985916,0.00522906690140845,-0.0477304788732394
0.0740521944694769,-1.67930844982734,-9.72287232347879,18.4443275546923,7.1070286095561,18.208502851735,0.446973580985916,0.0100230669014085,-0.0317514788732394
0.122172963469115,-2.05452091043793,-9.63280397629798,18.6366389266827,9.29057386012792,19.7567821522233,0.436719580985916,-0.00342693309859155,-0.0503934788732394
0.107797962050732,-2.45861714506813,-9.54285055028967,18.6366389266827,9.29057386012792,19.7567821522233,0.366145580985916,-0.0154109330985915,-0.0192344788732394
0.0694294745509651,-2.8194053590966,-9.43391657979266,19.6971227533603,8.86310270155682,19.3435115206016,0.358554580985916,-0.00875293309859155,0.0181835211267606
-0.0074343536993770,-3.28605699526224,-9.33460196654618,18.4469820130066,10.132319503639,18.9196650884524,0.358954580985915,-0.0178079330985915,0.0171185211267606
-0.0265736353188918,-3.57467803335664,-9.23509134944837,20.2948883447227,10.5941969382803,19.4264693625255,0.306223580985916,-0.00648893309859155,-0.00778247887323943
0.0841019608370248,-3.68044509714783,-9.14476575365132,19.2600105050702,11.2939366128995,18.4279603311645,0.298632580985915,-0.00888593309859155,0.0157865211267606
-0.0071360949970883,-4.0171900207948,-9.03590053962404,19.2600105050702,11.2939366128995,18.4279603311645,0.259483580985916,-0.0484349330985915,0.0233775211267606
-0.0839256171857033,-4.17592145460941,-8.93640501764457,21.645780481926,12.1677326009759,17.337461565837,0.201426580985916,-0.0395129330985915,0.00127252112676056
-0.0645927358740251,-4.32982270225274,-8.84625185492693,20.2267256736691,12.3205942482479,18.8155974377434,0.215274580985916,-0.00289393309859155,-0.0309524788732394
-0.0212074017193555,-4.53663157989258,-8.73709932758537,20.1694625946693,14.026708638465,17.846505468151,0.242972580985916,-0.00142893309859155,0.00034052112676055
-0.0595370056511273,-4.76751443464211,-8.623340594003,20.1154854823742,15.0868831128726,17.9098524254811,0.231253580985916,-0.0143459330985915,-0.0124434788732394
-0.0641785104445882,-4.95507533716033,-8.49051188317954,20.1154854823742,15.0868831128726,17.9098524254811,0.226992580985916,-0.0199379330985915,0.0247085211267606
-0.155377114260392,-5.19077874644369,-8.3720914143146,19.3318701261792,14.5103592478716,16.2944425899222,0.243770580985916,0.00296506690140845,7.45211267605683e-05
-0.14079369047513,-5.48417399994947,-8.22505041092953,18.6119647701278,15.5658358624377,15.6639607685422,0.233517580985916,0.0236050669014084,-0.0354794788732394
-0.063694189158669,-5.69575991956395,-8.07311495032228,19.288792682876,15.5502515577255,15.9995653725698,0.172929580985916,0.00522906690140845,0.0189825211267606
-0.0972802764348272,-5.75347513183877,-8.02572115459502,19.8588185749675,14.9425590266905,15.0553683056347,0.167070580985916,-0.0425759330985916,0.0741115211267606
-0.217374473372307,-5.89779913223701,-7.95477265613016,19.8588185749675,14.9425590266905,15.0553683056347,0.182783580985916,-0.0554919330985916,0.0518735211267606
-0.30373394245774,-6.0324626923582,-7.83153684786288,20.4821912496278,16.9459214917515,13.7265422635415,0.144167580985916,-0.0160769330985915,0.00566652112676057
-0.20265422861036,-6.12379258776737,-7.73172294105223,19.41461483907,17.7064996616804,13.8027042345661,0.0940995809859155,0.0161480669014085,-0.011244478873239
-0.1689796250071,-6.10934214170447,-7.71741638303051,20.0201044084457,15.4332571881282,14.5440495811729,0.110078580985916,0.0134850669014085,0.0148545211267606
-0.226684844956272,-6.17190486250601,-7.7222919987596,20.1274272575624,16.9841370051453,14.0960778104227,0.115937580985916,0.0136180669014084,0.0550695211267606
-0.221651040971848,-6.32576537070891,-7.55619230304359,20.1274272575624,16.9841370051453,14.0960778104227,0.147496580985916,0.0152160669014085,0.0235105211267606
-0.144616991034482,-6.40267288589238,-7.46590249590184,19.2277226143622,16.9435409232497,15.7258030963807,0.164274580985916,0.00909006690140845,0.00233752112676057
-0.22618128878857,-6.61431225822312,-7.33795692878325,21.2914130421245,17.7947067482385,13.9301688791674,0.159347580985916,0.00722606690140845,0.0337635211267606
-0.235704316559591,-6.6768219725658,-7.27153550266566,20.9726339918735,17.126135911458,14.2574718762156,0.111409580985916,-0.0167429330985915,0.00113952112676056
-0.225964558390504,-6.77779897761154,-7.17661787375972,21.5464822251488,16.8312750613919,12.6179367020557,0.105550580985916,-0.0451059330985915,-0.0394744788732394
-0.269184201743956,-6.82109115369862,-7.14347401578983,21.5464822251488,16.8312750613919,12.6179367020557,0.0991595809859155,-0.0732029330985915,-0.0748954788732394
-0.336333042735108,-6.96056633316114,-7.02970777889834,19.7660919912192,18.3142239367328,12.4007266507518,0.0866425809859155,-0.0554919330985916,-0.0549214788732394
-0.259405194889535,-6.95091987948661,-7.02008566290517,20.4609131575146,17.9452503462874,12.7152146615358,0.0850445809859155,-0.0285939330985915,-0.0566524788732394
-0.211234455377226,-6.99416637776333,-6.94881406188044,20.5071516508545,20.8894126441382,11.9934858546757,0.0702635809859155,-0.0222019330985916,-0.0195004788732394
-0.249684558495561,-7.03747152610592,-6.93940289973132,19.8516013425527,19.9056609609818,12.6692042802774,0.0428325809859155,-0.00582293309859155,-0.0159054788732394
-0.230367271641888,-7.03743296154154,-6.88239540920285,19.8516013425527,19.9056609609818,12.6692042802774,0.0512215809859155,0.00988906690140845,0.00220452112676056
-0.182250599505566,-7.09032247524033,-6.84436657821843,20.6401915206057,18.0825516299435,12.182784137787,0.0348425809859155,0.00403006690140845,0.011525521126760
-0.19188720624956,-7.0614684283324,-6.86335746876959,20.7584140387142,19.6131492824069,11.3765881923542,-0.0230814190140845,-0.00688893309859155,0.00513452112676056
-0.254385711129901,-7.066297963058,-6.86820670052971,22.1660325267594,18.5218059047802,11.9844498144794,-0.0190874190140845,-0.00662193309859155,0.00207152112676057
-0.259213368248385,-7.06149598925475,-6.88245475393272,19.2853820676367,20.8261863964684,12.918071080887,-0.00816741901408448,0.0176130669014085,0.00420152112676056
-0.23993899521465,-7.11439246942717,-6.84447113745051,19.2853820676367,20.8261863964684,12.918071080887,-0.0204184190140845,0.00602806690140845,-0.00685047887323943
-0.24962718844939,-7.08074759961435,-6.8966953279414,20.6281391929505,20.8152999010723,13.9475044317588,-0.0264104190140845,-0.0276619330985916,0.00473452112676057
-0.2880703037801,-7.03264375967065,-6.89672734398166,20.8543008894321,20.2255404816923,13.0146145478539,-0.0318704190140845,-0.0158109330985916,0.0133905211267606
-0.244862270892802,-7.03746471175469,-6.92989946929156,20.2385430451976,18.8478354208803,12.9527722200155,-0.0889964190140845,0.0106880669014085,-0.00285547887323943
-0.206532711647462,-6.93166448225155,-7.02473891745447,19.2140582564021,18.5685274697451,13.3238113454777,-0.176215419014084,-0.0115489330985915,-0.0173694788732394
-0.249991100525406,-6.77780195581228,-7.17190710769653,19.2140582564021,18.5685274697451,13.3238113454777,-0.280347419014084,-0.0134139330985915,0.0213795211267606
-0.33684877915038,-6.53740721656799,-7.41880259890792,19.89467875399,18.8657687104241,12.964082544922,-0.368232419014084,0.00642706690140845,0.0325655211267606
-0.274727504582948,-6.19115061847875,-7.70338687079137,20.1819256981194,16.882726004488,12.3049537951334,-0.459047419014084,0.0361220669014084,0.0412205211267606
-0.265436254774095,-5.90261952978331,-7.95010239389403,21.3126911342377,16.7953778937026,14.941496481173,-0.460512419014084,0.0331920669014085,0.0330975211267606
-0.19374437330927,-5.35913341751791,-8.29153305045067,20.081174072755,14.0399757616591,14.817812331732,-0.546800419014084,0.0106880669014085,-0.0674384788732394
-0.271017489444491,-4.80123249926264,-8.60470015270747,20.081174072755,14.0399757616591,14.817812331732,-0.594338419014084,-0.0325889330985915,-0.0360124788732394
-0.285748663115554,-4.33951347170577,-8.87034437198613,20.7002192061824,14.771733909463,15.9120931395534,-0.634686419014084,-0.0200719330985916,0.0220455211267606
```

-0.285952508821499,-3.81039946479954,-9.07894906137157,19.7078966775448,13.4728157476472,16.9362362021932,-0.613247419014084,0.0182780669014085,0.0160535211267606
-0.233252023942394,-3.3486122977944,-9.25902275084456,20.4201892682524,11.7916813905961,18.9573821406047,-0.559184419014084,0.0112210669014084,-0.0113774788732394
-0.23347501612708,-2.86761424597038,-9.47240415054918,19.5434507814987,8.99806199489356,18.4641619069976,-0.492204419014084,0.00216606690140845,-0.00525247887323944
-0.199869850799617,-2.5164314515561,-9.55286263331424,19.5434507814987,8.99806199489356,18.4641619069976,-0.417102419014085,-0.00222793309859155,0.00606652112676057
-0.228741107894524,-2.01612424628578,-9.64757578819519,19.3826715661553,9.46612929465645,17.2477082999535,-0.411776419014084,-0.00129593309859155,0.0167195211267606
-0.238431534237557,-1.74193835916979,-9.73764420393747,19.5647288736118,7.99873314035769,19.4754895090032,-0.403120419014084,0.0240040669014085,0.0478785211267606
-0.180733122726493,-1.27045814396715,-9.7990015625268,20.3064194715795,6.90268495621625,19.3895219373445,-0.423893419014085,0.0234720669014085,0.0180505211267606
-0.185530324075604,-0.823049084070699,-9.86047101636086,19.3108266046478,6.24970566461589,19.3812213740387,-0.424426419014085,0.0333260669014084,-0.0176364788732394
-0.103723664349509,-0.418885714714364,-9.8648539131538,19.3108266046478,6.24970566461589,19.3812213740387,-0.444666419014084,0.0438450669014084,-0.0290884788732394
-0.0699594595859611,0.0959241296432581,-9.86924616017865,19.6302371127637,3.247046683886,19.375195126492,-0.405118419014085,0.0185450669014085,-0.0101794788732394
-0.0987103743247838,0.40384402395959,-9.85961456778325,18.9773420590497,5.28858526697877,20.7620830286938,-0.310574419014084,0.0160150669014084,-0.00565147887323944
-0.0505592379660125,0.654048964760772,-9.84514788838393,19.7550316948993,3.48575797313101,20.6338839508755,-0.275020419014084,0.0166810669014084,-0.0105784788732394
-0.00715284732472784,0.97162828535499,-9.80216049421698,19.1866793536628,2.33975586969264,18.4438093622243,-0.245059419014084,0.00336506690140845,-0.0176364788732394
-0.0743282111980919,1.22182049957093,-9.76413443253481,19.1866793536628,2.33975586969264,18.4438093622243,-0.203247419014084,-0.00489093309859155,0.0105935211267606
-0.0741913442038231,1.41430391866091,-9.70704401532049,18.9599808784977,1.97075163134932,21.1044767546466,-0.125082419014084,-0.0191399330985915,0.0285705211267606
-0.0838894183350127,1.37578342962103,-9.754563154488,20.9234552645546,0.937937631246212,20.0939198232858,-0.0566384190140845,-0.0186069330985916,0.0220455211267606
-0.136827239842311,1.37093743500612,-9.79263344146668,18.3769207000911,0.532208007160568,19.9679613617042,0.00102058098591552,-0.0160769330985915,0.0615935211267606
-0.165663457125975,1.4190397655553,-9.79739882905112,18.8461004974919,1.71175741966719,19.487563277592,0.0538845809859155,0.0265340669014084,0.111795521126761
0.0218016133849066,1.34692150915174,-9.78765145287652,18.8461004974919,1.71175741966719,19.487563277592,0.0490905809859155,0.0676810669014085,0.114858521126761
0.0890923762624709,1.23147654464245,-9.77336945971486,19.5987053284552,0.595426253792397,19.0433700059699,0.0445635809859155,0.0310620669014085,0.0998105211267606
0.137128637865835,1.19298977501951,-9.78756105680703,19.5987053284552,0.595426253792397,19.0433700059699,0.0203285809859155,0.0415810669014084,0.0165855211267606
0.170853119946557,1.28924294118716,-9.75421465598431,17.5491858262625,-0.921959664109155,21.5132237018765,0.0187305809859155,0.0100230669014085,0.0139225211267606
0.10339300404513,1.14002964347899,-9.83512536150229,19.1463660490002,1.77495290693068,20.9091517460001,0.0877075809859155,-0.0160769330985915,0.0381575211267606
0.13716572344393,1.15932744511347,-9.7590921691915,19.1463660490002,1.77495290693068,20.9091517460001,0.0750575809859155,-0.0123479330985916,-0.011114788732394
0.137005090439617,0.962023932237062,-9.83042875919199,19.7054927213764,1.1875354158874,20.3231752967975,0.0826475809859155,0.0245370669014085,0.0236435211267606
0.151432090094658,0.894678319833767,-9.81620138912519,19.0935416404406,0.12266208925636,19.5659995014609,0.0353755809859155,0.0498370669014084,0.0364265211267606
0.185096264091471,0.928367558974949,-9.81138140212924,18.2613527226883,2.02690019710194,21.4853184372659,0.0143365809859155,0.00696006690140845,0.0358945211267606
0.194695193919495,0.899499378692149,-9.81613074341147,18.9142631498858,-0.0146389572147835,20.0984342143782,0.0789185809859155,0.00482906690140845,-0.0310854788732394
0.156205067427775,0.784017564442806,-9.82100731032443,20.3283431176251,2.23213352463797,20.7221273325252,0.132981580985916,0.00895706690140845,-0.00498647887323944
0.184973263559155,0.605996558949129,-9.84005847428656,20.3283431176251,2.23213352463797,20.7221273325252,0.180919580985916,0.0185450669014085,0.00699852112676057
0.252219360159555,0.399128472188392,-9.84007452630013,19.5480136006713,1.0096699810126,20.1391606130574,0.225261580985916,0.0236050669014084,0.00619952112676056
0.271348738606048,0.120068278011884,-9.85920111481958,18.0575115463293,3.53485993139541,19.973986115019,0.240175580985916,-0.000629933098591551,0.00313652112676056
0.194334268692199,-0.159016958800889,-9.8832268533918,19.4651445665123,2.44351579588536,20.5818472591556,0.200360580985916,-0.00249393309859155,0.00380252112676056
0.208666153811108,-0.370723564513229,-9.90706885399444,19.6084377110481,3.28761113158591,20.0916455704414,0.0750575809859155,0.0290640669014085,-0.000325478873239443
0.304891027172306,-0.288883489753574,-9.86413707859132,19.6084377110481,3.28761113158591,20.0916455704414,-0.0648944190140845,0.0330590669014085,-0.00165747887323944
0.324177513599783,-0.0771800631544891,-9.85923411790166,19.0814893127854,2.85541036038512,21.3307197954327,-0.106173419014084,0.000435066901408453,-0.00218947887323943
0.30021202614354,0.023866952908908,-9.83072376116814,18.7632314128073,3.14560422692185,19.9302499829864,-0.0692884190140845,-0.0357849330985915,-0.000991478873239443
0.218447252692421,0.0430728803505697,-9.864077674679,19.3038442008961,1.95281839233104,21.093166461606,-0.0214834190140845,-0.0415099330985915,-0.000325478873239443
0.155935842283721,0.0430493068683612,-9.87841997238154,19.0253789880466,1.84905846613833,18.9551285646747,0.0104745809859155,-0.00608993309859155,0.000473521126760565
0.228091238133668,0.00941004636242996,-9.84034116648616,19.0253789880466,1.84905846613833,18.9551285646747,0.0227255809859155,0.0254690669014084,-0.000591478873239432
0.261705691210825,-0.0338908158877257,-9.85455819636343,19.7022069326608,1.83347410212993,19.2907321214101,0.0215265809859155,0.0157480669014084,-0.00072447887323944
0.266499566994999,-0.0579495481260828,-9.85931323729512,19.6695078619534,1.89432071438355,20.3654067400422,0.00661258098591553,-0.00355993309859155,0.000473521126760565
0.232813376055695,-0.0579718094417551,-9.88310720960098,19.1463660490002,1.77495290693068,20.9091517460001,-0.00989841901408448,-0.00808693309859155,0.000606521126760559
0.232871362143366,-0.00503010616065488,-9.85458644900609,20.013372153161,1.87638742483971,20.3540964151356,-0.0105644190140845,-0.00049693309859155,-0.000192478873239435
0.242460867742547,-0.0146594925324308,-9.86882136820928,20.013372153161,1.87638742483971,20.3540964151356,0.00381658098591553,0.00016906690140845,-0.000192478873239435
0.242492739546697,-0.038701420882063,-9.84509526975074,19.604630998275,2.974778405023,20.7869789786433,0.00701258098591553,0.00150006690140845,-0.00072447887323944
0.252083563580616,-0.0387075790288005,-9.85932453670535,18.7627102306686,2.18683958290088,21.658023839773,-0.000844419014084469,0.00229906690140845,0.000340521126760557
0.256894263847292,-0.0242725799303328,-9.85930852054974,19.1991754058893,3.42723740845198,22.2523009434436,-0.00457241901408448,-0.00182893309859155,-0.000591478873239432
0.232882949479448,-0.0194582909078892,-9.84509903472195,18.0177195699286,3.92882044238376,20.7115514408356,-0.00150941901408447,-0.00289393309859155,-0.000192478873239435
0.232881788360101,-0.0242699051416362,-9.84510186163304,18.0177195699286,3.92882044238376,20.7115514408356,0.00275158098591553,0.00256606690140845,-0.000325478873239443
0.247289687553987,-0.014649815125635,-9.85456949636397,17.5381758815445,3.7283169619759,19.822397270271,8.85809859155207e-05,0.00083506690140845,0.000207521126760563
0.237682630617901,-0.0194599566996317,-9.84983994168848,18.3300365253551,1.25926852353719,20.3684165645577,-0.00270841901408447,-0.00236093309859155,-0.000458478873239437
0.237657701914901,-0.0290916653101015,-9.86408993291694,18.4046603037643,2.87099495280784,20.9951194124395,-0.00137641901408447,3.60669014084491e-05,-0.000325478873239443
0.237647275698165,-0.0098518663291202,-9.87357452028999,18.3757684774981,3.24466611538425,21.3744559593312,0.00181958098591553,0.00176706690140845,-0.000192478873239435
0.237676257055763,-0.0146521735088019,-9.85458456681563,18.3757684774981,3.24466611538425,21.3744559593312,0.00315058098591553,-0.00102993309859155,-0.000192478873239435
0.232849323886919,-0.0339034359410752,-9.86410029525037,17.8384269009149,3.79151320712279,21.2439862166677,-0.000577419014084479,-0.000230933098591547,-0.00072447887323944
0.228041381212579,-0.00985136141426911,-9.8688411477625,18.2149892621548,3.71273341514714,20.1580023522871,-0.00457241901408448,-0.00169593309859155,0.000340521126760557
0.237672773940177,-0.0290860114879226,-9.85459304695861,19.0455048070413,3.56218871220803,21.3729508236036,-0.00230841901408448,0.00150006690140845,-0.000858478873239435
0.242467241304685,-0.0194672757232606,-9.86407674308212,19.776831064749,3.44519358472735,19.9174345542182,0.00261858098591553,0.00296506690140845,0.000207521126760563
0.252123562492828,-0.0290712219085646,-9.83557865164007,19.776831064749,3.44519358472735,19.9174345542182,0.00261858098591553,0.00083506690140845,0.000340521126760557
0.232856859899557,-0.0339006090299857,-9.85935185227121,18.1185812611641,2.14157751254442,20.2477488062819,-0.00164341901408448,3.60669014084491e-05,-0.00112447887323944
0.252093421589492,-0.0290825283723373,-9.8545704404943,16.7708646688161,4.55209756584149,21.3201481247775,-0.00310741901408447,-0.000762933098591552,-0.000591478873239432
0.242483474206853,-0.014651012389455,-9.85457703080299,18.5801316773415,2.69547121177118,21.1580185820941,0.000487580985915531,0.000302066901408451,0.000207521126760563
0.242458545746309,-0.0242817162778045,-9.86882702144116,19.0106721702113,1.55651590248049,20.0086876692213,0.00275158098591553,0.00123406690140845,0.000207521126760563
0.228058182565061,-0.0338989429957877,-9.85461094373107,19.0106721702113,1.55651590248049,20.0086876692213,-0.00164341901408448,0.00016906690140845,-0.00139047887323944
0.232865556789086,-0.0290871726072695,-9.85460058297125,17.8962111807131,3.04416252784109,20.4853053174694,-0.00177641901408447,-0.000896933098591547,0.000739521126760567
0.237661185030486,-0.0146578273309808,-9.86408145277396,18.6836479092828,3.93351812433656,21.4053770776694,-4.54190140844746e-05,0.000568066901408454,-0.00072447887323944
0.237658863034248,-0.0242800510763545,-9.86408710600585,19.0814893127854,2.85541036038512,21.3307197954327,0.000620580985915525,0.00083506690140845,-0.000325478873239443
0.232856859899557,-0.0339006090299857,-9.85935185227121,19.4438664425332,3.44284470971748,19.5705207044421,8.85809859155207e-05,-0.000230933098591547,0.000739521126760567
0.237623508114512,-0.014671960705843,-9.88782168460737,19.4438664425332,3.44284470971748,19.5705207044421,-0.00164341901408448,-0.000896933098591547,-5.94788732394408e-05
0.237662915931301,-0.0387110621443858,-9.85934714316966,18.2722522960825,2.00661821043699,21.1270974044597,-4.54190140844746e-05,0.00110106690140845,-5.94788732394408e-05
0.237679147502315,-0.0338937946787524,-9.84984842183145,18.2689664249754,2.65255794835765,20.0946553356607,0.000620580985915525,-0.000230933098591547,-0.00205647887323944
0.21844374826288,-0.0339012652344816,-9.85462601575635,19.1539802829554,2.40060248194626,19.5184839808563,-4.54190140844746e-05,-0.00049693309859155,7.45211267605683e-05
0.237672773940177,-0.0290860114879226,-9.85459304695861,18.8738255690272,4.05054512319787,20.5147218063159,-0.00124341901408448,0.00123406690140845,0.000473521126760565
0.232848162767572,-0.0387150501748221,-9.86410312216146,18.8738255690272,4.05054512319787,20.5147218063159,-0.000311419014084477,3.60669014084491e-05,-0.000458478873239437
0.237653650591457,-0.0146606536517777,-9.86882890422192,18.5659461372426,3.36169311424556,20.4838006879777,-0.000178419014084469,-0.000230933098591547,7.45211267605683e-05
0.24247245507863,-0.029087677296651,-9.85933395392514,19.4471519094358,2.79691291085194,20.6029632476111,-0.000844419014084469,0.000435066901408453,7.45211267605683e-05
0.237635095450595,-0.0291001454530773,-9.87833427032322,17.7387320600079,2.86629709296629,20.3012906337294,-0.000311419014084477,-9.79330985915461e-05,-0.000591478873239432
0.23764379258258,-0.0242857043082409,-9.87358300043297,17.7387320600079,2.86629709296629,20.3012906337294,0.000753580985915533,3.60669014084491e-05,-0.000458478873239437
0.237681469498554,-0.0242715709333787,-9.84984276859957,18.7120189070891,2.60107537106595,22.7538139724905,0.000221580985915529,0.000568066901408454,-0.000458478873239437
0.242474777317324,-0.0194644488121712,-9.85932830010296,18.2722522960825,2.00661821043699,21.1270974044597,-0.000444419014084471,-0.000363933098591548,-0.000458478873239437
0.237650167475871,-0.0290944916308984,-9.86883738436489,18.7556176786543,2.51994653496237,21.3209141318838,-0.000311419014084477,-0.000230933098591547,0.000473521126760565
0.228039059216341,-0.0194735851596428,-9.86884680099438,18.4662517005401,2.43646906103394,19.5411046306694,-0.000444419014084471,0.00016906690140845,-0.000192478873239435
0.256899477863692,-0.0338919767646171,-9.85456573080246,18.4662517005401,2.43646906103394,19.5411046306694,-0.000710419014084473,-9.79330985915461e-05,-0.000192478873239435
0.237673935059524,-0.0242743972541756,-9.85459022004752,18.0542257576137,4.18079861763794,18.9415429396317,-4.54190140844746e-05,0.000302066901408451,-0.000192478873239435
0.237687844391845,-0.0290803582560362,-9.84509715253149,18.8891783449995,0.671857170758171,19.7824400205744,-0.000444419014084471,-0.000363933098591548,-0.000192478873239435
0.247289687553987,-0.014649815125635,-9.85456949636397,18.0749826170626,2.22270611146299,21.6806444577202,-4.54190140844746e-05,-0.000230933098591547,0.000473521126760565
0.228061665680647,-0.019465105016667,-9.8546024635881,19.6264303681246,2.93421401661933,20.070529581986,-0.000178419014084469,0.000435066901408453,-0.000458478873239437
0.237666399046886,-0.0242772241652651,-9.85933866302669,19.6264303681246,2.93421401661933,20.070529581986,-0.00137641901408447,0.00016906690140845,-0.00072447887323944
0.247250848187513,-0.019474594989345,-9.87831354604938,19.787209583468,2.46614671685643,21.2869831890301,-0.000444419014084471,3.60669014084491e-05,-0.000858478873239435
0.223234732269347,-0.0387173721710605,-9.86411819261313,18.7703239648216,2.81249727486034,20.2673596988757,-0.000178419014084469,0.000302066901408451,7.45211267605683e-05
0.242470133082391,-0.0387099010250388,-9.85933960715702,19.1631905464663,4.13402270694812,22.2945323866883,-0.00111041901408447,3.60669014084491e-05,-0.00112447887323944
0.232844111444127,-0.0242840385164984,-9.86884209346644,19.787209583468,2.46614671685643,21.2869831890301,-0.000311419014084477,0.000968066901408451,-0.000325478873239443
0.242486364653406,-0.0338926335594055,-9.84984088581882,19.787209583468,2.46614671685643,21.2869831890301,-0.000577419014084479,0.00016906690140845,7.45211267605683e-05
0.256919192307836,-0.0146418760419833,-9.84505852991157,18.5730390934614,3.02857822312894,20.820909921497,0.000354580985915523,-0.000363933098591548,7.45211267605683e-05
0.247254901084566,-0.0339056060573762,-9.87357358321319,18.4695371674427,1.79053726216839,20.5735471738384,0.000354580985915523,-0.000363933098591548,-0.000192478873239435
0.232849323886919,-0.0339034359410752,-9.86410029525037,18.9245172462856,3.63620338835956,19.4189312585246,-0.000444419014084471,0.00016906690140845,-5.94788732394408e-05
0.237667560166233,-0.0194656099315181,-9.85933581156794,18.3724825613189,3.89060503881178,20.3420169731124,0.000354580985915523,-0.000496933098591548,-0.000858478873239435
0.2280605045613,-0.0242767192500414,-9.85460529049919,18.3724825613189,3.89060503881178,20.3420169731124,-0.000311419014084477,-0.000363933098591548,-0.00072447887323944
0.232856859899557,-0.0339006090299857,-9.85935185227121,18.508161901797,4.10904280114537,21.2424815278797,-0.000977419014084477,0.00016906690140845,-0.000192478873239435
0.237650167475871,-0.0290944916308984,-9.86883738436489,18.5877455070923,3.3211287258419,19.7673512913204,-0.000977419014084477,-9.79330985915461e-05,-0.000991478873239443
0.237665237927539,-0.029088838399012,-9.85934148993777,19.099481969862,2.50201324541854,21.3096038069772,-0.000311419014084477,0.000435066901408453,-0.000192478873239435
0.22804659365537,-0.0194707588388459,-9.86409934954643,19.4819700327876,4.80258554537724,21.9672278679779,-0.000844419014084469,-0.000363933098591548,-0.000858478873239435
0.242443473721033,-0.0242873070999834,-9.8783239933995,19.4819700327876,4.80258554537724,21.9672278679779,-0.000710419014084473,0.000435066901408453,-0.00072447887323944
0.23286787902778,-0.0194639441397756,-9.85459492914907,17.8706047894836,2.7719019508482,21.8970854548222,-0.00137641901408447,0.00016906690140845,-0.000858478873239435
0.237681469498554,-0.0242715709333787,-9.84984276859957,17.8744118108635,3.08472691624476,21.2017547141267,-0.000577419014084479,0.00016906690140845,-0.000858478873239435
0.232881788360101,-0.0242699051416362,-9.84510186163304,18.90271784457,3.67686683605947,20.1353817024741,-0.000444419014084471,-0.000363933098591548,-0.000192478873239435
0.232880627240754,-0.0290815193753831,-9.84510468854413,18.8918181755762,3.69714900061318,20.4936058771567,-0.000311419014084477,-0.000363933098591548,-0.000192478873239435
0.247247365071928,-0.0339084329684657,-9.87832202619235,19.7943021354823,2.13303976479493,21.6240928969193,-0.00150941901408447,0.000435066901408453,-5.94788732394408e-05
0.228088323468397,-0.033887636532015,-9.83561915487684,19.7943021354823,2.13303976479493,21.6240928969193,-0.000444419014084471,-0.000230933098591547,-0.000192478873239435
0.232873091228116,-0.0290843462864726,-9.84985313152329,17.7349253472348,2.55346436640337,20.9966240419312,-0.000311419014084477,-0.000230933098591547,-0.00112447887323944
0.24729257800054,-0.0338914726825141,-9.84983335137979,18.5152549411268,3.77592866522562,21.5795866315267,-0.000577419014084479,-0.000230933098591547,-0.000458478873239437
0.237662915931301,-0.0387110621443858,-9.85934714316966,19.4509590264134,3.10973769835974,19.9076293650393,-4.54190140844746e-05,-0.000896933098591547,-0.000591478873239432
0.232819181409974,-0.0339147429951405,-9.88309307563582,18.2073755598677,3.08707566389143,21.5486654538923,-0.000178419014084469,-9.79330985915461e-05,0.000207521126760563
0.228089484345288,-0.0290770270203882,-9.83561632855604,18.2073755598677,3.08707566389143,21.5486654538923,-0.000444419014084471,0.000568066901408454,0.00127252112676056
0.247312294018293,-0.0146413713695877,-9.84032515895768,18.916903384669,3.01064493358511,20.8095995965904,-0.000311419014084477,0.00136706690140845,0.000739521126760567
0.247287365557749,-0.0242720752579372,-9.85457514959585,18.544146767393,3.40225750264923,21.200250084635,-0.000444419014084471,-0.000496933098591548,7.45211267605683e-05
0.237672773940177,-0.0290860114879226,-9.85459304695861,17.6591489101623,3.65420404370777,21.7764173133386,-0.000311419014084477,-0.000629933098591551,-0.000192478873239435
0.237667560166233,-0.0194656099315181,-9.8593358136156,18.848740732275,4.73704131046708,20.1987286598042,-0.000710419014084473,0.00016906690140845,0.000340521126760557
0.247323881354375,-0.029069556116822,-9.83083774467354,18.848740732275,4.73704131046708,20.1987286598042,0.000354580985915523,0.000302066901408451,-0.00072447887323944
0.252106170044922,-0.0386990988858246,-9.84508019929907,18.4406452137129,2.16420866192981,20.9528879098986,0.000221580985915529,0.000968066901408451,-0.000192478873239435
0.252116028053799,-0.0290740482293615,-9.84032610308802,18.4226529608409,2.51757837841126,20.9740034239841,-0.00177641901408447,3.60669014084491e-05,-0.000325478873239443
0.242478830214377,-0.0338954598802024,-9.85458833726677,18.4913365372924,1.74997287376473,19.8570977711811,-0.000178419014084469,3.60669014084491e-05,-0.00072447887323944
0.237651328595218,-0.0242828773971514,-9.8688345574538,18.594838463311,2.98801383472528,20.1044605248396,0.000487580985915531,3.60669014084491e-05,-0.00112447887323944
0.237685522395607,-0.0387025820014099,-9.84510280576338,18.594838463311,2.98801383472528,20.1044605248396,-0.000844419014084469,0.00016906690140845,-0.000458478873239437
0.237710450856151,-0.0290718781130604,-9.83085281512521,19.8488004804416,2.03162894202641,19.8329720235063,-0.000710419014084473,3.60669014084491e-05,-0.00072447887323944
0.228036736977647,-0.0290968136271368,-9.86885245481656,19.396460681454,3.21114852112826,21.6987502384523,-0.000178419014084469,-0.000363933098591548,-0.000591478873239432
0.237679147502315,-0.0338937946787524,-9.84984842183145,18.8733047405674,3.09177353250367,22.2424952480287,-0.000444419014084471,-9.79330985915461e-05,-0.000325478873239443
0.228017020959894,-0.0483469149400631,-9.87836064723866,18.9278030855267,2.99036270973515,20.4513743746157,-0.000311419014084477,0.00136706690140845,7.45211267605683e-05

```
0.23769132750743,-0.0146465202769155,-9.84508867238852,18.9278030855267,2.99036270973515,20.4513743746157,-0.000977419014084477,0.00083506690140845,-0.00072447887323944
0.261720761662493,-0.0338851626558393,-9.84506230193631,18.5550464363848,3.38197533809552,20.8420259099524,-0.000311419014084477,0.000701066901408448,-0.000991478873239443
0.228034414981409,-0.0387190373725105,-9.86885810804845,17.04010477741,2.9224457860821,20.6821354263591,-0.000977419014084477,0.000568066901408454,0.000872521126760561
0.237689005511192,-0.0242687440222892,-9.8450943256204,19.3114580306469,2.57847590640175,19.7024991708323,-0.000178419014084469,-0.00049693309859155,-0.000192478873239435
0.252094582708839,-0.0242709141385903,-9.85456761358321,18.2400745031115,3.02622928882282,20.4739950244288,-0.000577419014084479,0.00016906690140845,-0.000458478873239437
0.232849323886919,-0.0339034359410752,-9.86410029525037,18.2400745031115,3.02622928882282,20.4739950244288,-0.0011104190140847,0.000302066901408451,-0.000325478873239443
0.237685522395607,-0.0387025820014099,-9.84510280576338,19.1283737961282,2.12834208284214,20.9302672600855,-0.000577419014084479,0.000701066901408448,-0.000192478873239435
0.247272293532472,-0.0242777290801162,-9.86407203555418,18.0607969813659,2.88892819182612,21.0064297054802,-0.000178419014084469,0.000435066901408453,0.000340521126760557
0.242479991091268,-0.0290848503685756,-9.85458551094597,18.544146767393,3.40225750264923,21.200250084635,-0.00124341901408448,3.60669014084491e-05,-0.000458478873239437
0.252069654248295,-0.0339016180269398,-9.86881760422138,18.1964758590099,3.10735788774139,21.9068906758671,-0.000844419014084469,0.00016906690140845,-0.000591478873239432
0.228040220093232,-0.0146629756480161,-9.86884397467358,18.1964758590099,3.10735788774139,21.9068906758671,-0.000577419014084479,0.00016906690140845,-0.000458478873239437
0.2376652379275539,-0.029088838399012,-9.85934148993777,18.1964758590099,3.10735788774139,21.9068906758671,-0.000444419014084471,-0.000762933098591552,-0.000591478873239432
0.237701753966621,-0.0338853145357766,-9.83560408442517,17.6989404372863,3.26025065728142,21.0388555444721,-4.54190140844746e-05,0.000302066901408451,7.45211267605683e-05
0.237682630617901,-0.0194599566996317,-9.84983994168848,17.6989404372863,3.26025065728142,21.0388555444721,-0.000444419014084471,-0.000363933098591548,-0.000192478873239435
0.232820342286866,-0.0291041334835137,-9.88309024931503,18.708733067848,3.24701504969037,21.7213708563994,-4.54190140844746e-05,-9.79330985915461e-05,-0.00125747887323943
0.242419707710988,-0.0291074638864137,-9.89257008018565,18.708733067848,3.24701504969037,21.7213708563994,-0.00124341901408448,0.00136706690140845,-0.000192478873239435
0.228080789029367,-0.0338904628528119,-9.84036660632479,18.7414320429577,3.18616861532551,20.6466993796437,-0.000311419014084477,-0.000363933098591548,-5.94788732394408e-05
0.232845272563474,-0.0194724242827514,-9.86883926655535,18.4226529608409,2.51759783784126,20.9740034239841,-0.000844419014084469,3.60669014084491e-05,-0.000192478873239435
0.24245100973671,-0.024284543188894,-9.87357546442033,18.9278030855267,2.99036270973515,20.4513743746157,-0.000977419014084477,0.00083506690140845,-0.000325478873239443
0.2280605045613,-0.024276719250414,-9.85460529049919,19.4580515784276,2.77663074629823,20.2447390729285,-0.000844419014084469,3.60669014084491e-05,-0.000591478873239432
0.242478830214377,-0.0338954598802024,-9.85458833726677,19.4580515784276,2.77663074629823,20.2447390729285,0.000354580985915523,0.00016906690140845,-0.000458478873239437
0.23769654152383,-0.0242659171111998,-9.84034588264124,20.1239652900461,2.78132936402318,20.9385683296272,0.000487580985915531,-0.00049693309859155,-5.94788732394408e-05
0.232888163253392,-0.0290786924642937,-9.84035624556496,19.9087033900201,3.35080638166439,21.5132298496811,-0.000311419014084477,3.60669014084491e-05,-0.000325478873239443
0.232889324372739,-0.0242670782305467,-9.84035341865387,19.4215459224107,2.52464439480392,22.0147429105939,0.000354580985915523,0.00016906690140845,-0.00139047887323944
0.227999060061673,-0.029110947001999,-9.89259268664996,18.1713906499191,3.79386195476946,21.5908969564333,-0.0011104190140847,0.000435066901408453,7.45211267605683e-05
0.232866717908433,-0.0242755583735225,-9.85459775606016,18.1713906499191,3.79386195476946,21.5908969564333,-0.000444419014084471,3.60669014084491e-05,-5.94788732394408e-05
0.237652489714565,-0.0194712631634045,-9.86883173054271,20.051995924879,4.19489301434225,21.0230308010429,-0.000178419014084469,0.00016906690140845,-0.00072447887323944
0.228037898096994,-0.0242851993933898,-9.86884962790547,19.8019158696353,2.7586974567544,20.233428748022,-0.000710419014084473,-9.79330985915461e-05,0.000340521126760557
0.232866717908433,-0.0242755583735225,-9.85459775606016,19.0743966333075,3.18851754963163,20.9936142767119,-0.000577419014084479,-0.000896933098591547,7.45211267605683e-05
0.247224760181231,-0.033916912521149,-9.89256537206742,18.3866680508923,3.2243841287193,21.016234926525,-0.0011104190140847,0.00110106690140845,-0.000591478873239432
0.232865556789086,-0.0290871726072695,-9.85460058297125,18.3866680508923,3.2243841287193,21.016234926525,-0.000977419014084477,0.00016906690140845,-0.000325478873239443
0.23764900659898,-0.0339051011425251,-9.86884021068569,18.0177195699286,3.92882044238376,20.7115514408356,8.85809859155207e-05,-0.000363933098591548,-0.000591478873239432
0.24249738378163,-0.0194559686691953,-9.84508396269668,18.5801316773415,2.69547121177118,21.1580185820941,8.85809859155207e-05,-0.000230933098591547,0.000340521126760557
```
